# Copper nanoparticles on controlled pore glass (CPG) as highly efficient heterogeneous catalysts for “click reactions”

**DOI:** 10.1038/s41598-020-77629-3

**Published:** 2020-11-25

**Authors:** Abdolrahim A. Rafi, Ismail Ibrahem, Armando Córdova

**Affiliations:** Department of Natural Sciences, Engineering and Mathematics, Mid Sweden University, 851 70 Sundsvall, Sweden

**Keywords:** Catalyst synthesis, Heterogeneous catalysis

## Abstract

We herein report that supported copper nanoparticles (CuNPs) on commercially available controlled pore glass (CPG), which exhibit high mechanical, thermal and chemical stability as compared to other silica-based materials, serve as a useful heterogeneous catalyst system for 1,3-dipolar cycloadditions (“click” reactions) between terminal alkynes and organic azides under green chemistry conditions. The supported CuNPs-CPG catalyst exhibited a broad substrate scope and gave the corresponding triazole products in high yields. The CuNPs-CPG catalyst exhibit recyclability and could be reuced multiple times without contaminating the products with Cu.

## Introduction

The click chemistry concept invented by Sharpless and co-workers, represent one of the most highly efficient and powerful tools in synthetic organic chemistry^1^. It has been proven to be highly efficient, reliable, wide in scope, high yielding, stereospecific and can proceed under green chemistry conditions. In this context, the copper-(I)salt-catalyzed Huisgen 1,3-dipolar cycloaddition reaction between organic azides and alkynes is a stand out reaction in click chemistry. It works under mild conditions, tolerates several functionalities, recation conditions and have been used for a large varaiety of applications^2–6^. For example, the Cu(I)-catalyzed alkyne-azide click chemistry (CuAAC) reaction have produced 1,2,3-triazole derivatives are employed as antibacterial agents, inhibitors, visualization and labeling of cells as well as several other medicinal chemistry applications^7–12^. It is also an important transformation for functionalization of gel-like^13^ and solid polysaccharides^14^, as well as different materials^15,16^. Copper nanoparticles (CuNPs) have proven to be more efficient catalysts for the CuAAC reaction as compared to copper metal salts, reducing both the catalyst loading and the reaction time^17–22^. In addition, the presence of both CuI/CuII particles on the surface omits the use of ascorbate salts, which is neseccesery to add as a reducing agent for converting the Cu(II) precatalyst to the catalytically active Cu(I) species as well as avoiding aerobic oxidation of the Cu(I) back to a Cu(II) species^2,22^. In addition, the choice of a heterogeneous systems allows for avoidance of contaminating the products with Cu.

Among the different supports that can be prepared with tunable morphology and specific surface functionalization, controlled pore glass (CPG), which belongs to the family of silica-based materials, remains a relatively underdeveloped support within the nanocatalysis field^23–27^. It has several advantages over supports, which have been used for immobilization of metal nanoparticles: cost-effective synthesis, being commercial available with different types of surface functionalities, efficient mass-transfer, high surface area, tunable morphology, producibility in many different shapes , high mechanical, thermal and chemical stability. They are also used in industry for large scale applications. The resulting catalysts are more stable can also exhibit different selectivity as compared to the same types of nanocatalysts supported on mesocellular foam^23,25,28–31^, rice husk derived biosilicates^32^ and nanocellulose foam^33^. Hence, there is a need for further developments of CPG as a support within the nanocatalyst research area. In this context, we have previously assembled heterogeneous palladium nanocatalysts (PdNP)^23,25^ and copper nanocatalysts (CuNP)^24^ on controlled pore glass (CPG), which demonstrated notable levels of performance in catalysis. For example, in our previous work on the development of a CuNP-CPG nanocatalyst we fabricated an efficient catalyst for the aerobic oxidation of alcohols to their corresponding carbonyl compounds^24^. In comparison to other supported CuNP nanocatalysts used for the CuAAC reaction, the use of CPG as the support would allow for all of the above mentioned advantages (e.g. commercial availability, , efficient mass-transfer, high surface area, tunable morphology, controlled pore size, producibility in many different shapes , high mechanical, thermal and chemical stability) as well as being a robust material approved for use in industrial synthesis and biochemistry applications. In fact, CPG is a preffered material in the synthesis and purification of oligonucleotides, RNA and DNA.

**Figure 1:**
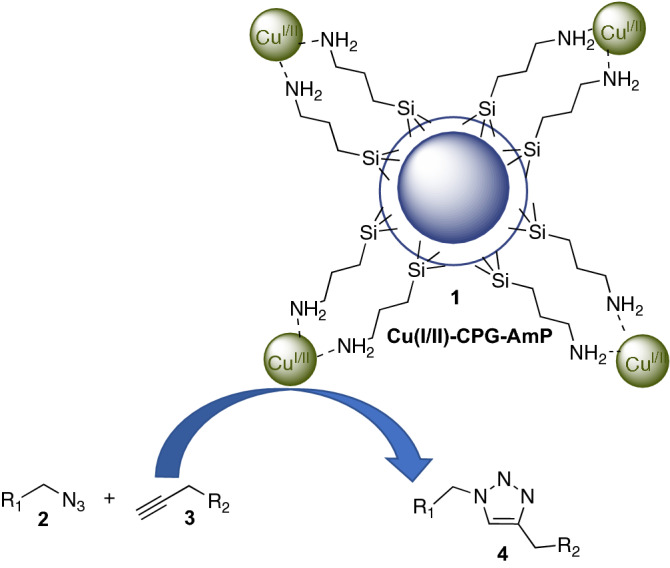
Cu-AmP-CPG (**1**)-catalyzed 1,3-dipolar cycloaddition between azides **2** and propargyl alcohols **3**.

Based on our research interest in development of heterogeneous metal catalysts and the importance of the CuAAC transformation, we began to assemble and investigate a CuNP-CPG nanocatalyst **1** as a heterogeneous catalyst for the selective 1,3-dipolar cycloaddition between azides **2** and alkynes **3** for the synthesis of 1,4-triazoles **4** (Fig. 1).

Herein we disclose that Cu-AmP-CPG serves as a higly efficient and recyclable heterogeneous catalyst for the CuAAC (“click” chemistry) producing the corresponding 1,4-triazoles in high yields. The Cu-AmP-CPG-catalyzed click chemistry reactions proceed under environemtally friendly conditions and no Cu is leached during the process.

## Results and discussion

We began fabricating our heterogeneous catalyst by assembling copper nanoparticles on aminopropyl-(AmP)-functionalized CPG according to our previous procedures. The synthesized Cu-AmP-CPG (**1**) (5 mol%) was next investigated as a heterogeneous catalyst for the 1,3-dipolar cycloaddition reaction between benzyl azide **2a** and propargyl alcohol **3a** in a *t*-BuOH/H_2_O-1:1 mixture (Table 1, entry 1). After 1 h, the initial experiment gave triazole **4a** in > 98% conversion at 70 °C. Encouraged by this result, we continued to probe the recation conditions. The key results are summarized in Table 1.

**Table 1:** Initial examination and model reaction of catalytic 1,3-dipolar cycloaddition of benzyl azide 2a and propargyl alcohol 3a.

Entry^a^	Cu-Amp-CPG **1** (%)	Temp. (°C)	Time (h)	Conv. (%)^b^
1	5	70	0.3	> 98
2	2	70	0.5	> 98
3	1	70	0.7	> 98
4	1	r.t	2	15
5	1	r.t	18	> 98
6	1	50	2	73
7	1	50	2.4	> 98
8	–	70	3	–
9^c^	1	70	0.8	> 98
10^c^	0.5	70	3	> 98
11^c^	0.25	70	4.7	98
12^d^	0.25	70	3	98

^a^Reactions performed using nanocatalyst **1**, **2a** (0.1 mmol) and **3 (**0.15 mmol) in *t*-BuOH/H_2_O; 1:1 (0.5 mL). See SI for details.

^b^Conversion determined by H^1^-NMR analysis using xylene as internal standard.

^c^**2a** (0.1 mmol) and **3a (**0.1 mmol) in *t*-BuOH/H_2_O; 1:1 (0.5 mL).

^d^**2a** (0.1 mmol) and **3a** (0.1 mmol), neat reaction, no solvent.

For example, it was also possible to run the cycloaddition with a significantly lower catalyst loading (**1**, 2 mol %), which gave nearly full conversion within 1 h (entry 2, Table 1). Further reduction of the catalyst loading increased the reaction time (entry 3 and 4, Table 1). Moreover, control experiments revealed that product **4a** was not formed in the absence of nanocatalyst **1** (entry 8, Table 1). To our delight, changing the ratio between **2** and **3a** to 1:1 instead of 1:1.5 gave nearly full conversion to **4a** using very low catalyst loadings (entries 10–12). In this context, the reaction without solvent (neat condition) was also effective at a low catalyst loading of **1** (0.25 mol%) (entry 12, Table 1). The copper nanocatalyst **1**, were recycled from the neat reaction by washing with a fresh *t*-BuOH/H_2_O (1:1) mixture, which is a green solvent mixture. It is important that when the reaction proceed to completion, the desired product **4a** is simply isolated by filtration of **1**, washing (*t*-BuOH/H_2_O (1:1)) and final removal of the solvent. This avoids the use of silica-gel column chromatography techniques and toxic organic solvents. With these results in hand, we selected *t*-BuOH/H_2_O (1:1) as the solvent mixture for probing the reaction for different azides **2** and alkynes **3** over the neat reaction condition due to practical reasons regarding the recycling of the nanocatalyst **1** (Table 2). As depicted in Table 2, a variety of substrates were used to study the scope of the nanocatalyst 1-catalyzed “click” reaction between **2** and **3**. The Cu-AmP-CPG **1**-catalyzed 1,3-dipolar cycloaddition reactions were successful and afforded the corresponding triazoles (**4a**–**4k**) in high yields. Furthermore, we noticed that prolonged reaction times were required in some cases (entries 3 and 8).

**Table 2:** Scope of the 1,3-dipolar cycloaddition reaction catalyzed by Cu-AmP-CPG 1.

Entry	R	R^1^	Cat (mol%)	Prod	Time (h)	Yield [%]^a^
1	Ph	CH_2_OH	0.25		4.7	95
2	Ph	*n*-But	0.25		6	93
3	Ph	Ph	1		48	85
4	Ph	CH_2_CH(CO_2_Me)_2_	0.5		3	92
5	Ph		0.5		7	95
6	Ph		0.5		3	87
7	Ph		0.25		5	92
8	Ph		1		9	93
9	*n*-C_15_H_31_	CH_2_OH	0.25		6	90 (81)^b^
10	*n*-C_15_H_31_	*n*-C_4_H_9_	0.5		7	81
11	*n*-C_15_H_31_		0.25		4	94
12	Ph		0.5		4	94
13	Ph		0.5			
14		CH_2_OH	0.5		24	94

Reactions performed using catalyst **1** (0.25 mol%), **2** (0.4 mmol) and **3** (0.4 mmol) in *t*-BuOH/H_2_O; 1:1 (2 mL) at 70 °C for the time shown. See SI for details.

^a^Isolated yield.

^b^Neat condition (no solvent), **1** (1 mol%), 2 h.

**Figure 2:**
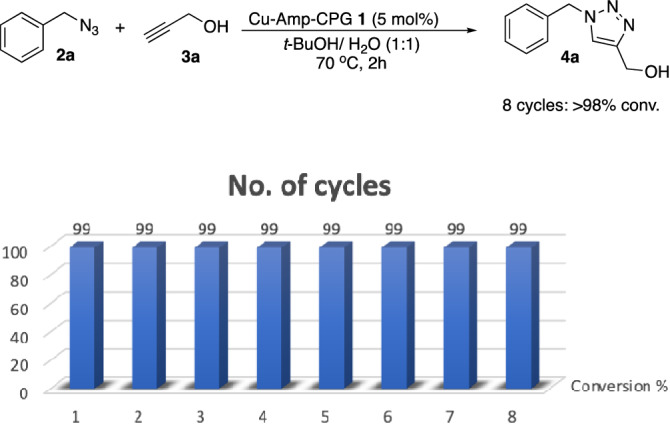
Recycling experiments of Cu-AmP-CPG **1**-catalyzed 1,3-dipolar cycloaddition between benzyl azide **2a** and propargyl alcohol **3a**. Conversion to **4a**.

**Figure 3:**
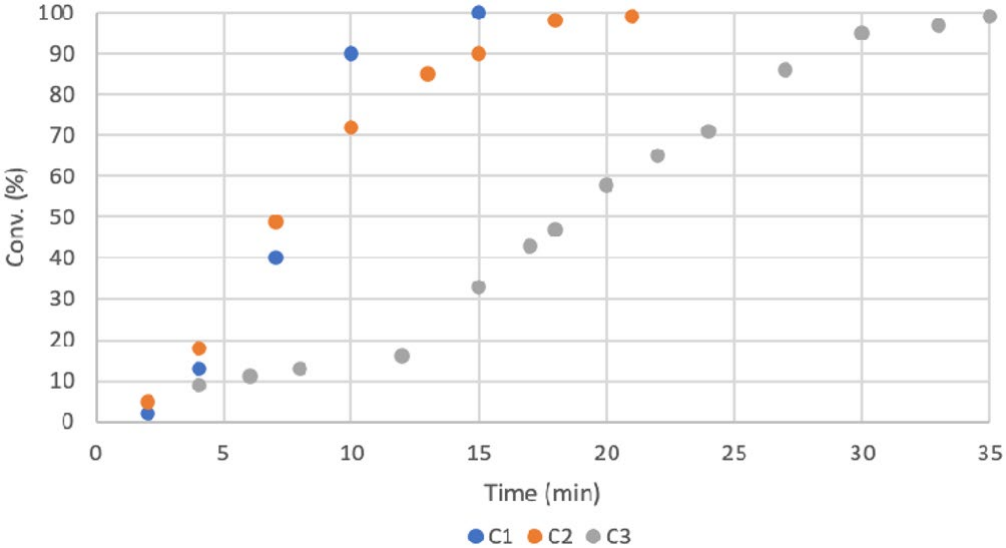
Formation of **4a** for cycles C1 to C3 for the Cu-AmP-CPG **1**-catalyzed 1,3-dipolar cycloaddition between benzyl azide **2a** and propargyl alcohol **3a**. C1 = cycle 1, C2 = cycle 2 and C3 = cycle 3.

Recyclability is an important and crucial factor in developing a heterogeneous catalyst. Thus, we performed recycling experiments of the Cu-AmP-CPG **1** catalyst (Figs. 2 and 3). We found that nanocatalyst **1** afforded product **4a** in excellent conversion over eight consecutive reaction cycles. However, when examining the kinetics of the first three cycles of the Cu-AmP-CPG **1**-catalyzed cycloadditions^34,35^, we found that the recation was completed much faster than the set time of 2 h and that the rate of reaction started to decrease after 2 cycles (Fig. 3). Thus, we had been using more catalyst than needed to achieve full conversion in the allotted time of Table 1. We were also able to recycle catalyst **1** when using differen substrates (Table 3).

**Table 3:** Recycling with different alkynes.

Cycle	R^1^	Product	Time (min)	Conv. (%)
1	CH_2_OH		15	> 99
2	*n*-C_4_H_9_		40	> 99
3	*O*-*p*-MeO-C_6_H_4_		40	> 99

Reactions performed using catalyst **1** (5 mol%), **2** (0.1 mmol) and **3** (0.15 mmol) in *t*-BuOH/H_2_O; 1:1 (0.5 mL) at 70 °C.

The leaching of Cu-AmP-CPG nanocatalyst **1** was also investigated. No leaching was observed. In fact, the elemental analysis of the resulting recation mixture after removal of catalyst **1** had less amount of copper than the reaction performed without catalyst **1** (Table 1, entry 8), which had a negligible amount of copper (*i.e.* less than 10 ppm). Thus, catalyst **1** does not leach. Elemental analysis of the product determined that no Cu was present. In addition, a hot filtration experiment was performed where catalyst **1** was removed when formation of **4a** reached 10% by filtration. Next, the reaction mixtured was allowed to stir for 3 days without the presence of catalyst 1. No formation of product **4a** was observed.

**Figure 4:**
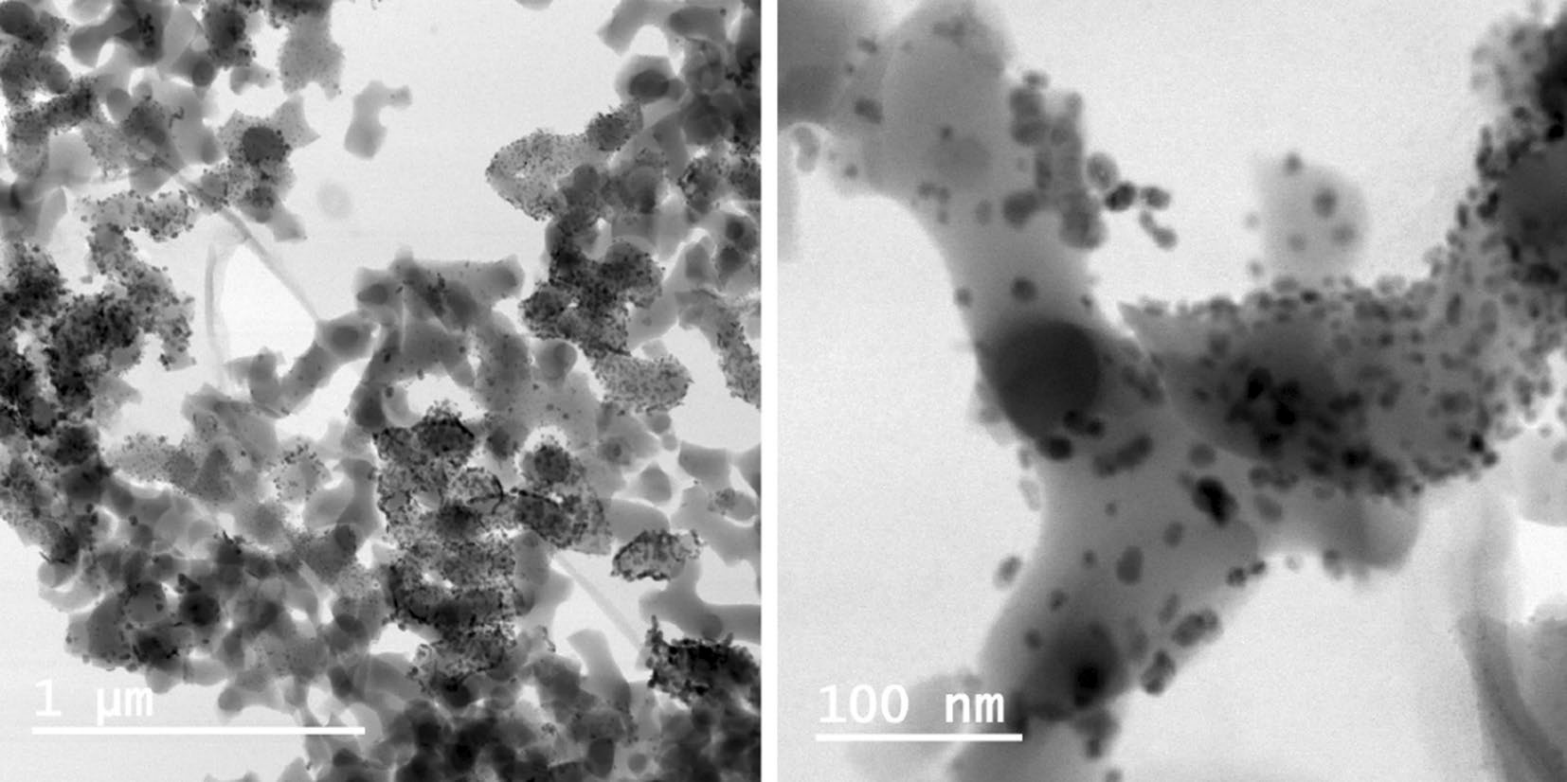
Transmission electron micrographs (TEM) of the mixed valence Cu-AmP-CPG nanocatalyst **1**.

The Cu nanocatalyst was characterized by a number of analytic techniques, including transmission emission microscopy (TEM), scanning electron microscopy (SEM), X-ray photoelectron spectroscopy (XPS) and elemental analysis by inductively coupled plasma optical emission spectrometry (ICP-QES)^24^. The TEM analysis, performed here revealed the Cu-Amp-CPG nanocatalyst (Fig. 4) displayed a well-dispersed and uniform pattern of nanoparticles in the size range 1–20 nm, with an average particle size of 6.4 nm. The total amount of Cu of AmP-CPG **1** was 3.3 wt% as determined by elemental analysis. The XPS analyses revealed that the manufactured Cu-AmP-CPG nanocatalyst **1** had a Cu(II)/Cu(I) atomic ratio of 3.2:1. We also performed XPS analysis on the recovered nanocatalyst **1** after completed reaction, which revealed only Cu(I) in the composition.

## Conclusions

In summary, we have shown that Cu NPs can be successfully immobilized on controlled pore glass to form Cu-AmP-CPG **1** and next used as a heterogeneous copper nanocatalyst for “click” chemistry without the use of ascorbate as a reducing agent. With just a small nanocatalyst loading (0.25–1 mol%), **1**-catalyzed a range of Huisgen 1,3-dipolar cycloadditions with high efficiency exhibiting a broad substrate scope to give the corresponding 1,4-triazole products in high yields. The Cu-AmP-CPG catalyst was simply recycled and multiple reaction cycles. However, we found that the rate of reaction decreases after 2 cycles and this should be taken in account when investigating silica based Cu NPG catalysts^20,21^. The concept of immobilizing noble metals such as Cu on recyclable CPG allows for future development of sustainable and green chemical approaches that should find relevant and sutible future applications in additional chemical transfromations.

## Methods

### General procedure for the catalytic 1,3-dipolar cycloaddition between 2 and 3

A 6-mL microwave vial with a magnetic stir bar was charged with azide compound **2** (1.0 mmol, 1.0 equiv.) and Cu(I/II)-AmP-CPG (5.0 mg, 0.25 mol%). Next, alkyne **3** (1.0 mmol, 1.0 equiv.) and solvent (*t*-BuOH : H_2_O, 1:1, 5 mL) were added to the vial and the resulting mixture was allowed to stir at 70 °C. After 3 h of stirring, the solids were separated by centrifugation and the supernatant was removed. Next, additional solvent (*t*-BuOH : H_2_O, 1:1, 5 mL) was added and the solids were washed and separated by centrifugation again. After removal of the supernatant, the combined solvents were evaporating under reduced pressure to afford the corresponding pure triazole **4**.

### Procedure for recycling of the Cu-AmP-CPG nanocatalyst 1

A 6-mL microwave vial with a magnetic stir bar was charged with azide compound **2a** (0.25 mmol, 1.0 equiv.) and Cu(I/II)-AmP-CPG (10 mg, 5 mol%). Next, alkyne **3a** (0.38 mmol, 1.5 equiv.) and solvent (*t*-BuOH:H_2_O, 1:1, 1.3 mL) were added to the vial and the resulting mixture was allowed to stir at 70 °C. After 2 h of stirring, the solids were separated by centrifugation and the supernatant was removed. Next, additional solvent (*t*-BuOH : H_2_O, 1:1, 5 mL) was added and the solids were washed and separated by centrifugation again. After removal of the supernatant, the combined solvents were evaporating under reduced pressure to afford the corresponding pure triazole **4a**. The solid nanocatalyst **1** that was separated from the reaction mixture by centrifugation was washed once with the reaction solvent *t*-BuOH/H_2_O (1:1) and next re-utilized in the next experimental cycle as described above.

### Procedure for the synthesis of Cu-AmP-CPG (1)

To a suspension of amine functionalized CPG (1.0 g, 1 equiv. amine content) in deionized water (25 mL, pH 9), was added a suspension of copper(II) trifluoromethanesulfonate (Cu(OTf)_2_, 0.3 g, 2 equiv.) in deionized water (20 mL, pH 9) at room temperature. After stirring for 24 h, the prepared Cu(II)-AmP-CPG was transferred to a centrifuge vial (50 mL) and was washed with deionized H_2_O (3 × 35 mL) and acetone (3 × 35 mL) by centrifugation. The washed Cu(II)-AmP-CPG was collected by decantation and dried overnight under reduced pressure. Next, the dry Cu(II)-AmP-CPG was suspended in deionized water (35 mL) and NaBH_4_ (20 equiv.) in deionized water (15 mL) was added slowly at room temperature. After stirring for 45 min, the resulting Cu(I/II)-AmP-CPG nanocatalyst was transferred to a centrifuge vial (50 mL) and was washed with deionized H_2_O (3 × 35 mL) and acetone (3 × 35 mL) by centrifugation and subsequent removal of the supernatant. The washed Cu(I/II)-AmP-CPG was finally dried for 48 h under reduced pressure.

## Supplementary information

Supplementary Information.
