# Supplementary material for: Copper nanoparticles on controlled pore glass (CPG) as highly efficient heterogeneous catalysts for “click reactions”
Source: Sci Rep. 2020 Nov 25;10:20547. doi: 10.1038/s41598-020-77629-3 (PMC7688963; doi:10.1038/s41598-020-77629-3)
Supplement: Supplementary file 1 — Supplementary Information. [file 41598_2020_77629_MOESM1_ESM.pdf]

Copper Nanoparticles on Controlled Pore Glass (CPG) as Highly Efficient Heterogeneous Catalysts for "Click Reactions"

Abdolrahim A. Rafi [‡], *Ismail Ibrahem*[‡], *and Armando Córdova*[*,‡]
[‡]Department of Natural Sciences, Engineering and Mathematics, Mid Sweden University, SE-851 70 Sundsvall, Sweden.

SUPPORTING INFORMATION

General. All chemicals if not noted were purchased from commercial sources. The control pore glass (pore size: 1863 Å, amine content: 93 μmol/g) was obtained from Prime Synthesis Ltd. ^{1}H NMR spectra were recorded on a Bruker Avance 500 (500 MHz) spectrometer. Chemical shifts are reported in ppm from tetramethylsilane with the solvent resonance resulting from incomplete deuterium incorporation as the internal standard ($CDCl_3$: δ 7.26 ppm). Data are reported as follows: chemical shift, multiplicity (s = singlet, d = doublet, q = quartet, br = broad, m = multiplet), and coupling constants (Hz), integration. ^{13}C NMR spectra were recorded on a Bruker Avance 500 (125.8 MHz) spectrometer with complete proton decoupling. Chemical shifts are reported in ppm from tetramethylsilane with the solvent resonance as the internal standard ($CDCl_3$: δ 77.26 ppm). Infrared (IR) spectra were recorded on a Termo Fisher Nicolet 6700 FT-IR spectrometer, ν_{max} in cm^{-1}. Bands are characterized as broad (br), strong (s), medium (m), or weak (w). High resolution mass spectrometry was performed on a Agilent Technologies 6520- Q-TOF ESI-MS (positive mode) at the Mid-Sweden University Mass Spectrometry Facility. Unless otherwise noted, all reactions were performed with distilled solvents under an atmosphere of N_2 in oven-dried (135 ^{o}C) glassware with standard vacuum line techniques. All chemicals used in the synthesis and preparation of the

starting materials and Dimethyl propargylmalonate, were purchased from Aldrich and used as received. For TEM characterization, the Cu nanocatalyst was crushed and diluted with ethanol, and a drop from this suspension was placed on a formvar/carbon coated copper grid and dried under argon. The sample was examined on JEOL JEM 2100F microscope. SEM was performed on JEOL JSM 7401F equipped with cold FEG. Elemental analysis was performed with was performed with inductively coupled plasma-optical emission spectrometry (ICP-OES) by Medac Ltd. (UK) on a Varian Vista MPZ for the quantification of copper. The XPS spectra were collected on a Kratos Axis Ultra DLD electron spectrometer using monochromated Al K_{α} source operated at 150 W. Analyser pass energy of 160 eV for acquiring wide spectra and a pass energy of 20 eV for individual photoelectron lines were used. The surface potential was stabilized by the spectrometer charge neutralization system. The binding energy scale was referenced to the Si 2p line of silica, set at 103.3 eV. Powder sample for the analysis was gently hand-pressed into a pellet directly on a sample holder using clean Ni spatula. The spectra was processed using the Kratos software.

Experimental Procedures

Preparation of Cu(I/II)-Amp-CPG nanoparticles: To a suspension of amine functionalized CPG (1.0 g, 1 equiv. amine content) in deionized water (25 mL, pH 9), was added a suspension of copper(II) trifluoromethanesulfonate ($Cu(OTf)_2$, 0.3 g, 2 equiv.) in deionized water (20 mL, pH 9) at room temperature. After stirring for 24 h, the formed Cu(II)-AmP-CPG was transferred to a centrifuge vial (50 mL) and was washed with deionized H_2O (3 × 35 mL) and acetone (3 × 35 mL), using centrifuge technique. The washed Cu(II)-AmP-CPG was collected by decantation and dried overnight under vacuum.

In the next step, the dry Cu(II)-AmP-CPG was suspended in deionized water (35 mL) and $NaBH_4$ (20 equiv.) in deionized water (15 mL) was added slowly at room temperature. After stirring for 45 min, the resulting Cu(I/II)-AmP-CPG nanocatalyst was transferred to a centrifuge vial (50 mL) and was washed with deionized H_2O (3 × 35 mL) and acetone (3 × 35 mL), using centrifuge technique. The washed Cu(I/II)-AmP-CPG[1] was collected by decantation and dried for 48 h under reduced pressure. The total Cu-content was 3.3 wt% as determined by elemental analysis.

Procedure for the preparation of starting materials

p-methoxyphenyl propargyl ether (3e) [2,3]

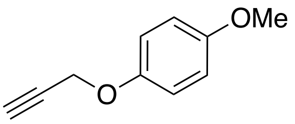

In a round bottom flask, 3.5 mmol (0.443 g, 3.5 mmol) of *p*-methoxyphenol and anhydrous potassium carbonate (1.5 g, 10.8 mmol) were dissolved in 8.0 mL anhydrous DMF. The mixture was heated at 60 °C and stirred for 2 hours under N_2. Next, the mixtures was cooled down to room temperature, and a solution of propargyl bromide (0.650 g, 4.3 mmol, 80% in toluene) was added portion-wise to the solution. The mixture was kept stirring at room temperature for 18 h. Next, the mixture was poured into an ice water and extracted with EtOAc. The organic phase was washed with NaOH 5%, water, brine and dried over Na_2SO_4. Evaporating toluene afforded yellowish oil (0.400 g, 2,47 mmol, 70 %).

^{1}H NMR (500 MHz, CDCl3): δ 6.97 – 6.89 (m, 2H), 6.88 – 6.81 (m, 2H), 4.64 (d, J = 2.3 Hz, 2H), 3.77 (s, 3H), 2.50 (t, J = 2.3 Hz, 1H).

p-nitrophenyl propargyl ether (3f) [2,4]

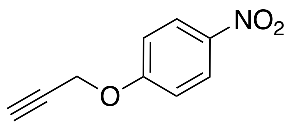

p-nitrophenol (283 mg, 2.0 mmol) was dissolved in 2.5 mL of 0.8 M NaOH and the mixture was heated at 60 °C until a clear solution was formed. Then, tetrabutylammonium bromide (64 mg, 0.2 mmol) was added to the mixture as a phase transfer catalyst. Next, to this solution was added a soloution of propargyl bromide (282 mg, 2.1 mmol, 80 % in toluene) dropwise. After stirring at 60 °C for 24 h, the reaction mixture was cooled to give yellow solids that was collected by filtration. Moreover, the toluene layer was washed with NaOH 5%, water, and dried over Na_2SO_4. Evaporating toluene afforded 3f as pale yellow solids (283 mg, 1.6 mmol, 80 %).

^{1}H NMR (500 MHz, $CDCl_3$): δ 8.26 – 8.20 (m, 2H), 7.10 – 7.03 (m, 2H), 4.80 (d, J = 2.4 Hz, 2H), 2.58 (t, J = 2.4 Hz, 1H).

2-propynyl-2,3,4,6-tetra-*O*-acetyl-β-D-glucopyranoside (3g) [5]

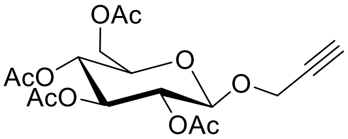

2-propynyl-2,3,4,6-tetra-*O*-acetyl-β-D-glucopyranoside (**3g**) was synthesized through the method described in the literature.[5] First, D-glucose-penta-acetate was synthesized via following procedure: in a 150 ml flask containing 35 mL of acetic anhydride, dextrose (5 g) was added and the reaction mixture was heated to 50 °C. Anhydrous sodium acetate (2.5 g) was added and the mixture was stirred for 2 h. The temperature was increased to 90 °C and the reaction was continued for 3 h. Then, it was cooled and poured with stirring onto 200 mL of ice water. After 3 h, the crystalline material was filtered and crystallized from hot methanol. The product is further purified by recrystallization.

A suspension of D-glucose-penta-acetate (1.5 g, 3,85 mmol) in dry dichloromethane (30 mL) at 0 °C was treated with propargyl alcohol (0.27 mL, 4.6 mmol) and $BF_3 \cdot Et_2O$ (0.72 ml, 5.7 mmol). Next, the reaction temperature was increased to room temperature and the reaction mixture were stirred for additional 3.5 h. Next, anhydrous K_2CO_3 (0.75 g) was added to the mixture and stirred for 30 min at room temperature. The solid were filtered off and washed with dichloromethane. The filtrate (organic phase) was washed with H_2O (2 x 15 mL). The water phase was extracted with dichloromethane. Next, combined organic phase (dichloromethane) washed with brine and dried over Na_2SO_4. Next, the solvent was evaporated under reduced pressure to yield a solid which was crystallized in dichloromethane– petroleum ether affording a white solid (1.19 g, 3.1 mmol, 80 % yield).

^{1}H NMR (500 MHz, CDCl$_3$) δ 5.24 (t, *J* = 9.5 Hz, 1H), 5.10 (t, *J* = 9.7 Hz, 1H), 5.04 – 4.99 (m, 1H), 4.78 (d, *J* = 8.0 Hz, 1H), 4.37 (d, *J* = 2.3 Hz, 2H), 4.28 (dd, *J* = 12.3, 4.6 Hz, 1H), 4.15 (dd, *J* = 12.3, 2.2 Hz, 1H), 3.73 (ddd, *J* = 9.9, 4.5, 2.4 Hz, 1H), 2.47 (t, *J* = 2.1 Hz, 1H), 2.11 – 1.97 (m, 12H); **^{13}C NMR (126 MHz, CDCl$_3$)** δ 170.8, 170.4, 169.6, 169.6, 98.3, 78.2, 75.6, 72.9, 72.1, 71.1, 68.4, 61.9, 56.1, 20.9, 20.8, 20.8, 20.8; **HRMS (ESI$^+$) [M+Na]$^+$** calcd for $C_{17}H_{22}NaO_{10}$: 409.1105, found: 409.1114.

4-(propargyloxy)-coumarin (3h) [6]

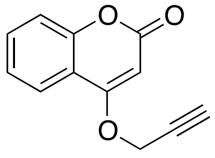

4-(propargyloxy)-coumarin (**3h**) was synthesized through the method described in the literature.[6]

^{1}H NMR (500 MHz, CDCl$_3$) δ 7.82 (dd, *J* = 7.9, 1.5 Hz, 1H), 7.60 – 7.50 (m, 1H), 7.36 – 7.23 (m, 2H), 5.83 (s, 1H), 4.87 (d, *J* = 2.4 Hz, 2H), 2.67 (t, *J* = 2.4 Hz, 1H); **^{13}C NMR**

(126 MHz, $CDCl_3$) δ 164.4, 162.6, 153.5, 132.7, 124.1, 123.2, 116.9, 115.5, 91.8, 78.0, 75.8, 57.0.

2-Propynyl 2,3,4,6-tetra-*O*-acetyl-β-D-galactopyranoside (3i):

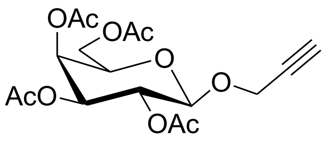

2-Propynyl 2,3,4,6-tetra-*O*-acetyl-β-D-galactopyranoside (**3i**) was synthesized from β-D-galactose pentaacetate through the method described in the literature.[7] Briefly, a suspension of β-D-Galactose pentaacetate (1 g, 2.56 mmol) in dry dichloromethane (20 mL) at 0 °C was treated with propargyl alcohol (0.2 mL, 3.4 mmol) and $BF_3{\cdot}Et_2O$ (0.5 ml, 4.0 mmol). Next, the reaction temperature was increased to room temperature and the reaction mixture were stirred for additional 3.5 h. Next, anhydrous K_2CO_3 (500 mg) was added to the mixture and stirred for 30 min at room temperature. The solid were filtered off and washed with dichloromethane, then, DCM was washed with H_2O (2 x 15 mL). The water phase was extracted with dichloromethane. Next, combined organic phase (dichloromethane) washed with brine and dried over Na_2SO_4. After solvent evaporation under reduced pressure, **3i** was obtained as syrup (900 mg, 2.3 mmol, 91 % yield).

^{1}H NMR (500 MHz, $CDCl_3$) δ 5.40 (dd, *J* = 3.4, 1.0 Hz, 1H), 5.22 (dd, *J* = 10.4, 8.0 Hz, 1H), 5.06 (dd, *J* = 10.4, 3.4 Hz, 1H), 4.74 (d, *J* = 8.0 Hz, 1H), 4.38 (d, *J* = 2.4 Hz, 2H), 4.20 – 4.09 (m, 2H), 3.93 (td, *J* = 6.7, 1.0 Hz, 1H), 2.46 (t, *J* = 2.4 Hz, 1H), 2.15 (s, 3H), 2.07 (s, 3H), 2.05 (s, 3H), 1.99 (s, 3H); **HRMS (ESI$^+$) [M+Na]$^+$** calcd for $C_{17}H_{22}NaO_{10}$: 409.1105, found: 409.1087.

2-Propynyl-β-D-galactopyranoside (3j)

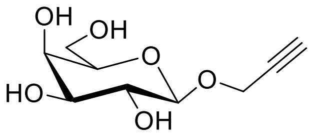

Aqueous ammonia 28% (50 ml) was added to 3i (900 mg, 2.34 mmol) in MeOH–THF (200 ml, 1:1) and the mixture was stirred at r.t. for 2.5 h. The solvent was evaporated and the resultant residue was subjected to the purification by silica gel column chromatography ($CHCl_3$–MeOH (10:1)→$CHCl_3$–MeOH (4:1)) resulting 3j as a white solid (260 mg, 1.18 mmol, 50%).

^{1}H NMR (500 MHz, D_2O): δ 4.48 (dd, *J* = 7.9, 1.1 Hz, 1H), 4.38 (m, 2H), 3.83 (d, *J* = 3.4 Hz, 1H), 3.73 – 3.53 (m, 4H), 3.47 – 3.40 (m, 1H), 2.84 – 2.77 (m, 1H); **^{13}C NMR (126 MHz, D_2O):** δ 101.0, 78.8, 76.1, 75.2, 72.7, 70.5, 68.5, 60.9, 56.4.

1-Azidohexadecane (Cetylazide) (2b) [8]

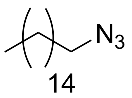

In a 25 mL flask, 3.27 mmol (1.0 g, 3.27 mmol) of alkyl bromide was dissolved in 10.0 mL DMF. To this solution 4.0 mmol (0.260 g, 4 mmol) of solid sodium azide NaN_3. The mixture was heated at 60 °C and stirred for 24 hours under N_2. Next, the mixtures were cooled down to room temperature and the mixture was poured into an ice water and extracted with EtOAc. The organic phase was washed with water and brine and after dried over Na_2SO_4. Evaporating EtOAc afforded 1-azidohexadecane as colorless oil (832 mg, 3.10 mmol, 95%).

^{1}H NMR (500 MHz, $CDCl_3$): δ 3.25 (t, *J* = 7.0 Hz, 2H), 1.66 – 1.55 (m, 2H), 1.43 – 1.18 (m, 26H), 0.88 (t, *J* = 6.9 Hz, 3H).

1-(*tert*-butyl) 2-methyl (*2S,4S*)-4-azidopyrrolidine-1,2-dicarboxylate (2c)

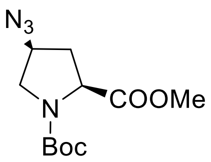

2c was prepared from *trans*-4-Hydroxy-L-proline as described in the litratures.[9, 10] Pure 2c as colorless oil was obtained after column chromatography on silica gel (n-hexane:EtOAc; 2:1).

^{1}H NMR (500 MHz, $CDCl_3$): δ 4.39 (dd, *J* = 8.6, 3.4 Hz, 0.5H), 4.29 (dd, *J* = 8.6, 4.2 Hz, 0.5H), 4.19 – 4.06 (m, 1H), 3.72 (s, 3H), 3.71 – 3.59 (m, 1H), 3.44 (ddd, *J* = 19.6, 11.6, 3.5 Hz, 1H), 2.43 (ddd, *J* = 23.5, 14.1, 8.6 Hz, 1H), 2.13 (d, *J* = 13.4 Hz, 1H), 1.44 (s, 4H), 1.38 (s, 5H); **^{13}C NMR (126 MHz, $CDCl_3$):** δ 172.3, 172.0, 154.0, 153.5, 80.6, 59.3, 58.3, 57.8, 57.4, 52.4, 52.3, 51.3, 50.9, 36.1, 35.2, 28.4, 28.3; **HRMS (ESI$^+$) [M+Na]$^+$** calcd. for $C_{11}H_{18}N_4NaO_4$: 293.122, found: 293.1206.

Typical procedure for 1,3-dipolar cycloaddition

A microvavevial (6-mL) with a magnetic stir bar was charged with the azide compound (1.0 mmol, 1.0 equiv.) and the Cu(I/II)-AmP-CPG catalyst (5.0 mg, 0.25 mol%). Solvent (*t*-BuOH : H_2O, 1:1, 5 mL), and alkyne (1.0 mmol, 1.0 equiv.) were subsequently added to the vial, which was next flushed with nitrogen. After stirring the resulting recation mixture at 70 °C for the time shown in the Tables, the crude products were obtained after catalyst **1** separation and solvent evaporation. Next, silica gel column chromatography gave the corresponding pure products.

(1-benzyl-1*H*-1,2,3-triazol-4-yl)-methanol (4a) [11]

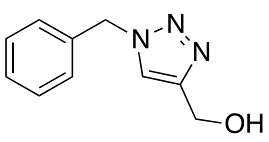

4a was purified on silica gel column chromatography (DCM:MeOH; 10:1) affording a white solid (180 mg, 0.95 mmol, 95% yield); **^{1}H NMR (500 MHz, $CDCl_3$)**: δ 7.44 (s,

1H), 7.41 – 7.33 (m, 3H), 7.30 – 7.26 (m, 2H), 5.51 (s, 2H), 4.76 (s, 2H), 2.48 (s, 1H); **^{13}C NMR (125.8 MHz, CDCl$_3$)**: δ 148.2, 134.6, 129.3, 129.0, 128.3, 121.7, 56.7, 54.4; **HRMS (ESI$^+$) [M+H]$^+$** calcd for $C_{10}H_{12}N_3O$: 190.0975, found: 190.0977.

1-benzyl-4-butyl-1H-1,2,3-triazole (4b) [12]

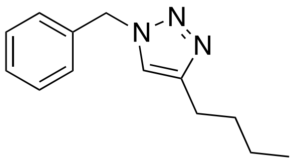

4b was purified on silica gel column chromatography (petroleum ether:EtOAc; 4:1) affording a white solid (200 mg, 0.93 mmol, 93% yield); **^{1}H NMR (500 MHz, CDCl$_3$)**: δ 7.42 – 7.32 (m, 3H), 7.25 (s, 2H), 7.17 (s, 1H), 5.49 (s, 2H), 2.68 (t, *J* = 7.6 Hz, 2H), 1.62 (quintet, *J* = 7.5 Hz, 2H), 1.36 (sextet, *J* = 7.4 Hz, 2H), 0.91 (t, *J* = 7.4 Hz, 3H); **^{13}C NMR (125.8 MHz, CDCl$_3$)**: δ 149.1, 135.2, 129.2, 128.7, 128.1, 120.6, 54.1, 31.7, 25.6, 22.5, 14.0; **HRMS (ESI$^+$) [M+H]$^+$** calcd for $C_{13}H_{18}N_3$: 216.1495, found: 216.1494.

1-benzyl-4-phenyl-1H-1,2,3-triazole (4c) [11]

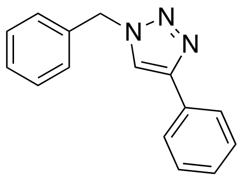

4c was purified on silica gel column chromatography (petroleum ether:EtOAc; 4:1) affording a white solid (209 mg, 0.85 mmol, 85% yield); **^{1}H NMR (500 MHz, CDCl$_3$)**: δ ^{1}H NMR (500 MHz, CDCl$_3$): δH 7.80 (d, *J* = 7.8 Hz, 2H), 7.66 (s, 1H), 7.44 – 7.28 (m, 8H), 5.58 (s, 2H); **^{13}C NMR (125.8 MHz, CDCl$_3$)**: δ 148.6, 134.8, 130.6, 129.3, 129.0, 128.9, 128.3, 128.2, 125.8, 119.6, 54.3; **HRMS (ESI$^+$) [M+H]$^+$** calcd for $C_{15}H_{14}N_3$: 236.1182, found: 236.1180.

(1-benzyl-1H-[1,2,3]-triazo-4-ylmethyl)-malonate dimethyl ester (4d) [13]

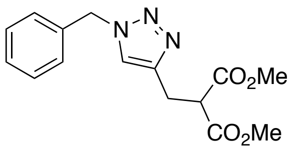

4d was purified on silica gel column chromatography (petroleum ether:EtOAc; 1:1) affording a white solid (280 mg, 0.92 mmol, 92% yield); **^{1}H NMR (500 MHz, CDCl$_3$)**: δ 1H NMR (500 MHz, CDCl3) δ 7.41 – 7.32 (m, 3H), 7.27 (s, 1H), 7.25 – 7.21 (m, 2H), 5.48 (s, 2H), 3.89 (t, *J* = 7.6 Hz, 1H), 3.69 (s, 6H), 3.29 (d, *J* = 7.6 Hz, 2H); **^{13}C NMR (125.8 MHz, CDCl$_3$)**: δ 169.3, 144.5, 134.9, 129.2, 128.9, 128.1, 122.0, 54.2, 52.8, 51.5, 25.2. **HRMS (ESI$^+$) [M+H]$^+$** calcd for $C_{15}H_{18}N_3O_4$: 304.1292, found: 304.1309.

1-benzyl-4-((4-methoxyphenoxy)methyl)-1H-1,2,3-triazole (4e) [11]

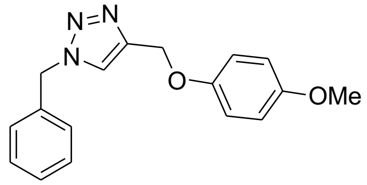

4e was purified on silica gel column chromatography (petroleum ether:EtOAc; 4:1) affording a white solid (280 mg, 0.95 mmol, 95% yield); **^{1}H NMR (500 MHz, CDCl$_3$)**: δ 7.54 (s, 1H), 7.43 – 7.34 (m, 3H), 7.31 – 7.25 (m, 2H), 6.96 – 6.89 (m, 2H), 6.86 – 6.81 (m, 2H), 5.53 (s, 2H), 5.14 (s, 2H), 3.77 (s, 3H); **^{13}C NMR (125.8 MHz, CDCl$_3$)**: δ 154.2, 152.4, 144.9, 134.57, 129.2, 128.8, 128.2, 122.7, 115.9, 114.7, 62.8, 55.7, 54.3; **HRMS (ESI$^+$) [M+H]$^+$** calcd for $C_{17}H_{18}N_3O_2$: 296.1394, found: 296.1391.

1-benzyl-4-((4-nitrophenoxy)methyl)-1H-1,2,3-triazole (4f) [14]

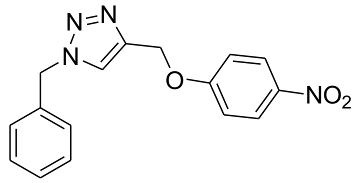

4f was purified on silica gel column chromatography (petroleum ether:EtOAc; 1:1) affording a white solid (270 mg, 0.87 mmol, 87% yield); **^{1}H NMR (500 MHz, CDCl$_3$)**:

δ 8.20 – 8.16 (m, 2H), 7.56 (s, 1H), 7.40 – 7.35 (m, 3H), 7.31 – 7.27 (m, 2H), 7.06 – 7.03 (m, 2H), 5.54 (s, 2H), 5.26 (s, 2H); **^{13}C NMR (125.8 MHz, $CDCl_3$)**: δ 163.2, 143.3, 142.0, 134.3, 129.3, 129.1, 128.3, 126.0, 123.1, 115.0, 62.6, 54.5; **HRMS (ESI^+) $[M+H]^+$** calcd for $C_{16}H_{15}N_4O_3$: 311.1139, found: 311.1137.

1-benzyl-4-(4-methyl-2,3,4,6-tetra-*O*-acetyl-β-D-glucopyranoside)-1H-1,2,3-triazole (4g)

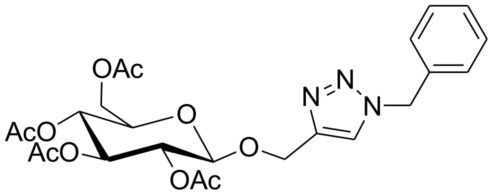

4g was purified on silica gel column chromatography (petroleum ether:EtOAc; 2:3) affording a white solid (475 mg, 0.92 mmol, 92% yield). **IR (neat)**: 3126 (w), 2961 (w), 1736 (s), 1455 (m), 1437 (m), 1366 (m), 1258 (m), 1223 (s), 1155 (m), 1132 (m), 1103 (m), 1040 (s), 959 (m), 907 (m), 725 (m), 692 (m), 597 (m); **^{1}H NMR (500 MHz, $CDCl_3$)**: δ 7.43 (s, 1H), 7.40 – 7.33 (m, 3H), 7.31 – 7.25 (m, 2H), 5.52 (s, 2H), 5.17 (t, J = 9.5 Hz, 1H), 5.07 (t, J = 9.7 Hz, 1H), 5.00 – 4.94 (m, 1H), 4.89 (d, J = 12.7 Hz, 1H), 4.80 (d, J = 12.7 Hz, 1H), 4.64 (d, J = 8.0 Hz, 1H), 4.24 (dd, J = 12.3, 4.7 Hz, 1H), 4.14 – 4.08 (m, 1H), 3.72 – 3.67 (m, 1H), 2.02 (4s, 9H), 1.86 (s, 3H); **^{13}C NMR (125.8 MHz, $CDCl_3$)**: δ 170.8, 170.3, 169.6, 169.5, 144.8, 134.6, 129.3, 129.0, 128.3, 122.8, 100.0, 72.9, 72.0, 71.3, 68.4, 63.1, 61.9, 54.4, 20.9, 20.7, 20.7; **HRMS (ESI^+) $[M+H]^+$** calcd for $C_{24}H_{30}N_3O_{10}$ 520.1926, found: 520.1935.

4-((1-benzyl-1H-1,2,3-triazol-4-yl)methoxy)-2H-chromen-2-one (4h)

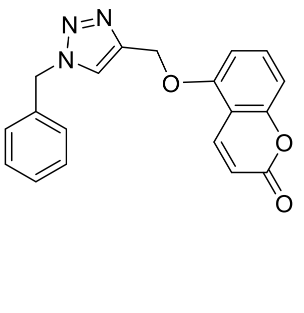

4h was purified on silica gel column chromatography (petroleum ether:EtOAc; 1:2) affording a white solid (310 mg, 0.93 mmol, 93% yield). **IR (neat)**: 3072 (s), 1720 (s), 1623 (s), 1566 (m), 1493 (m), 1457 (m), 1415 (m), 1371 (m), 1273 (m), 1247 (m), 1183 (m), 1139 (m), 1108 (m), 929 (m), 844 (m), 756 (m), 752 (m), 711 (m); **^{1}H NMR (500 MHz, $CDCl_3$)**: δ 7.76 (dd, *J* = 7.9, 1.5 Hz, 1H), 7.62 (s, 1H), 7.56 – 7.51 (m, 1H), 7.43 – 7.36 (m, 2H), 7.33 – 7.29 (m, 2H), 7.25 – 7.20 (m, 1H), 5.83 (s, 1H), 5.58 (s, 2H), 5.31 (s, 2H); **^{13}C NMR (125.8 MHz, $CDCl_3$)**: δ 165.1, 162.8, 153.5, 142.0, 134.3, 132.7, 129.4, 129.2, 128.4, 124.0, 123.4, 123.3, 116.9 , 115.5, 91.3, 62.8, 54.6; **HRMS (ESI^+) $[M+H]^+$** calcd for $C_{19}H_{16}N_3O_3$: 334.1186, found: 334.1212.

(1-hexadecyl-1H-1,2,3-triazol-4-yl)methanol (4i) [15]

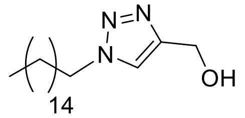

4i was purified on silica gel column chromatography (petroleum ether:EtOAc; 1:2) affording a white solid (290 mg, 0.90 mmol, 90% yield) of as white solid. **^{1}H NMR (500 MHz, $CDCl_3$)**: δ 7.53 (s, 1H), 4.79 (s, 2H), 4.33 (t, *J* = 7.2 Hz, 2H), 2.55 (s, 1H), 1.95 – 1.83 (m, 2H), 1.34 – 1.17 (m, 26H), 0.87 (t, *J* = 6.9 Hz, 3H); **^{13}C NMR (125.8 MHz, $CDCl_3$)**:

δ 147.5, 121.6, 56.9, 50.6, 32.1, 30.5, 29.8, 29.8, 29.8, 29.8, 29.7, 29.7, 29.5, 29.5, 29.1, 2 6.6, 22.8, 14.3; **HRMS (ESI^+) $[M+H]^+$** calcd for $C_{19}H_{38}N_3O$: 324.3009, found: 324.3033.

4-butyl-1-hexadecyl-1H-1,2,3-triazole (4j)

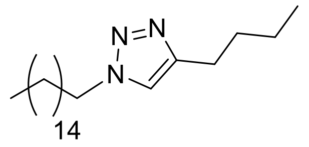

4j was purified on silica gel column chromatography (petroleum ether:EtOAc; 4:1) affording a white solid (280 mg, 0.81 mmol, 81% yield). **IR (neat)**: 3122 (m), 3073 (m), 2954 (s), 2914 (s), 2871 (s), 2847 (s), 2360 (m), 2342 (m), 1555 (m), 1469 (m), 1376 (m), 1214 (m), 1155 (m), 1063 (m), 1031 (m), 854 (m), 719 (m); **^{1}H NMR (500 MHz, $CDCl_3$)**: δ 7.24 (s, 1H), 4.30 (t, *J* = 7.3 Hz, 2H), 2.71 (t, *J* = 7.6, 2H), 1.92 – 1.83 (m, 2H), 1.65 (quintet, *J* = 7.7 Hz, 2H), 1.38 (sextet, *J* = 7.5, 2H), 1.33 – 1.21 (m, 26H), 0.93 (t, *J* = 7.4 Hz, 3H), 0.88 (t, *J* = 6.9 Hz, 3H); **^{13}C NMR (125.8 MHz, $CDCl_3$)**: δ 148.5, 120.5, 50.4, 32.1 , 31.7, 31.1, 30.5, 29.8, 29.8, 29.7, 29.7, 29.5, 29.5, 29.2, 26.7, 25.5, 22.8, 22.5, 14.3, 14.0; **HRMS (ESI$^+$) [M+H]$^+$** calcd for $C_{22}H_{44}N_3$: 350.3530, found: 350.3533.

1-hexadecyl-(4-methyl-2,3,4,6-tetra-*O*-acetyl-β-D-glucopyranoside)-1,2,3-triazol (4k)[15]

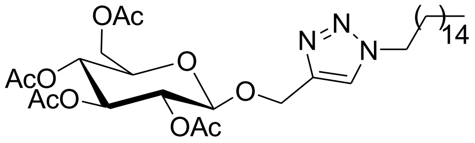

4k was purified on silica gel column chromatography (petroleum ether:EtOAc; 1:2) affording a white solid (620 mg, 0.94 mmol, 94% yield). **^{1}H NMR (500 MHz, $CDCl_3$)**: 7.49 (s, 1H), 5.20 (t, *J* = 9.5 Hz, 1H), 5.10 (t, *J* = 9.7 Hz, 1H), 5.05 – 4.99 (m, 1H), 4.94 (d, *J* = 12.6 Hz, 1H), 4.82 (d, *J* = 12.6 Hz, 1H), 4.68 (d, *J* = 8.0 Hz, 1H), 4.33 (t, *J* = 7.2 Hz, 2H), 4.27 (dd, *J* = 12.3, 4.7 Hz, 1H), 4.15 (dd, *J* = 12.3, 2.2 Hz, 1H), 3.77 – 3.71 (m, 1H), 2.09, 2.02, 1.99, 1.98 (4s, 12H), 1.92 – 1.85 (m, 2H), 1.36 – 1.22 (m, 26H), 0.87 (t, *J* = 6.8 Hz, 3H); **^{13}C NMR (125.8 MHz, $CDCl_3$)**: δ 170.8, 170.4, 169.6, 169.5, 144.2, 122.7, 100.0, 72.9, 72.1, 71.4, 68.5, 63.2, 62.0, 50.6, 32.1, 30.5, 29.8, 29.8, 29.8, 29.7, 29.7, 29.5, 29.5, 29.2, 26.7, 22.8, 20.9, 20.8, 20.8, 14.3; **HRMS (ESI$^+$) [M+H]$^+$** calcd for $C_{33}H_{56}N_3O_{10}$: 654.3960, found: 654.3976.

1-benzyl-4-(4-methyl-2,3,4,6-tetra-*O*-acetyl-β-D-galactopyranoside)-1H-1,2,3-triazole (4l)

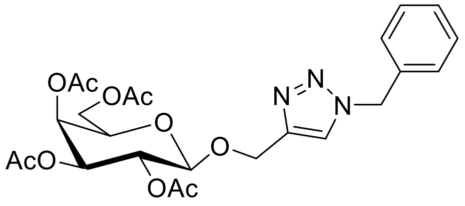

4l was purified on silica gel column chromatography (petroleum ether:EtOAc; 1:1) affording a yellowish oil (500 mg, 0.94 mmol, 94% yield). **^{1}H NMR (500 MHz, $CDCl_3$):** δ 7.43 (s, 1H), 7.39 – 7.27 (m, 5H), 5.51 (s, 2H), 5.37 (d, *J* = 3.0 Hz, 1H), 5.18 (dd, *J* = 10.3, 8.0 Hz, 1H), 4.98 (dd, *J* = 10.4, 3.4 Hz, 1H), 4.91 (d, *J* = 12.5 Hz, 1H), 4.80 (d, *J* = 12.6 Hz, 1H), 4.62 (d, *J* = 7.9 Hz, 1H), 4.12 (d, *J* = 6.6 Hz, 2H), 3.91 (t, *J* = 6.5 Hz, 1H), 2.12 (s, 3H), 2.03 (s, 3H), 1.96 (s, 3H), 1.85 (s, 3H); **^{13}C NMR (126 MHz, $CDCl_3$):** δ 170.5, 170.3, 170.2, 169.6, 144.9, 134.6, 129.3, 129.0, 128.3, 122.8, 100.5, 71.0, 68.9, 67.2, 63.1, 61.4, 54.4, 29.8, 20.8, 20.8, 20.7, 20.7; **HRMS (ESI$^+$) [M+H]$^+$** calcd for $C_{24}H_{30}N_3O_{10}$: 520.1926, found: 520.1915.

(1-benzyl-1,2,3-triazol-4-yl)methyl-β-D-galactopyranoside (4m)

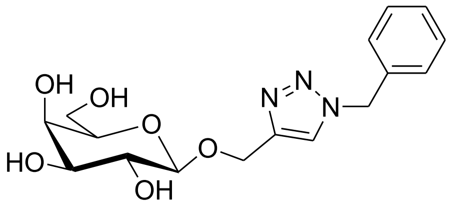

4m was purified on silica gel column chromatography ($CHCl_3$:MeOH; 4:1) affording a white solid (315 mg, 0.90 mmol, 90% yield). **^{1}H NMR (500 MHz, MeOD):** δ 8.00 (s, 1H), 7.45 – 7.27 (m, 5H), 5.59 (s, 2H), 4.96 (d, *J* = 12.5 Hz, 1H), 4.77 (d, *J* = 12.5 Hz, 1H), 4.32 (d, *J* = 7.6 Hz, 1H), 3.83 – 3.81 (m, 1H), 3.73 (ddd, *J* = 16.5, 11.4, 6.1 Hz, 2H), 3.56 – 3.50 (m, 2H), 3.45 (dd, *J* = 9.7, 3.4 Hz, 1H), 3.32 – 3.31 (m, 2H); **^{13}C NMR (126 MHz, MeOD):** δ 146.3, 136.8, 130.0, 129.6, 129.2, 125.3, 104.3, 76.8, 74.9, 72.4, 70.3,

63.0, 62.6, 55.0; **HRMS (ESI$^+$) [M+H]$^+$** calcd for $C_{16}H_{22}N_3O_6$: 352.1503, found: 352.1501.

(2S,4S)-1-tert-butyl 2-methyl 4-(4-(hydroxymethyl)-1H-1,2,3-triazol-1-yl)pyrrolidine-1,2-dicarboxylate (4n)

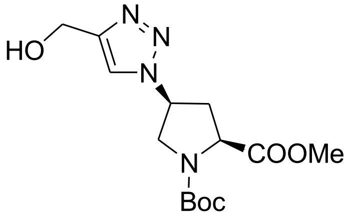

4n was purified on silica gel column chromatography (EtOAc) affording a colorless oil (305 mg, 0.94 mmol, 94% yield). **^{1}H NMR (500 MHz, CDCl$_3$):** δ 7.70 (s, 1H), 5.13 (s, 1H), 4.75 (s, 2H), 4.44 (d, *J* = 37.7 Hz, 1H), 4.13 (dd, *J* = 11.4, 7.3 Hz, 1H), 3.83 (s, 1H), 3.69 (s, 3H), 3.30 (s, 1H), 2.95 – 2.87 (m, 1H), 2.59 (d, *J* = 50.9 Hz, 1H), 1.41 – 1.45 (2s, 9H); **^{13}C NMR (126 MHz, CDCl$_3$):** δ 172.2, 153.9, 153.4, 148.4, 121.0, 81.2, 58.1, 57.8, 57.5, 57.3, 56.2, 52.6, 52.4, 51.7, 51.2, 36.1, 35.3, 28.2; **HRMS (ESI$^+$) [M+H]$^+$** calcd for $C_{14}H_{23}N_4O_5$: 327.1663, found: 327.1651.

Procedure for recycling of the Cu-AmP-CPG nanocatalyst 1 (with different alkyne substrates).

A 6-mL microwave vial with a magnetic stir bar was charged with azide compound **2a** (0.1 mmol, 1.0 equiv.) and Cu(I/II)-AmP-CPG **1** (10.0 mg, 5 mol%). Next, solvent (*t*-BuOH:H_2O, 1:1, 0.5 mL) and alkyne **3** (0.15 mmol, 1.5 equiv.) was added and the vial was flushed with nitrogen. After stiring the reaction mixture at 70 °C, the catalyst was separated by centrifugation and the supernatant collected. The remaining nanocatalyst 1 was washed with acetone (3 x 5 mL) by centrifugation and next dried under reduced pressure. The recovered catalyst **1** was next used for another reaction cycle as described above.

References

1. Ibrahem, I.; Iqbal, M. N.; Verho, O.; Eivazihollagh, A.; Olsén, P.; Edlund, H.; Tai, C.-W.; Norgren, M.; Johnston, E. V. Copper Nanoparticles on Controlled Pore Glass and TEMPO for the Aerobic Oxidation of Alcohols. *ChemNanoMat* **4**, 71-75 (2018).
2. Pal, M., Parasuraman, K. & Yeleswarapu, K. R. Palladium-Catalyzed Cleavage of O/N-Propargyl Protecting Groups in Aqueous Media under a Copper-Free Condition1. *Organic Letters* **5**, 349-352 (2003).
3. Schimler, S. D., Hall, D. J. & Debbert, S. L. Anticancer (hexacarbonyldicobalt)propargyl aryl ethers: Synthesis, antiproliferative activity, apoptosis induction, and effect on cellular oxidative stress. *Journal of Inorganic Biochemistry* **119**, 28-37 (2013).
4. Agag, T. & Takeichi, T. Novel Benzoxazine Monomers Containing p-Phenyl Propargyl Ether: Polymerization of Monomers and Properties of Polybenzoxazines. *Macromolecules* **34**, 7257-7263 (2001).
5. Singh, R. & Varma, A. J. Towards biodegradable elastomers: green synthesis of carbohydrate functionalized styrene–butadiene–styrene copolymer by click chemistry. *Green Chemistry* **14**, 348-356 (2012).
6. Thasnim, P. & Bahulayan, D. Click-on fluorescent triazolyl coumarin peptidomimetics as inhibitors of human breast cancer cell line MCF-7. *New Journal of Chemistry* **41**, 13483-13489 (2017).
7. Mereyala, H. B. & Gurrala, S. R. A highly diastereoselective, practical synthesis of allyl, propargyl 2,3,4,6-tetra-O-acetyl-β-d-gluco, β-d-galactopyranosides and allyl, propargyl heptaacetyl-β-d-lactosides. *Carbohydrate Research* **307**, 351-354 (1998).
8. Mishra, R., Pandey, S., Trivedi, S., Pandey, S. & Pandey, P. S. Synthesis and properties of l-valine based chiral long alkyl chain appended 1,2,3-triazolium ionic liquids. *RSC Advances* **4**, 33478-33488 (2014).
9. Gong, J., Gong, Y. & Xu, W. Synthesis and crystal structure of cis-4-azido-L-proline methyl ester hydrochloride. *Journal of Chemical Research* **2009**, 668-670 (2009).
10. Abraham, D. J., Mokotoff, M., Sheh, L. & Simmons, J. E. Design, synthesis, and testing of antisickling agents. 2. Proline derivatives designed for the donor site. *Journal of Medicinal Chemistry* **26**, 549-554 (1983).
11. Buckley, B. R., Dann, S. E., Harris, D. P., Heaney, H. & Stubbs, E. C. Alkynylcopper(i) polymers and their use in a mechanistic study of alkyne–azide click reactions. *Chemical Communications* **46**, 2274-2276 (2010).
12. Wang, D. *et al*. Solvent-free synthesis of 1,4-disubstituted 1,2,3-triazoles using a low amount of Cu(PPh3)2NO3 complex. *Green Chemistry* **12**, 2120-2123 (2010).
13. Tasca, E., La Sorella, G., Sperni, L., Strukul, G. & Scarso, A. Micellar promoted multi-component synthesis of 1,2,3-triazoles in water at room temperature. *Green Chemistry* **17**, 1414-1422 (2015).
14. Jiang, Y. *et al*. Metallic copper wire: a simple, clear and reusable catalyst for the CuAAC reaction in supercritical carbon dioxide. *RSC Advances* **5**, 73340-73345 (2015).
15. Tyagi, M. & Kartha, K. P. R. Synthesis of glycotriazololipids and observations on their self-assembly properties. *Carbohydrate Research* **413**, 85-92 (2015).

SEM images

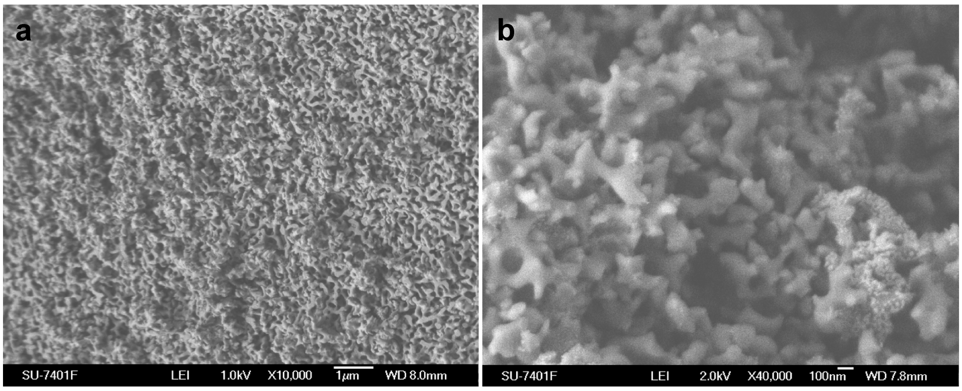

Figure S1. SEM images of Cu-AmP-CPG (1) with (a) 1 μm scale bar, and (b) 100 nm scale bar.

XPS spectra

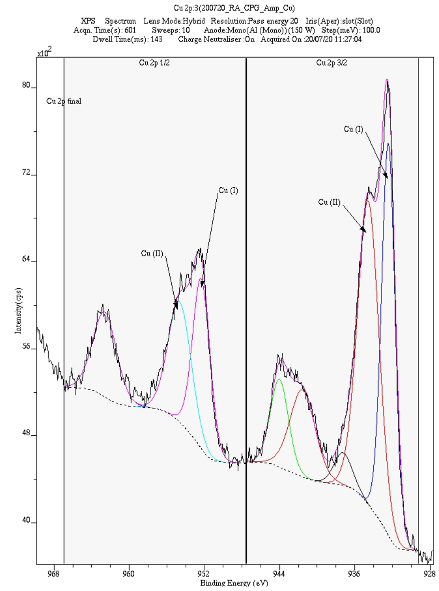

Figure S2. XPS spectrum of Cu-AmP-CPG nanocatalyst 1.

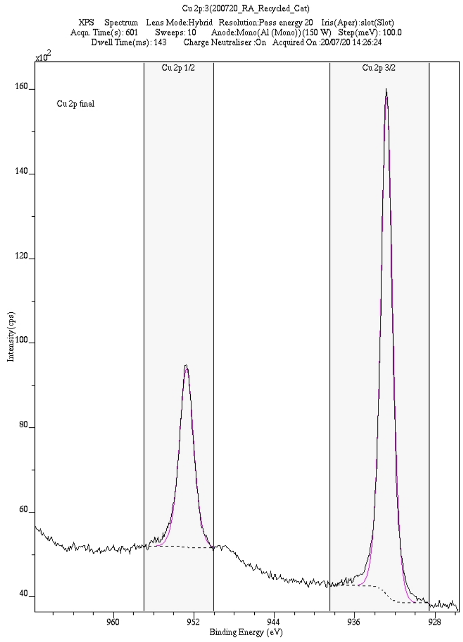

Figure S3. XPS spectrum of nanocatalyst **1** after first ceycle.

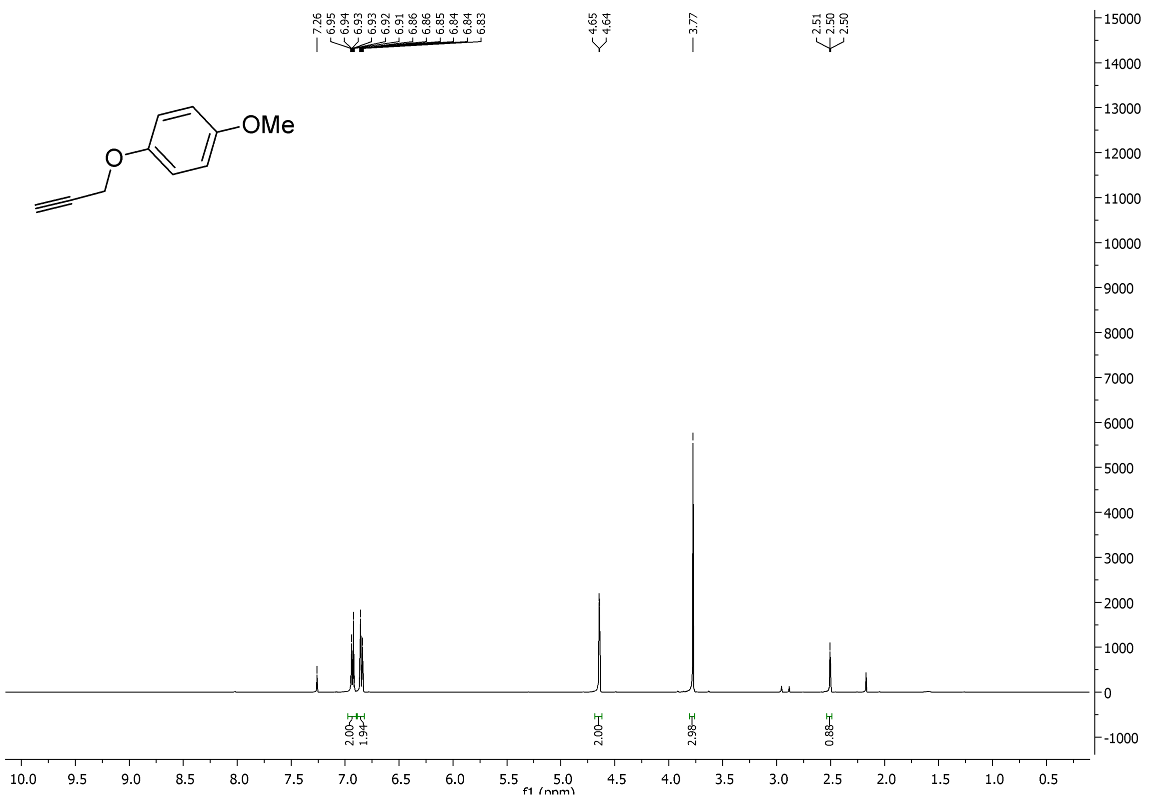

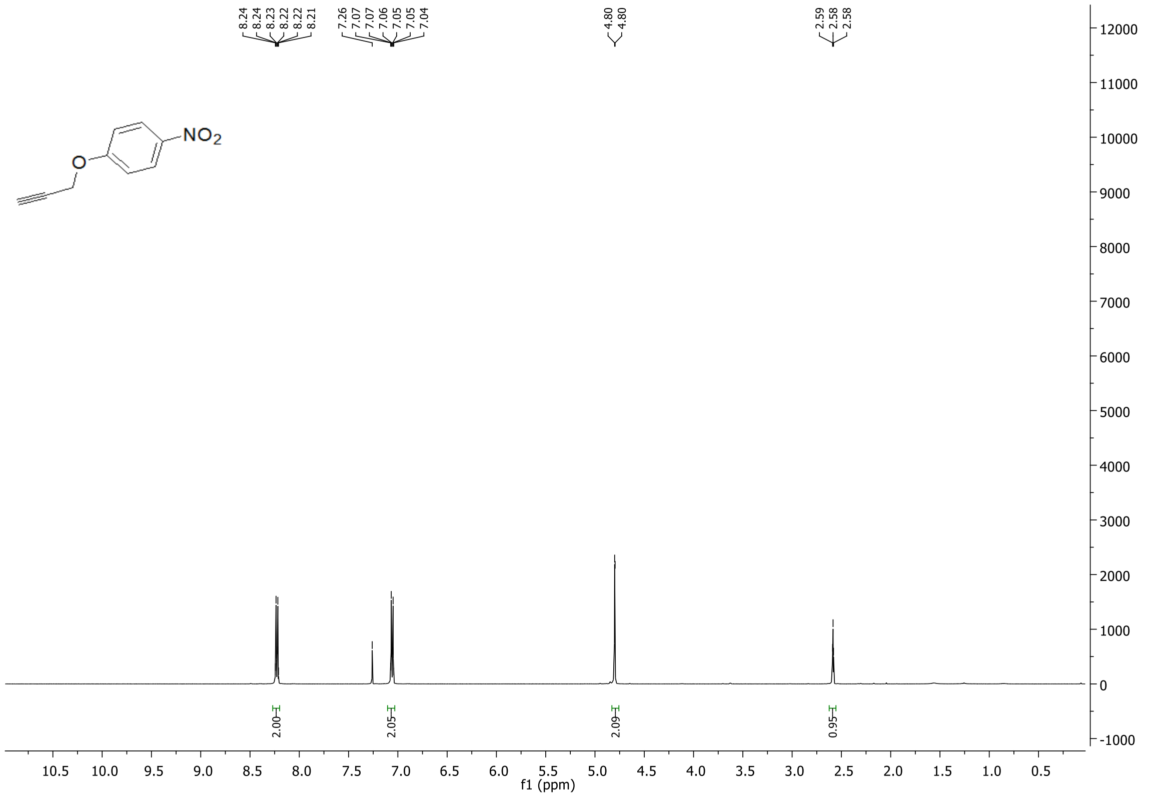

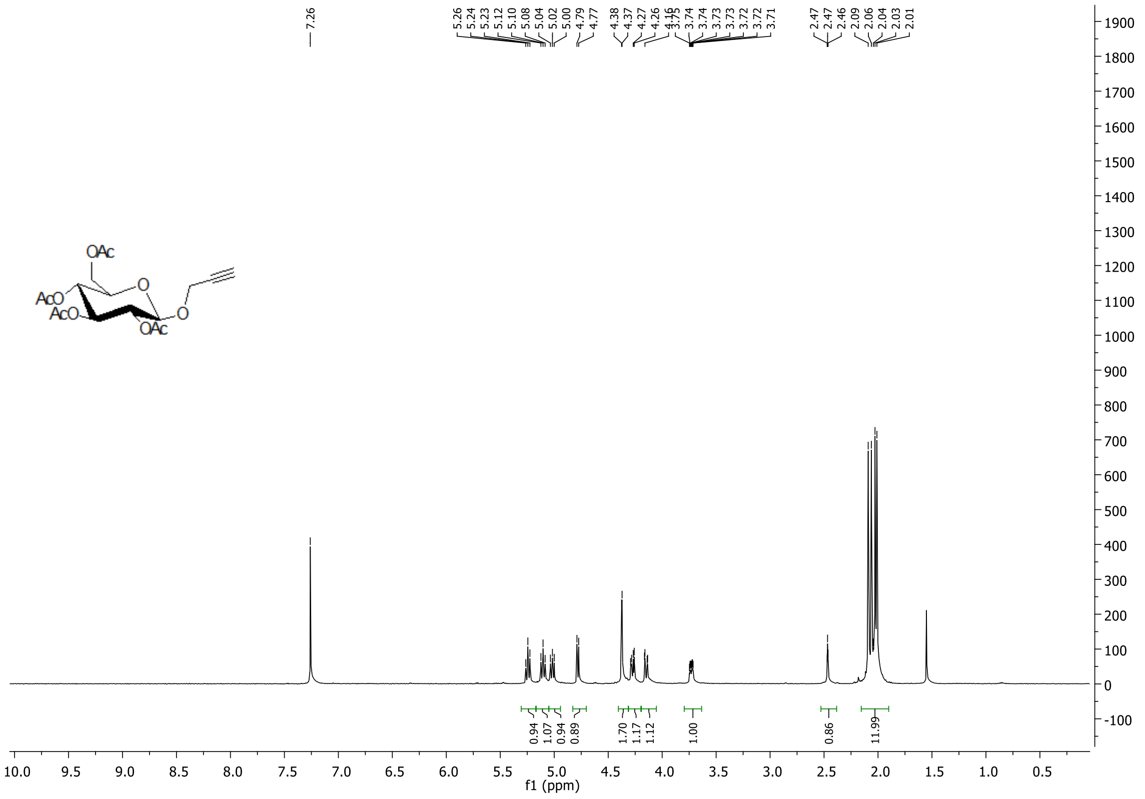

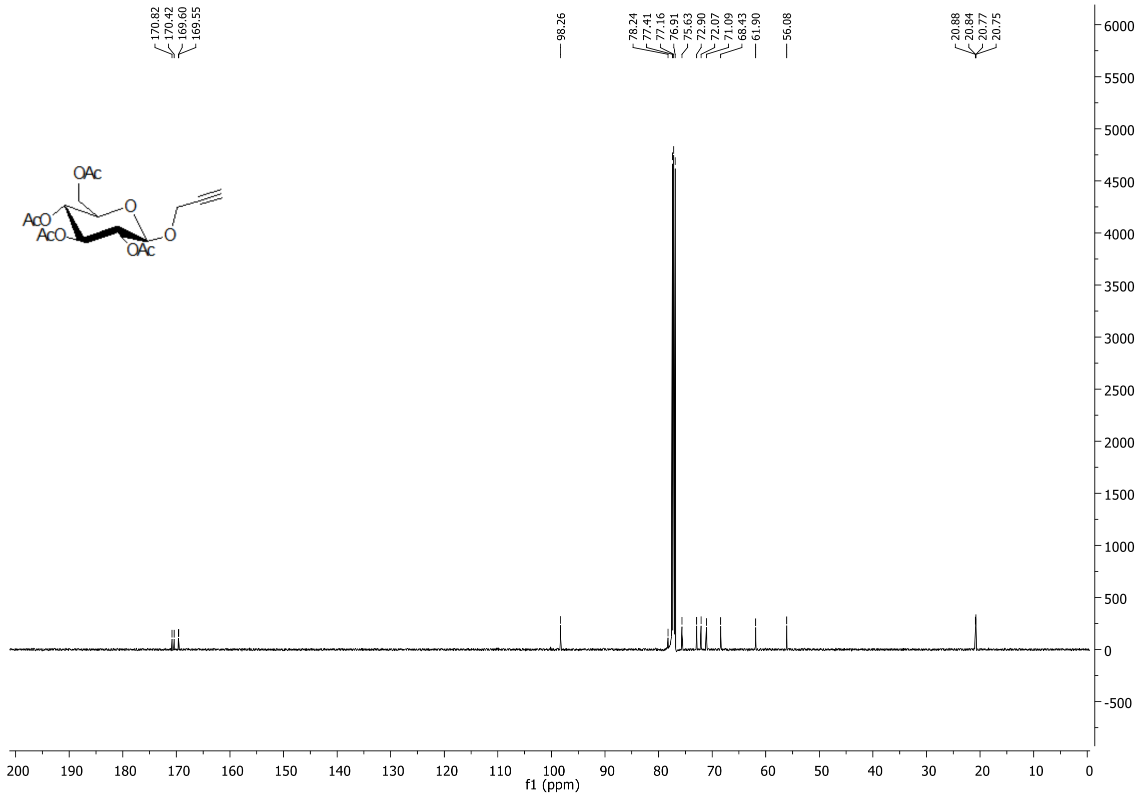

Qualitative Compound Report

**Data File**	rr-105.d	**Sample Name**	rr-105
**Sample Type**	Sample	**Position**	Vial 1
**Instrument Name**	QTOF	**User Name**	QTOF-PC\admin
**Acq Method**	ACgroup_new.m	**Acquired Time**	2019-02-28 16:23:57
**IRM Calibration Status**	Success	**DA Method**	szfg123.m
**Comment**			

**Acquisition SW**	6200 series TOF/6500 series
**Version**	Q-TOF B.05.00 (B5042.2)

Compound Table

Compound Label	RT	Mass	Abund	Formula	Tgt Mass	Diff (ppm)	MFG Formula	DB Formula
Cpd 1: C17 H22 O10	0.584	386.1222	31827	C17 H22 O10	386.1213	2.3	C17 H22 O10	C17 H22 O10

Compound Label	*m/z*	RT	Algorithm	Mass
Cpd 1: C17 H22 O10	409.1114	0.584	Find By Formula	386.1222

MS Zoomed Spectrum

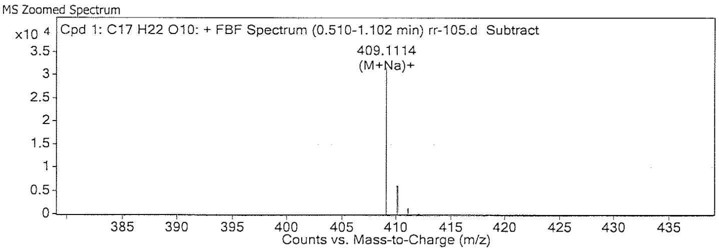

MS Spectrum Peak List

m/z	z	Abund	Formula	Ion
409.1114	1	31827.38	C17H22NaO10	(M+Na)+
410.1146	1	6094.54	C17H22NaO10	(M+Na)+
411.1167	1	1280.41	C17H22NaO10	(M+Na)+
412.1216	1	143.85	C17H22NaO10	(M+Na)+

--- End Of Report ---

Mass spectrum of 3g

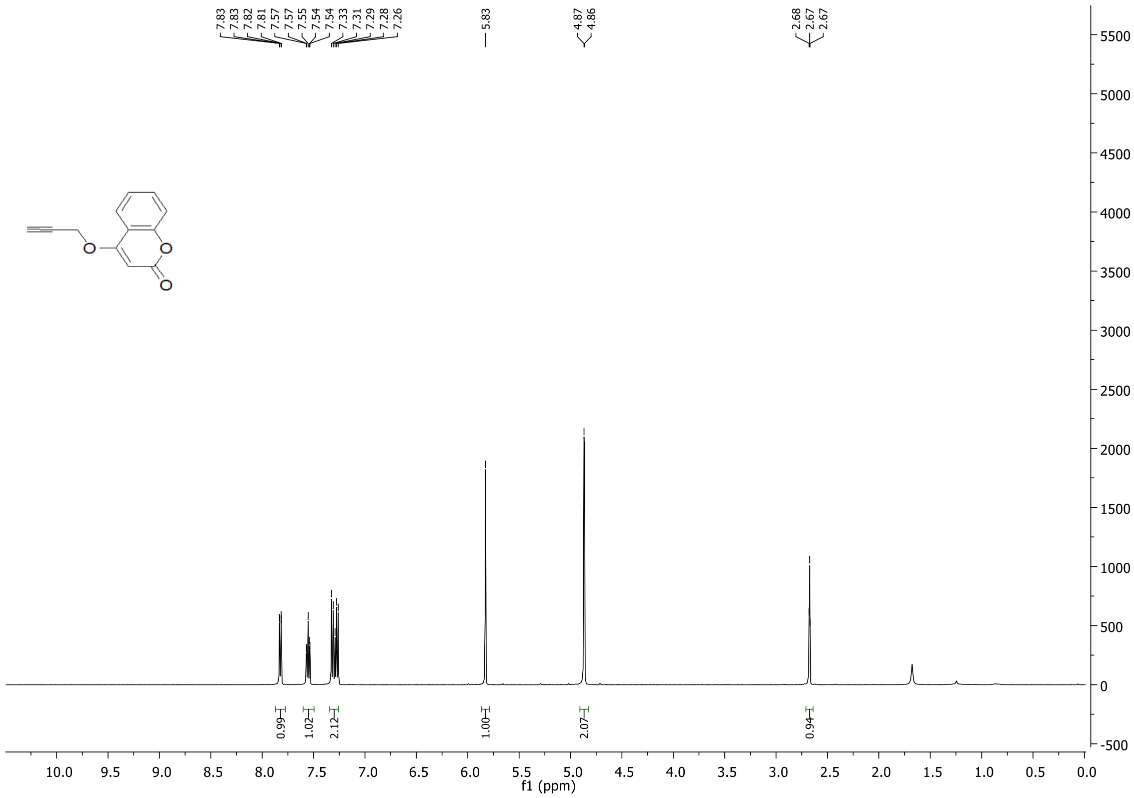
7.83
7.83
7.82
7.81
7.57
7.57
7.55
7.54
7.54
7.33
7.31
7.29
7.28
7.26
5.83
4.87
4.86
2.68
2.67
2.67
0.99
1.02
2.12
1.00
2.07
0.94
f1 (ppm)

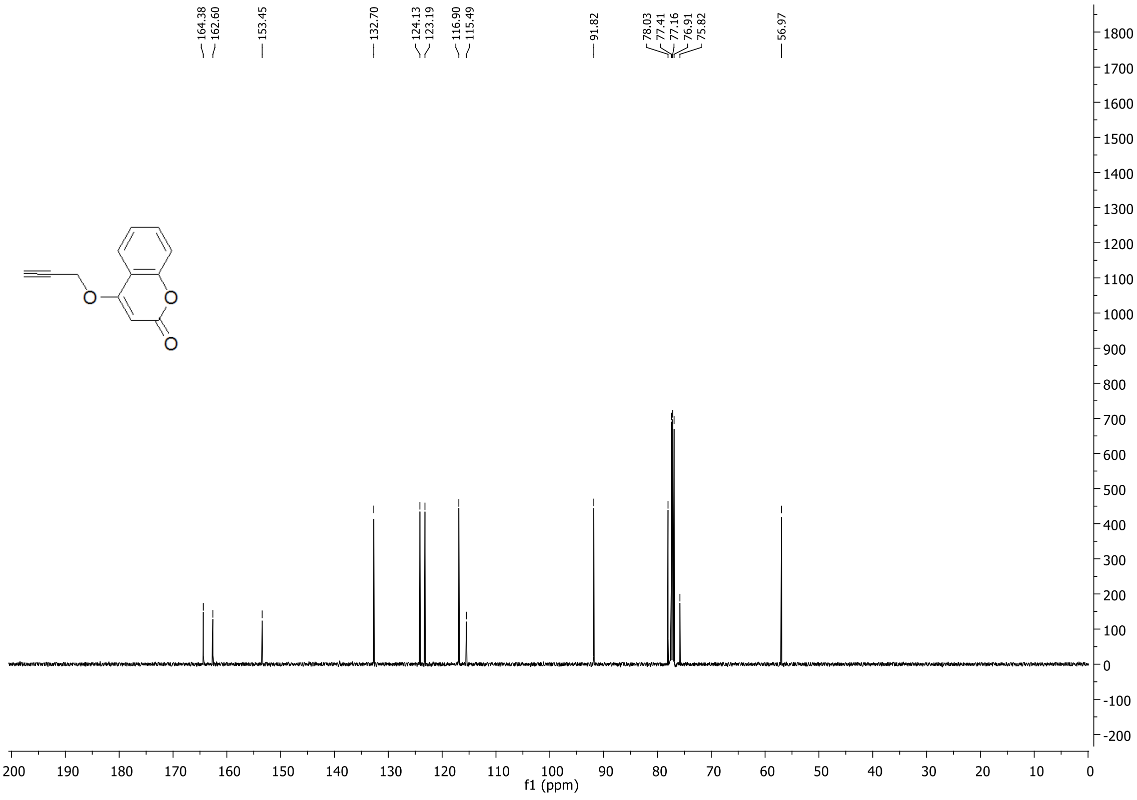
164.38
162.60
153.45
132.70
124.13
123.19
116.90
115.49
91.82
78.03
77.41
77.16
76.91
75.82
56.97
f1 (ppm)

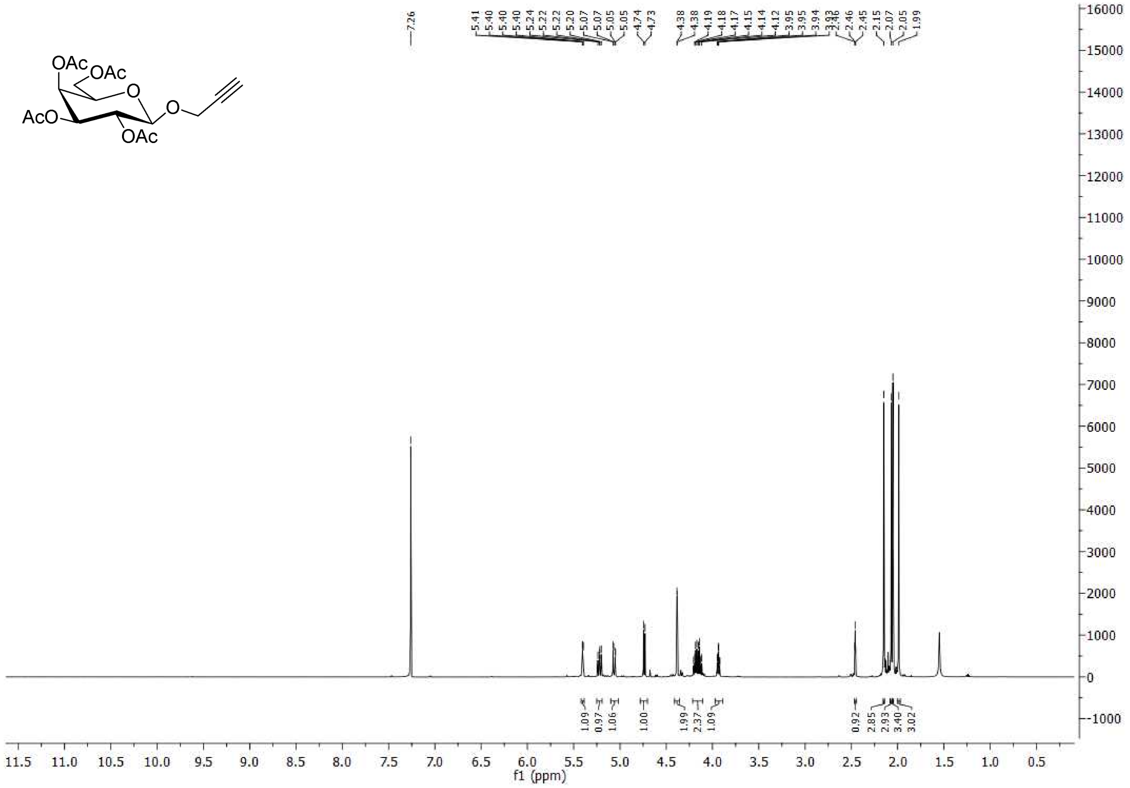

Qualitative Compound Report

**Data File**	rr-1-146.d	**Sample Name**	rr-1-146
**Sample Type**	Sample	**Position**	Vial 1
**Instrument Name**	QTOF	**User Name**	QTOF-PC\admin
**Acq Method**	ACgroup_new.m	**Acquired Time**	2020-07-28 12:19:36
**IRM Calibration Status**	Success	**DA Method**	Default.m
**Comment**			

Acquisition SW Version 6200 series TOF/6500 series Q-TOF B.05.00 (B5042.2)

Compound Table

Compound Label	RT	Mass	Abund	Formula	Tgt Mass	Diff (ppm)	MFG Formula	DB Formula
Cpd 1: C17 H22 O10	1.003	386.1196	40160	C17 H22 O10	386.1213	-4.41	C17 H22 O10	C17 H22 O10

Compound Label	*m/z*	RT	Algorithm	Mass
Cpd 1: C17 H22 O10	425.0838	1.003	Find By Formula	386.1196

MS Zoomed Spectrum

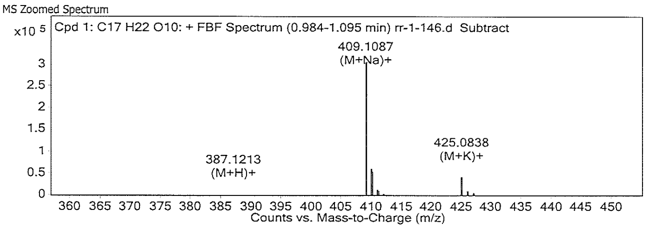

MS Spectrum Peak List

m/z	z	Abund	Formula	Ion
387.1213	1	497.81	C17H23O10	(M+H)+
388.1252	1	107.66	C17H23O10	(M+H)+
409.1087	1	307321.5	C17H22NaO10	(M+Na)+
410.1118	1	55479.63	C17H22NaO10	(M+Na)+
411.114	1	10917.18	C17H22NaO10	(M+Na)+
412.1126	1	2002.65	C17H22NaO10	(M+Na)+
425.0838	1	40159.59	C17H22KO10	(M+K)+
426.0876	1	7419.61	C17H22KO10	(M+K)+
427.0842	1	4313.16	C17H22KO10	(M+K)+
428.09	1	774.52	C17H22KO10	(M+K)+

--- End Of Report ---

Mass spectrum of 3i

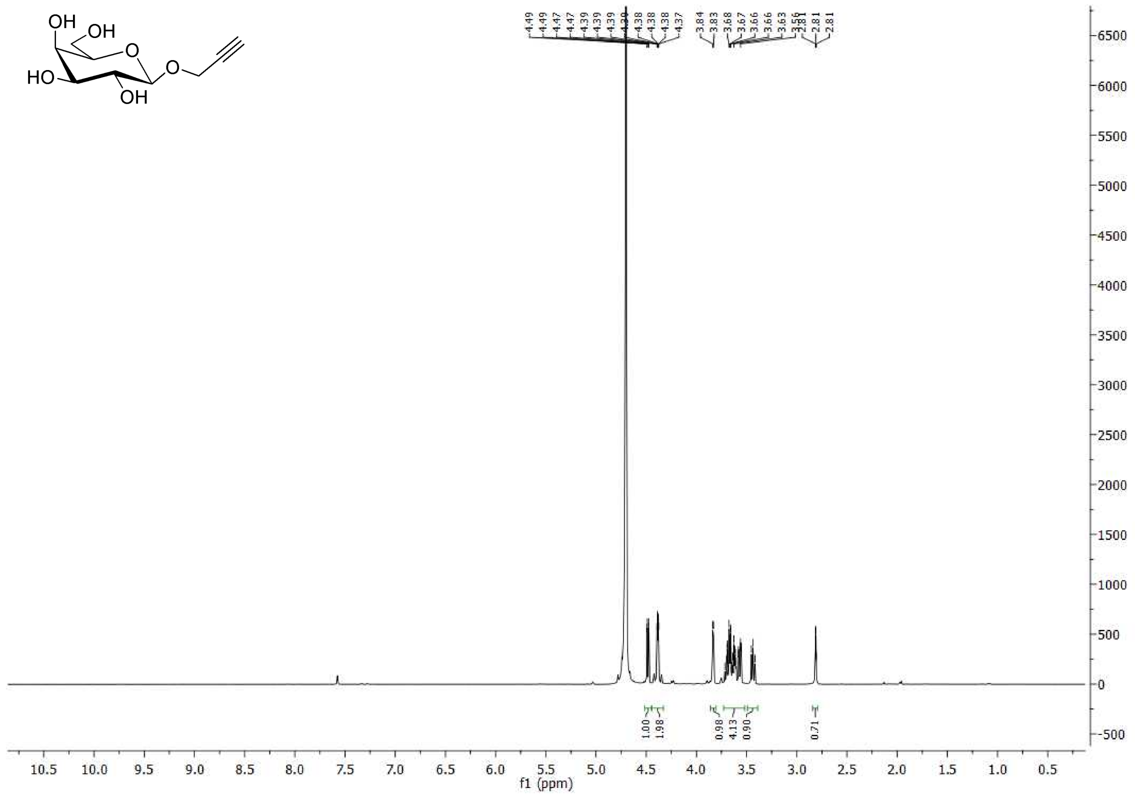
OH
OH
O
O
HO
OH
4.49
4.49
4.47
4.47
4.39
4.39
4.39
4.38
4.38
4.38
4.37
3.84
3.83
3.68
3.67
3.66
3.66
3.63
2.81
2.81
1.00
1.98
0.98
4.13
0.90
0.71
f1 (ppm)

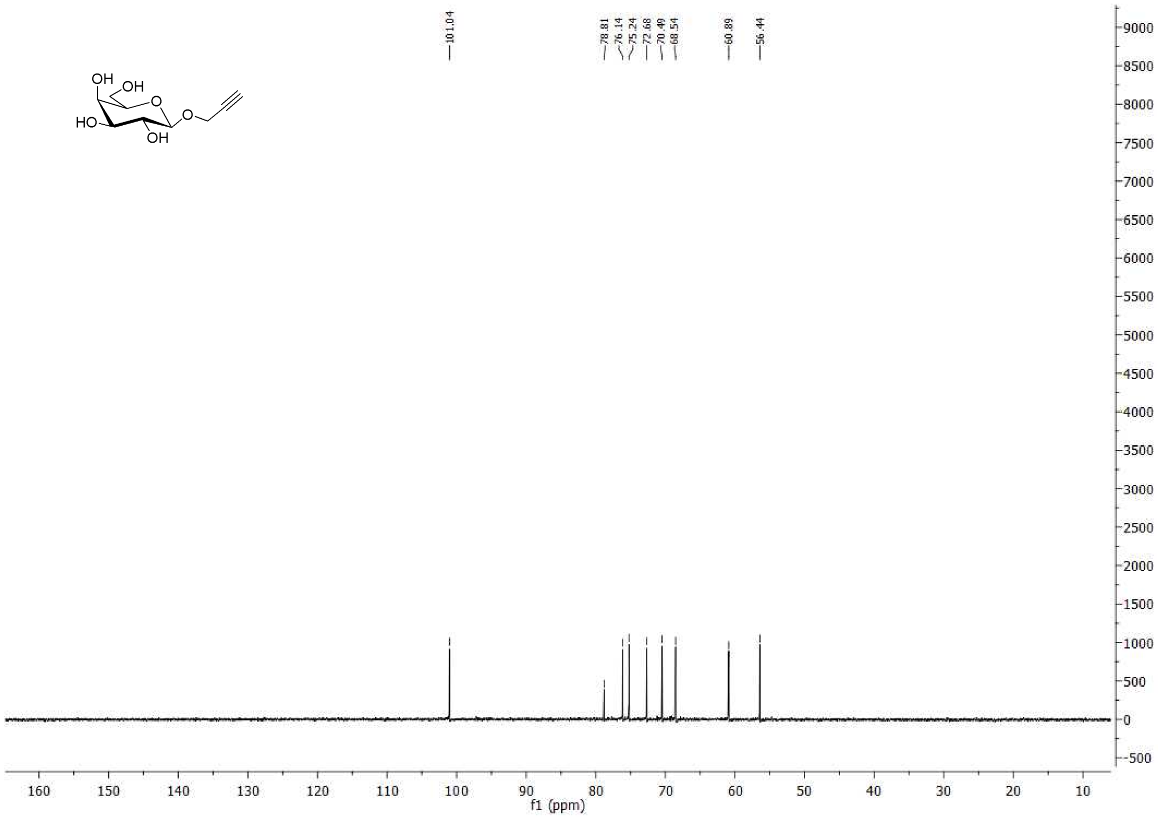
OH
OH
O
O
HO
OH
101.04
78.81
76.14
75.24
72.68
70.49
68.54
60.89
56.44
f1 (ppm)

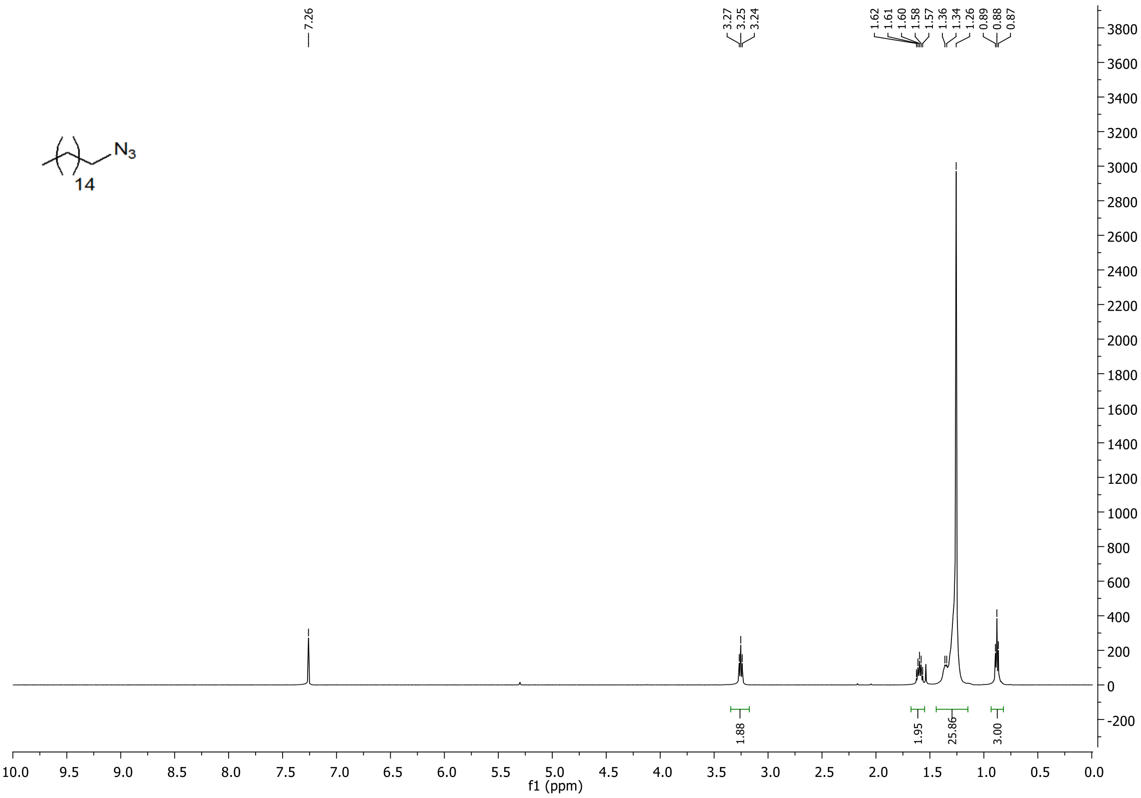

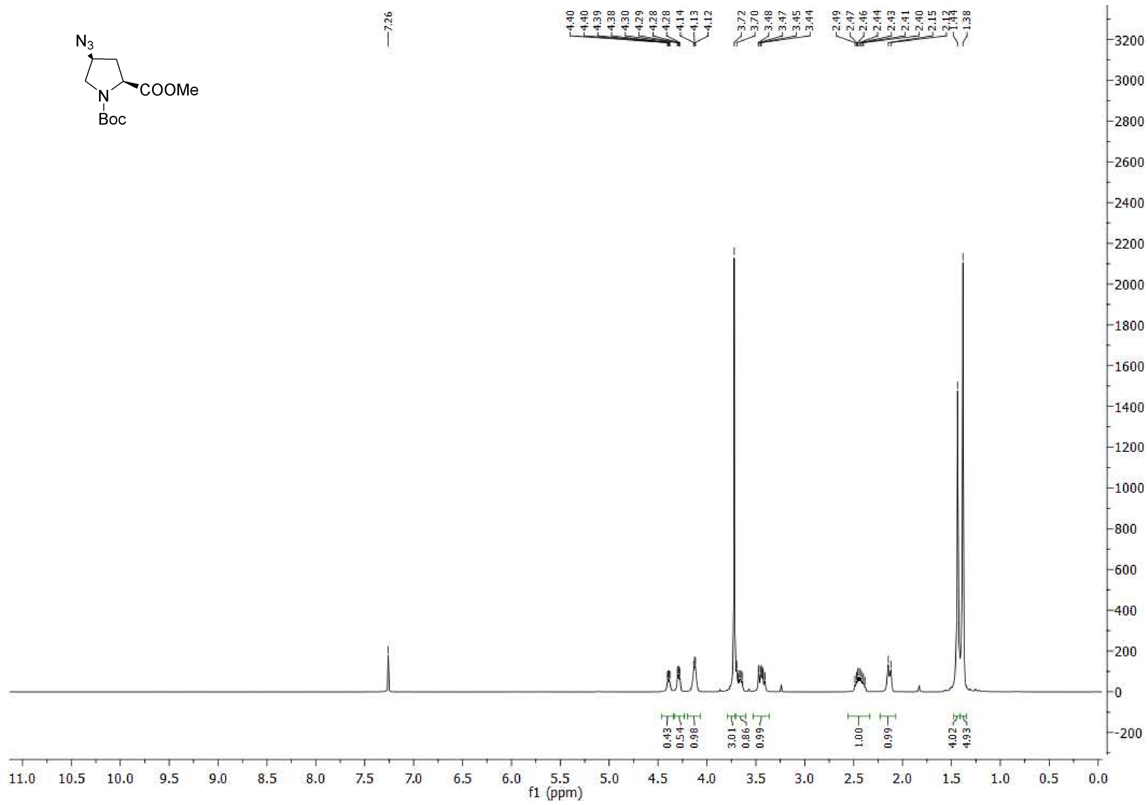

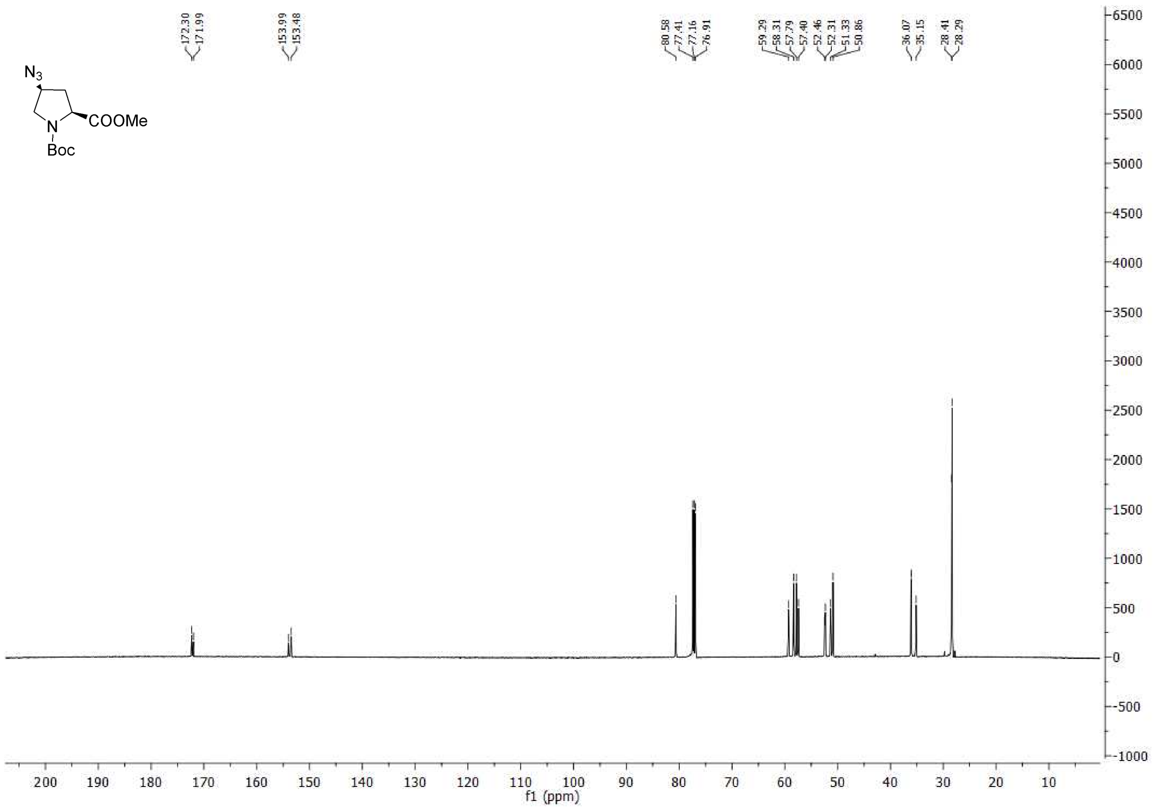
N3
N
COOMe
Boc
172.30
171.99
153.99
153.48
80.58
77.41
77.16
76.91
59.29
58.31
57.79
57.40
52.46
52.31
51.33
50.86
36.07
35.15
28.41
28.29
f1 (ppm)

Qualitative Compound Report

**Data File**	rr-1-161.d	**Sample Name**	rr-1-161
**Sample Type**	Sample	**Position**	Vial 1
**Instrument Name**	QTOF	**User Name**	QTOF-PC\admin
**Acq Method**	ACgroup_new.m	**Acquired Time**	2020-07-28 12:27:05
**IRM Calibration Status**	Success	**DA Method**	Default.m
**Comment**			

Acquisition SW Version 6200 series TOF/6500 series Q-TOF B.05.00 (B5042.2)

Compound Table

Compound Label	RT	Mass	Abund	Formula	Tgt Mass	Diff (ppm)	MFG Formula	DB Formula
Cpd 1: C11 H18 N4 O4	1.151	270.1315	7319	C11 H18 N4 O4	270.1328	-4.85	C11 H18 N4 O4	C11 H18 N4 O4

Compound Label	*m/z*	RT	Algorithm	Mass
Cpd 1: C11 H18 N4 O4	309.0951	1.151	Find By Formula	270.1315

MS Zoomed Spectrum

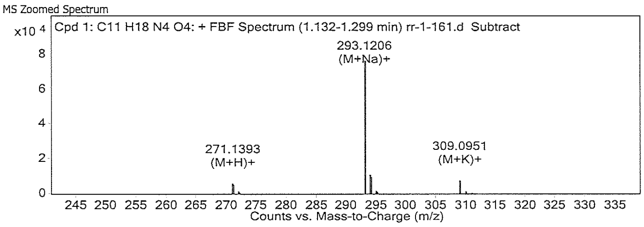

MS Spectrum Peak List

m/z	z	Abund	Formula	Ion
271.1393	1	5508.7	C11H19N4O4	(M+H)+
272.1421	1	776.07	C11H19N4O4	(M+H)+
293.1206	1	76404.57	C11H18N4NaO4	(M+Na)+
294.1232	1	10196.9	C11H18N4NaO4	(M+Na)+
295.1263	1	1342.75	C11H18N4NaO4	(M+Na)+
309.0951	1	7318.73	C11H18KN4O4	(M+K)+
310.0974	1	1058.4	C11H18KN4O4	(M+K)+
311.0944	1	732.4	C11H18KN4O4	(M+K)+
312.0956	1	75.21	C11H18KN4O4	(M+K)+

--- End Of Report ---

Mass spectrum of 2c

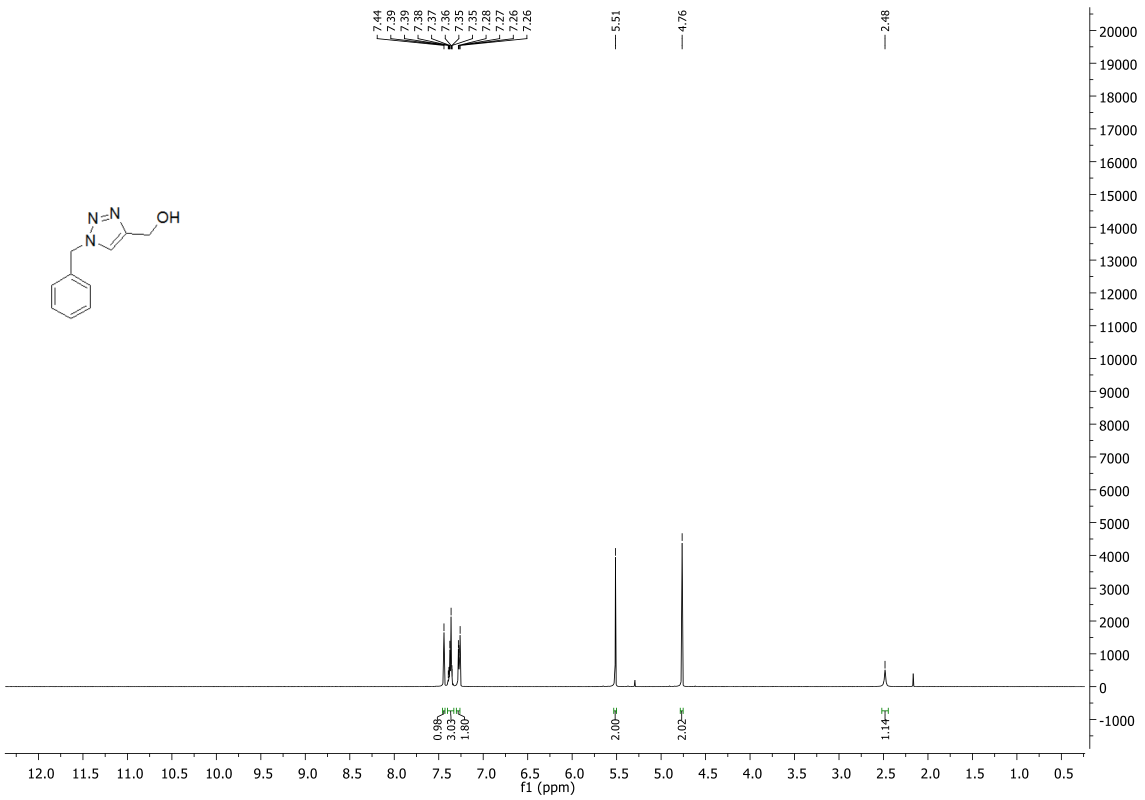
7.44
7.39
7.39
7.38
7.37
7.36
7.35
7.35
7.28
7.27
7.26
7.26
5.51
4.76
2.48
N=N
N
OH
0.98
3.03
1.80
2.00
2.02
1.14
f1 (ppm)

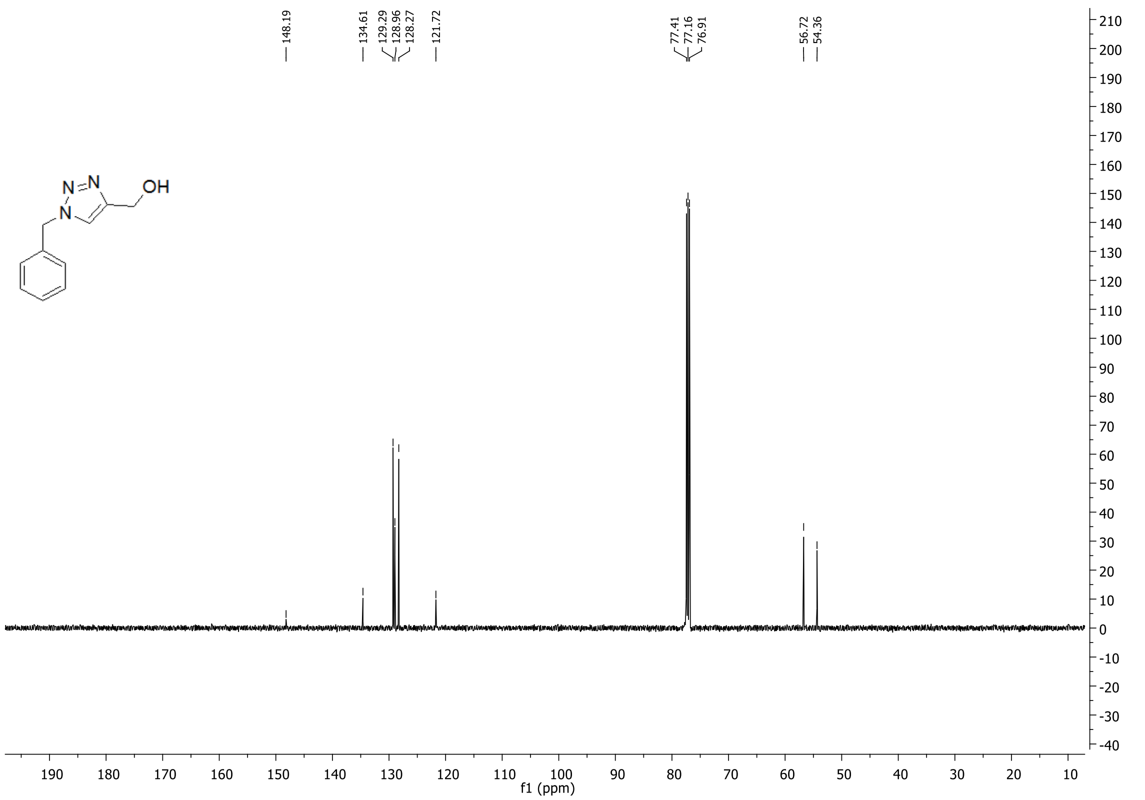
148.19
134.61
129.29
128.96
128.27
121.72
77.41
77.16
76.91
56.72
54.36
N=N
N
OH
f1 (ppm)

Qualitative Compound Report

**Data File**	rr-1-91-first reaction-after column.d	**Sample Name**	rr-1-91-first reaction-after column
**Sample Type**	Sample	**Position**	vial 1
**Instrument Name**	QTOF	**User Name**	QTOF-PC\admin
**Acq Method**	ACgroup_new.m	**Acquired Time**	2018-12-12 17:13:24
**IRM Calibration Status**	Success	**DA Method**	szfg123.m
**Comment**			

**Acquisition SW Version**	6200 series TOF/6500 series Q-TOF B.05.00 (B5042.2)

Compound Table

Compound Label	RT	Mass	Abund	Formula	Tgt Mass	Diff (ppm)	MFG Formula	DB Formula
Cpd 1: C10 H11 N3 O	0.532	189.0904	300076	C10 H11 N3 O	189.0902	1.12	C10 H11 N3 O	C10 H11 N3 O

Compound Label	*m/z*	RT	Algorithm	Mass
Cpd 1: C10 H11 N3 O	190.0977	0.532	Find By Formula	189.0904

MS Zoomed Spectrum

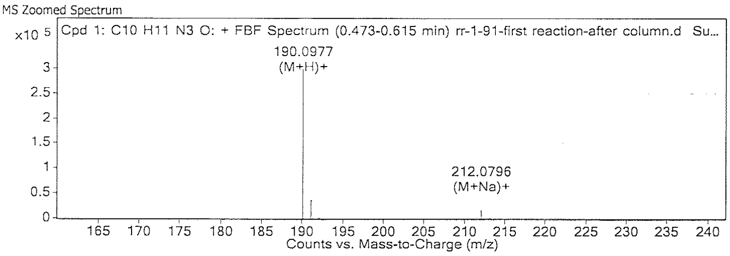

MS Spectrum Peak List

m/z	z	Abund	Formula	Ion
190.0977	1	300076.13	C10H12N3O	(M+H)+
191.1005	1	35483.06	C10H12N3O	(M+H)+
192.1031	1	2806.79	C10H12N3O	(M+H)+
212.0796	1	15348.46	C10H11N3NaO	(M+Na)+
213.0823	1	1923.97	C10H11N3NaO	(M+Na)+
214.0849	1	180.49	C10H11N3NaO	(M+Na)+

--- End Of Report ---

Mass spectrum of 4a

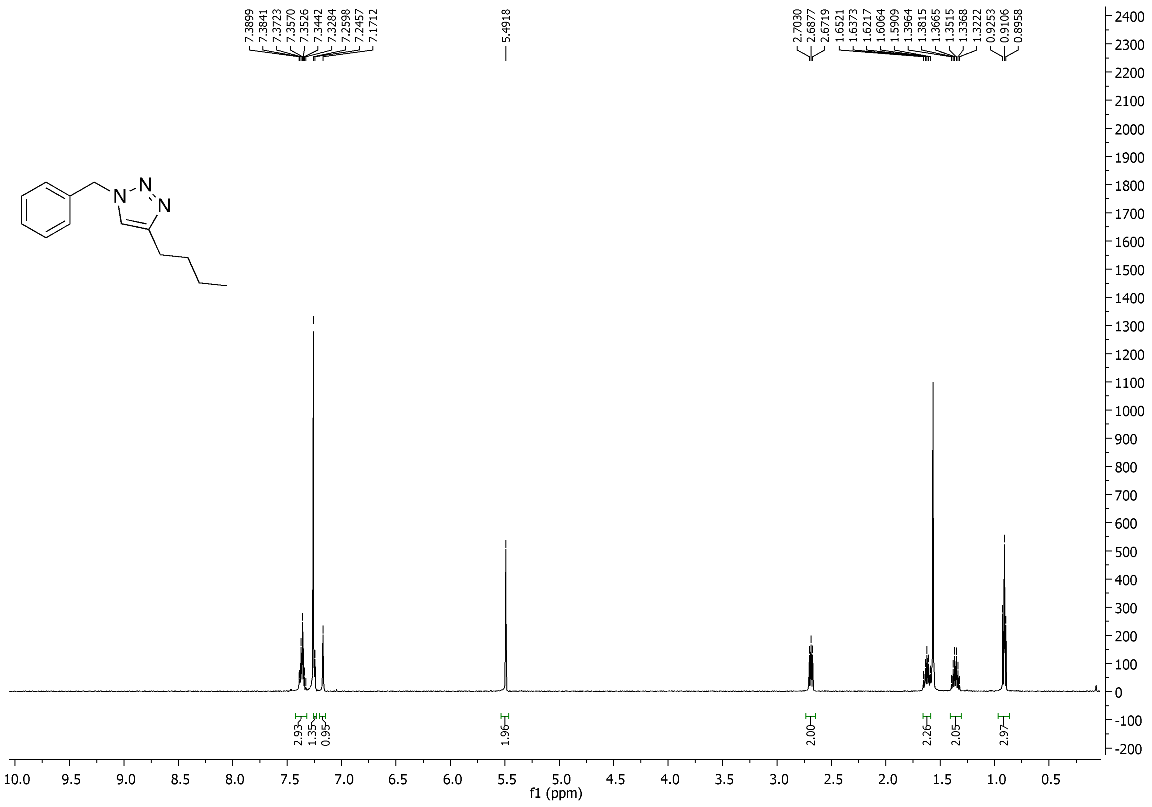

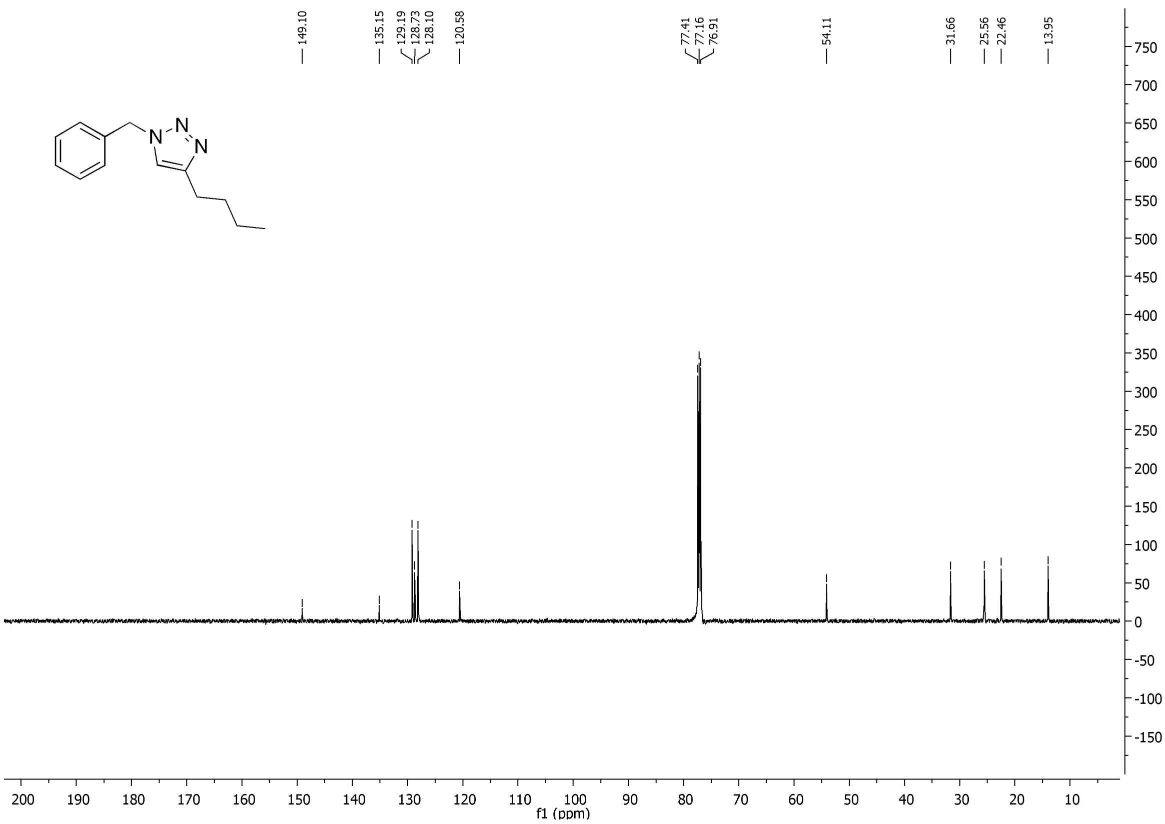

Qualitative Compound Report

**Data File**	rr-97.d	**Sample Name**	rr-97
**Sample Type**	Sample	**Position**	Vial 1
**Instrument Name**	QTOF	**User Name**	QTOF-PC\admin
**Acq Method**	ACgroup_new.m	**Acquired Time**	2019-02-28 12:11:25
**IRM Calibration Status**	Success	**DA Method**	szfg123.m
**Comment**			

Acquisition SW Version 6200 series TOF/6500 series Q-TOF B.05.00 (B5042.2)

Compound Table

Compound Label	RT	Mass	Abund	Formula	Tgt Mass	Diff (ppm)	MFG Formula	DB Formula
Cpd 1: C13 H17 N3	0.783	215.1422	1002971	C13 H17 N3	215.1422	-0.36	C13 H17 N3	C13 H17 N3

Compound Label	*m/z*	RT	Algorithm	Mass
Cpd 1: C13 H17 N3	216.1494	0.783	Find By Formula	215.1422

MS Zoomed Spectrum

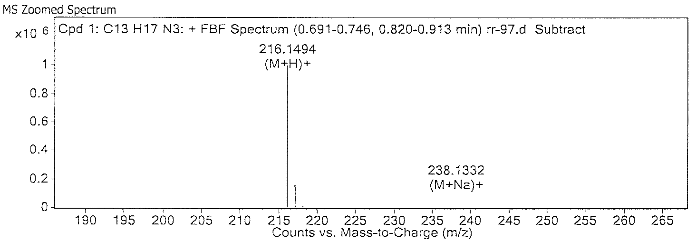

MS Spectrum Peak List

m/z	z	Abund	Formula	Ion
216.1494	1	1002971.38	C13H18N3	(M+H)+
217.1527	1	152635.83	C13H18N3	(M+H)+
218.1558	1	10854.13	C13H18N3	(M+H)+
219.1557	1	548.41	C13H18N3	(M+H)+
238.1332	1	1645.26	C13H17N3Na	(M+Na)+
239.1339	1	258.12	C13H17N3Na	(M+Na)+

--- End Of Report ---

Mass spectrum of 4b

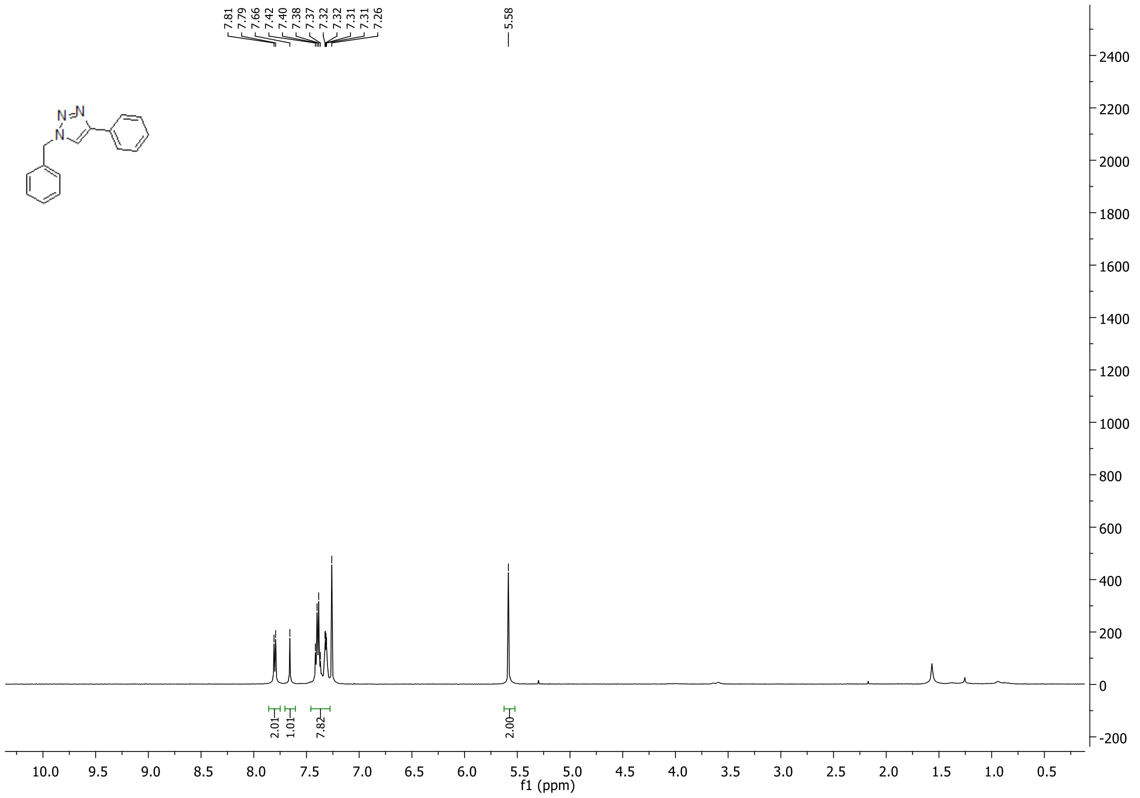
7.81
7.79
7.66
7.42
7.40
7.38
7.37
7.32
7.32
7.31
7.31
7.26
5.58
2.01
1.01
7.82
2.00
f1 (ppm)

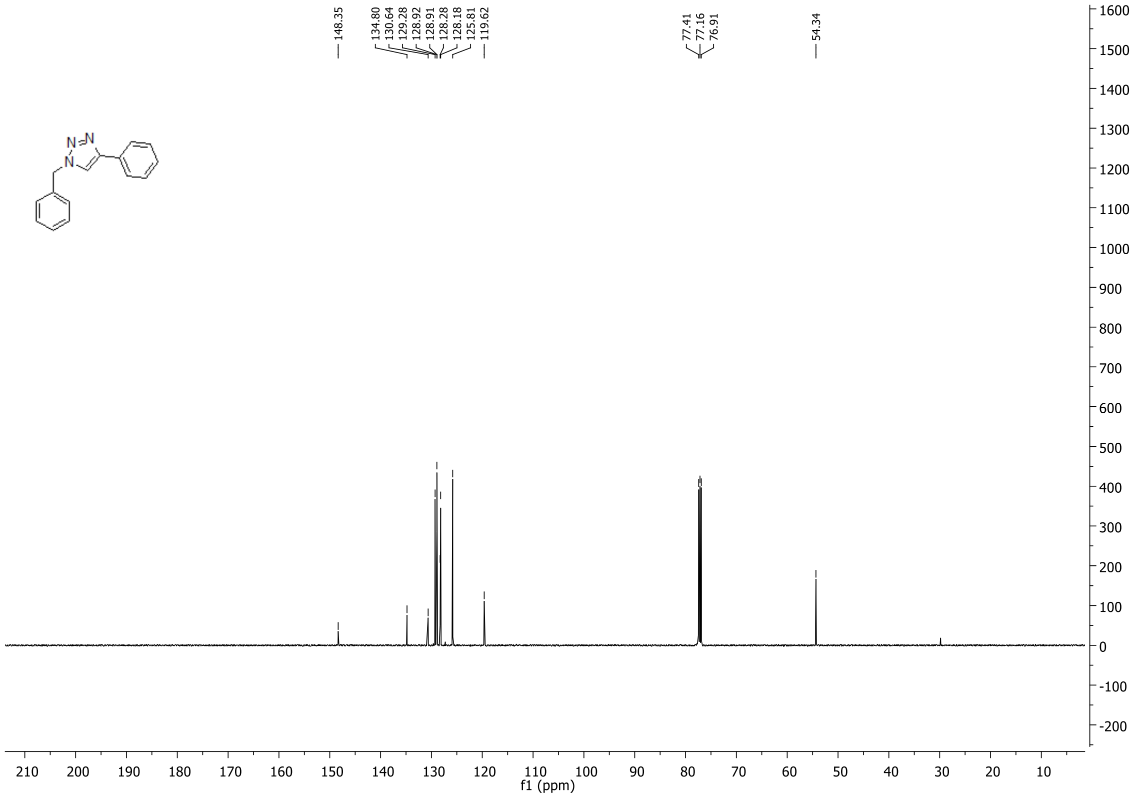
148.35
134.80
130.64
129.28
128.92
128.91
128.28
128.18
125.81
119.62
77.41
77.16
76.91
54.34
N
N
N
f1 (ppm)

Qualitative Compound Report

**Data File**	rr-100.d	**Sample Name**	rr-100
**Sample Type**	Sample	**Position**	Vial 1
**Instrument Name**	QTOF	**User Name**	QTOF-PC\admin
**Acq Method**	ACgroup_new.m	**Acquired Time**	2019-02-28 15:56:25
**IRM Calibration Status**	Success	**DA Method**	szfg123.m
**Comment**			

**Acquisition SW Version**	6200 series TOF/6500 series Q-TOF B.05.00 (B5042.2)

Compound Table

Compound Label	RT	Mass	Abund	Formula	Tgt Mass	Diff (ppm)	MFG Formula	DB Formula
Cpd 1: C15 H13 N3	0.782	235.1108	1261175	C15 H13 N3	235.1109	-0.69	C15 H13 N3	C15 H13 N3

Compound Label	*m/z*	RT	Algorithm	Mass
Cpd 1: C15 H13 N3	236.118	0.782	Find By Formula	235.1108

MS Zoomed Spectrum

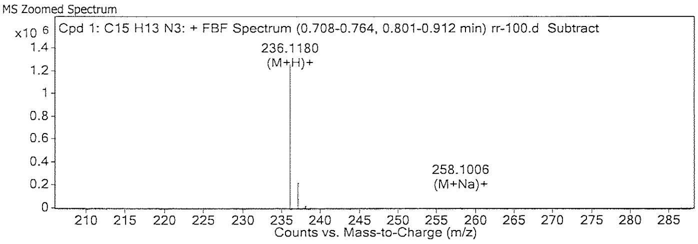

MS Spectrum Peak List

m/z	z	Abund	Formula	Ion
236.118	1	1261174.5	C15H14N3	(M+H)+
237.1215	1	216110.14	C15H14N3	(M+H)+
238.1244	1	17251.7	C15H14N3	(M+H)+
239.1322	1	988.26	C15H14N3	(M+H)+
258.1006	1	4008.32	C15H13N3Na	(M+Na)+
259.1035	1	897.76	C15H13N3Na	(M+Na)+

--- End Of Report ---

Mass spectrum of 4c

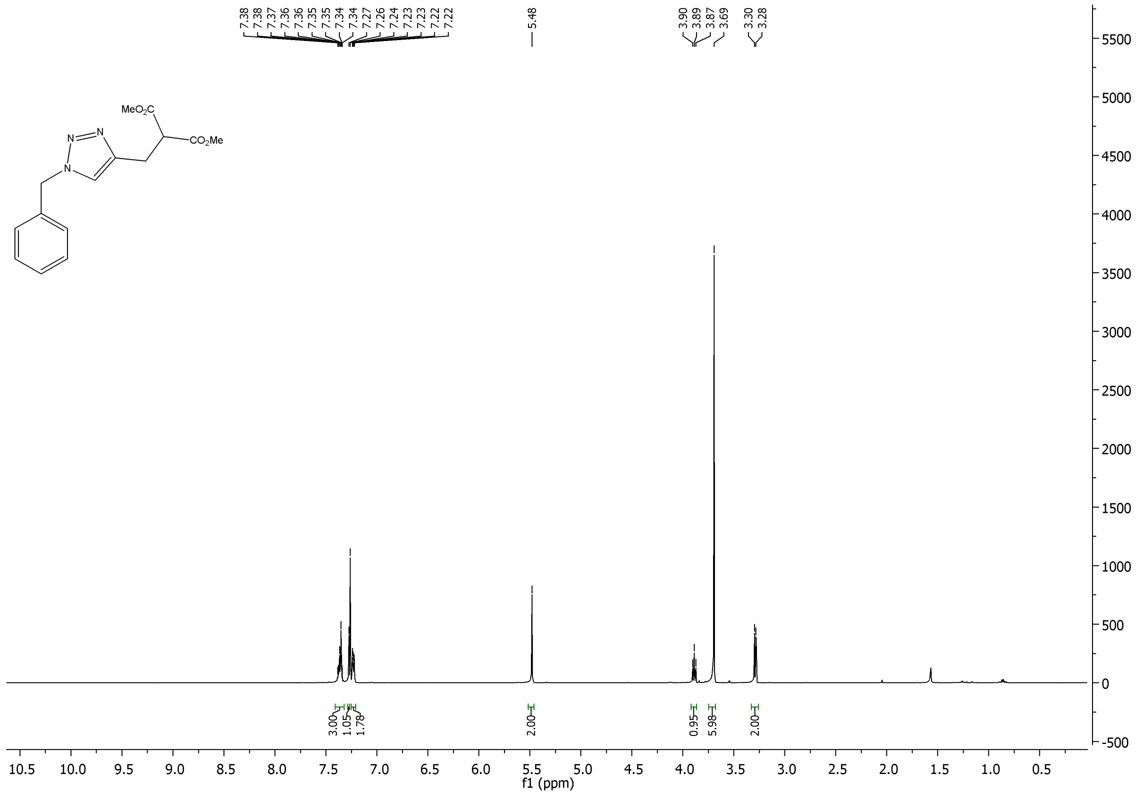
MeO2C
N
N
N
CO2Me
7.38
7.38
7.37
7.36
7.36
7.35
7.35
7.34
7.34
7.27
7.26
7.24
7.23
7.23
7.22
7.22
5.48
3.90
3.89
3.87
3.69
3.30
3.28
3.00
1.05
1.78
2.00
0.95
5.98
2.00
f1 (ppm)

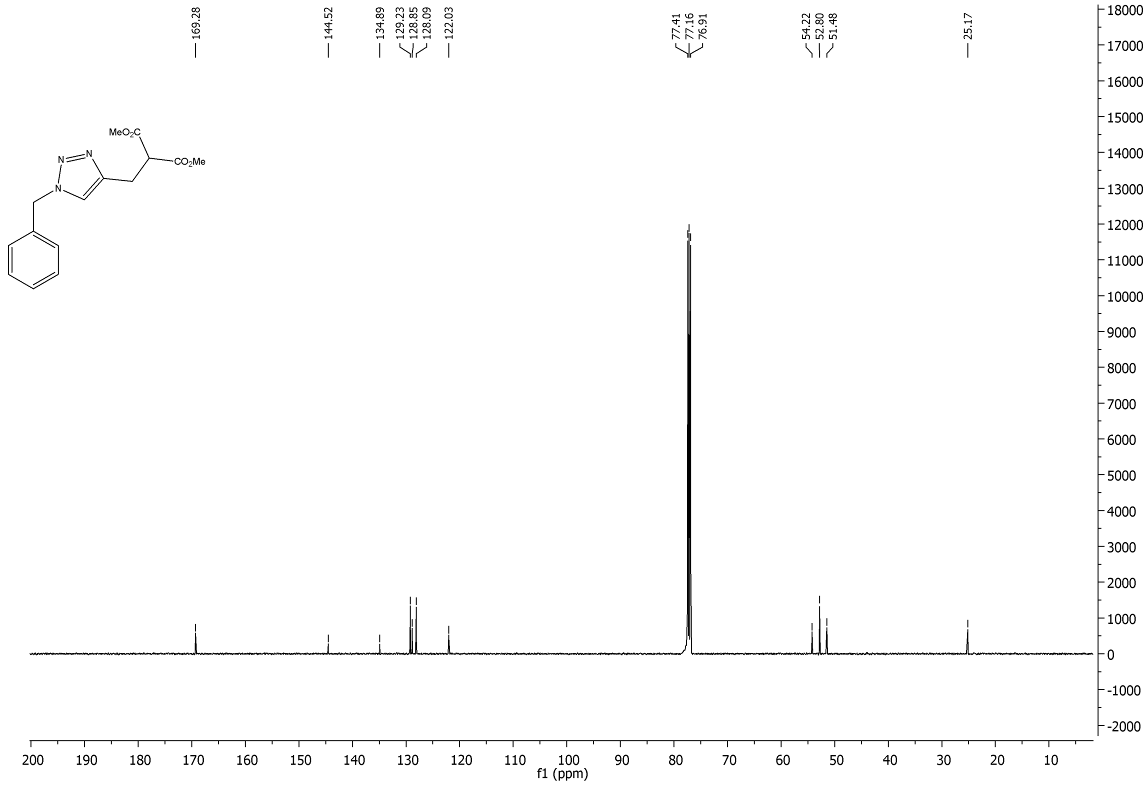
MeO2C
N
N
N
CO2Me
169.28
144.52
134.89
129.23
128.85
128.09
122.03
77.41
77.16
76.91
54.22
52.80
51.48
25.17
f1 (ppm)

Qualitative Compound Report

**Data File**	rr-102 (pos.mode).d	**Sample Name**	rr-102 (pos.mode)
**Sample Type**	Sample	**Position**	Vial 1
**Instrument Name**	QTOF	**User Name**	QTOF-PC\admin
**Acq Method**	ACgroup_new.m	**Acquired Time**	2019-07-10 13:43:52
**IRM Calibration Status**	Success	**DA Method**	szfg123.m
**Comment**			

Acquisition SW 6200 series TOF/6500 series
Version Q-TOF B.05.00 (B5042.2)

Compound Table

Compound Label	RT	Mass	Abund	Formula	Tgt Mass	Diff (ppm)	MFG Formula	DB Formula
Cpd 1: C15 H17 N3 O4	0.548	303.1235	18332	C15 H17 N3 O4	303.1219	5.35	C15 H17 N3 O4	C15 H17 N3 O4

Compound Label	*m/z*	RT	Algorithm	Mass
Cpd 1: C15 H17 N3 O4	326.1115	0.548	Find By Formula	303.1235

MS Zoomed Spectrum

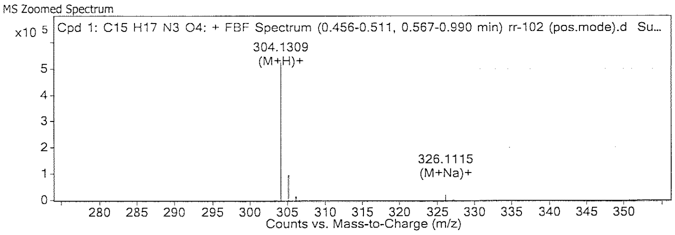

MS Spectrum Peak List

m/z	z	Abund	Formula	Ion
304.1309	1	524963.38	C15H18N3O4	(M+H)+
305.1337	1	93589.76	C15H18N3O4	(M+H)+
306.1351	1	12270.55	C15H18N3O4	(M+H)+
307.1366	1	1171.83	C15H18N3O4	(M+H)+
326.1115	1	18332.31	C15H17N3NaO4	(M+Na)+
327.1142	1	3314.85	C15H17N3NaO4	(M+Na)+
328.1154	1	507.78	C15H17N3NaO4	(M+Na)+

--- End Of Report ---

Mass spectrum of 4d

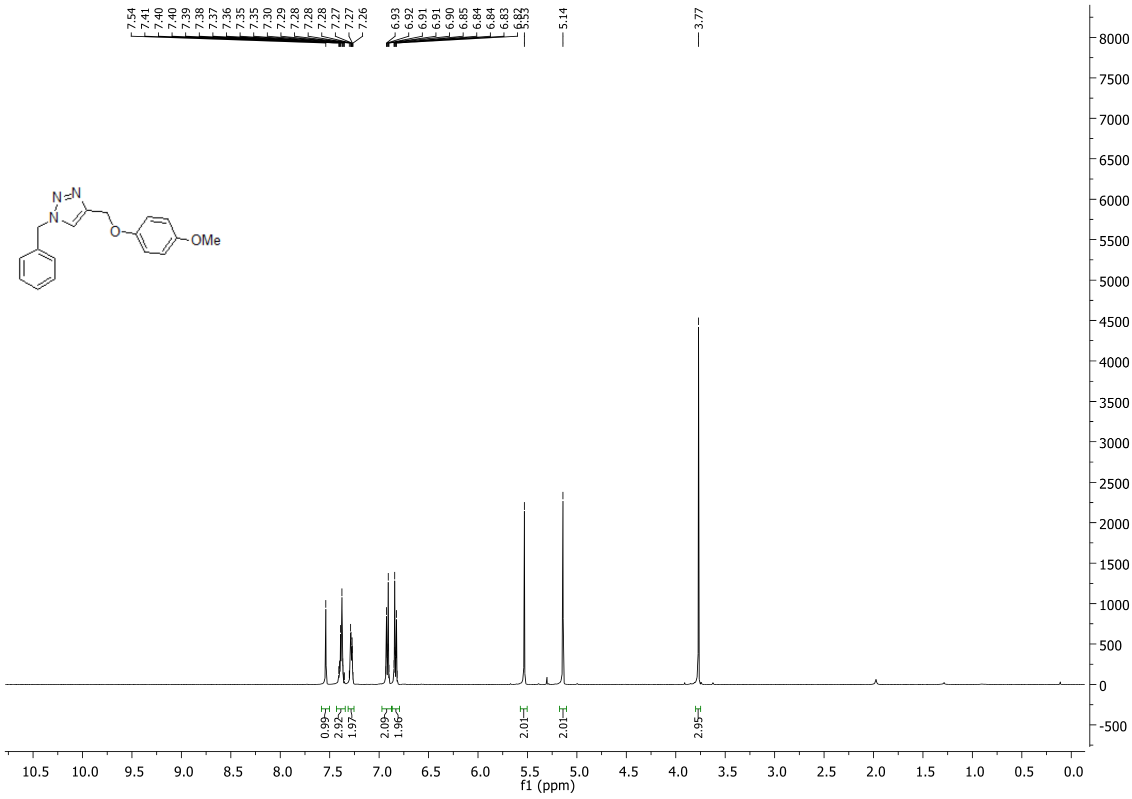
7.54
7.41
7.40
7.39
7.38
7.37
7.36
7.35
7.35
7.30
7.29
7.28
7.28
7.28
7.27
7.27
7.26
6.93
6.92
6.91
6.91
6.90
6.85
6.84
6.84
6.83
6.82
5.53
5.14
3.77
N=N
N
O
OMe
0.99
2.92
1.97
2.09
1.96
2.01
2.01
2.95
f1 (ppm)

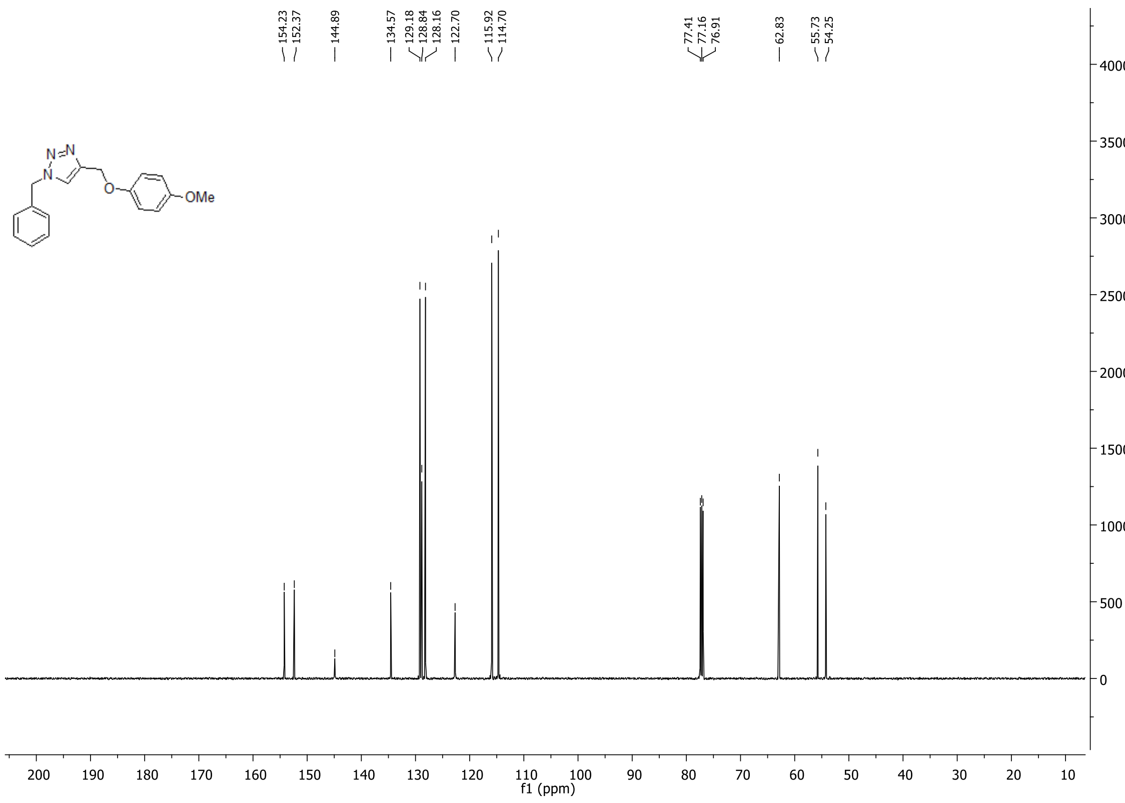
154.23
152.37
144.89
134.57
129.18
128.84
128.16
122.70
115.92
114.70
77.41
77.16
76.91
62.83
55.73
54.25
N=N
N
O
OMe
f1 (ppm)

Qualitative Compound Report

**Data File**	rr-106.d	**Sample Name**	rr-106
**Sample Type**	Sample	**Position**	Vial 1
**Instrument Name**	QTOF	**User Name**	QTOF-PC\admin
**Acq Method**	ACgroup_new.m	**Acquired Time**	2019-02-28 15:42:25
**IRM Calibration Status**	Success	**DA Method**	szfg123.m
**Comment**			

**Acquisition SW Version**	6200 series TOF/6500 series Q-TOF B.05.00 (B5042.2)

Compound Table

Compound Label	RT	Mass	Abund	Formula	Tgt Mass	Diff (ppm)	MFG Formula	DB Formula
Cpd 1: C17 H17 N3 O2	0.736	295.132	978645	C17 H17 N3 O2	295.1321	-0.35	C17 H17 N3 O2	C17 H17 N3 O2

Compound Label	*m/z*	RT	Algorithm	Mass
Cpd 1: C17 H17 N3 O2	296.1391	0.736	Find By Formula	295.132

MS Zoomed Spectrum

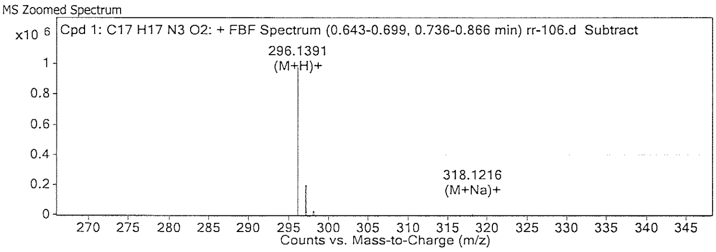

MS Spectrum Peak List

m/z	z	Abund	Formula	Ion
296.1391	1	978644.81	C17H18N3O2	(M+H)+
297.1428	1	189740.7	C17H18N3O2	(M+H)+
298.1453	1	21229.53	C17H18N3O2	(M+H)+
299.1521	1	2524.63	C17H18N3O2	(M+H)+
318.1216	1	7998.26	C17H17N3NaO2	(M+Na)+
319.1112	1	1149.1	C17H17N3NaO2	(M+Na)+
320.1087	1	114.42	C17H17N3NaO2	(M+Na)+

--- End Of Report ---

Mass spectrum of 4e

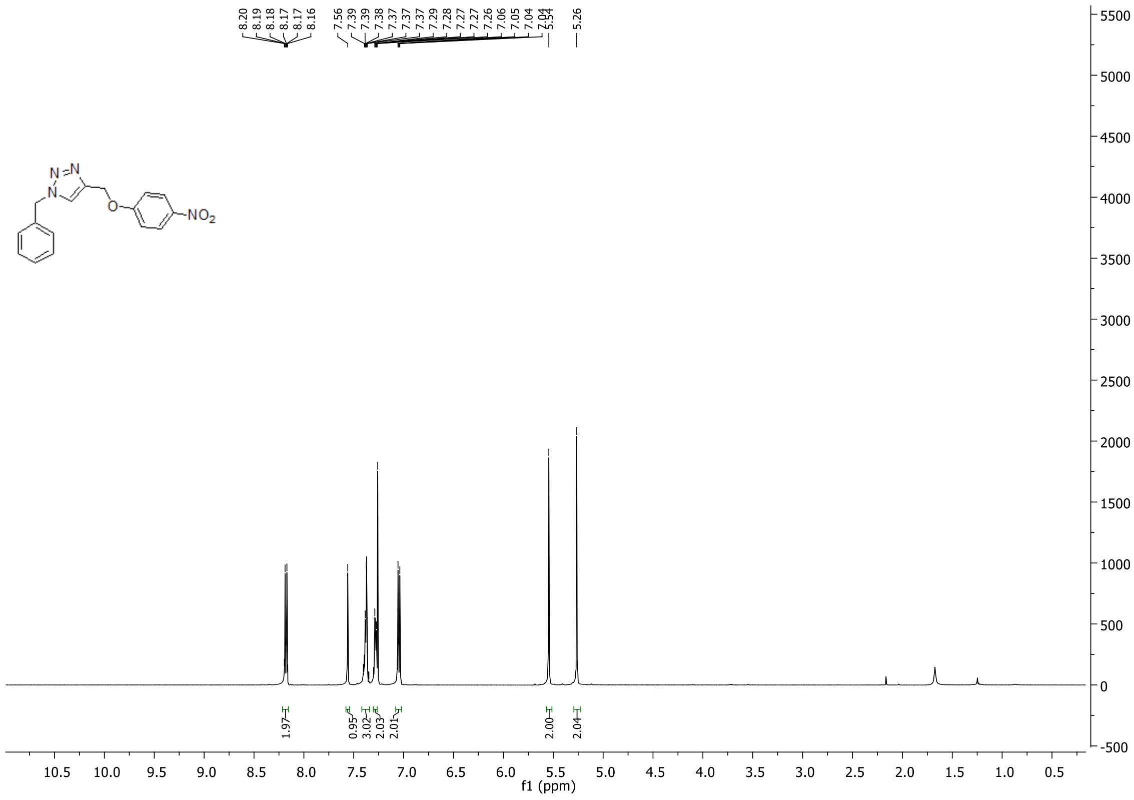
8.20
8.19
8.18
8.17
8.17
8.16
7.56
7.39
7.39
7.38
7.37
7.37
7.37
7.29
7.28
7.27
7.27
7.26
7.06
7.05
7.04
7.04
5.54
5.26
NO2
1.97
0.95
3.02
2.03
2.01
2.00
2.04
f1 (ppm)

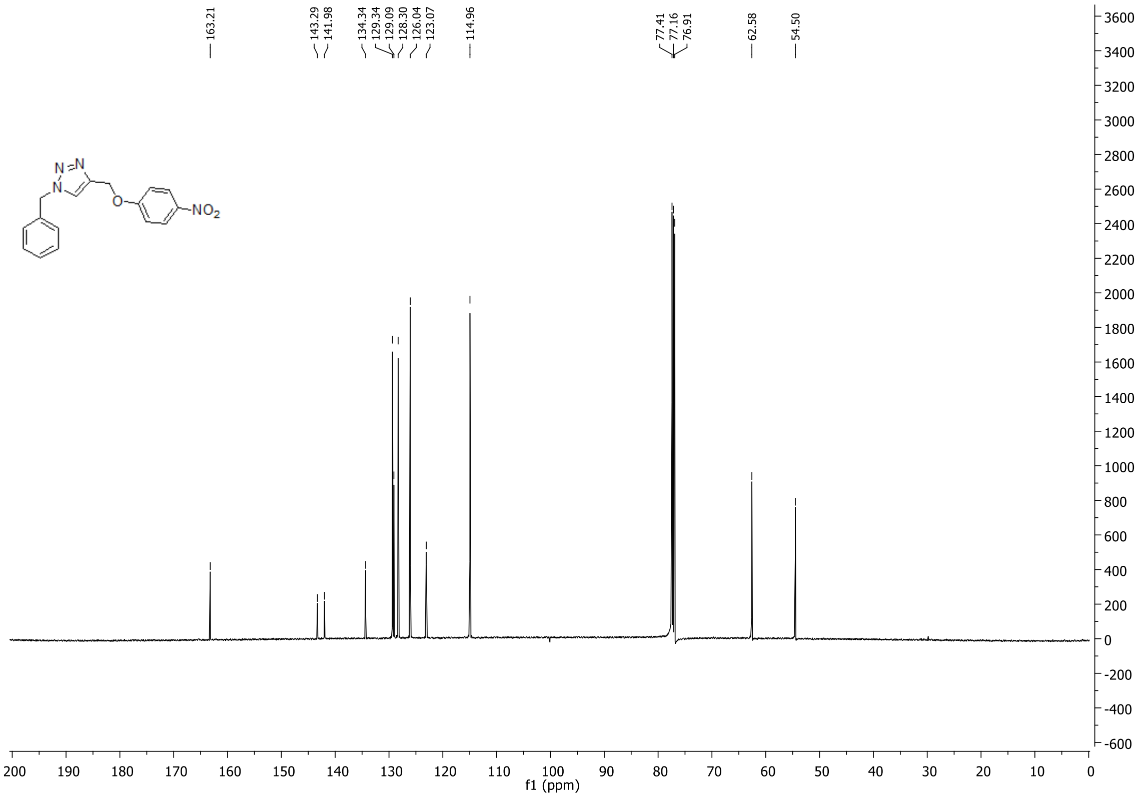
163.21
143.29
141.98
134.34
129.34
129.09
128.30
126.04
123.07
114.96
77.41
77.16
76.91
62.58
54.50
NO2
f1 (ppm)

Qualitative Compound Report

**Data File**	rr-101.d	**Sample Name**	rr-101
**Sample Type**	Sample	**Position**	Vial 1
**Instrument Name**	QTOF	**User Name**	QTOF-PC\admin
**Acq Method**	ACgroup_new.m	**Acquired Time**	2019-02-28 15:03:00
**IRM Calibration Status**	Success	**DA Method**	szfg123.m
**Comment**			

**Acquisition SW Version**	6200 series TOF/6500 series Q-TOF B.05.00 (B5042.2)

Compound Table

Compound Label	RT	Mass	Abund	Formula	Tgt Mass	Diff (ppm)	MFG Formula	DB Formula
Cpd 1: C16 H14 N4 O3	0.753	310.1065	667181	C16 H14 N4 O3	310.1066	-0.25	C16 H14 N4 O3	C16 H14 N4 O3

Compound Label	*m/z*	RT	Algorithm	Mass
Cpd 1: C16 H14 N4 O3	311.1137	0.753	Find By Formula	310.1065

MS Zoomed Spectrum

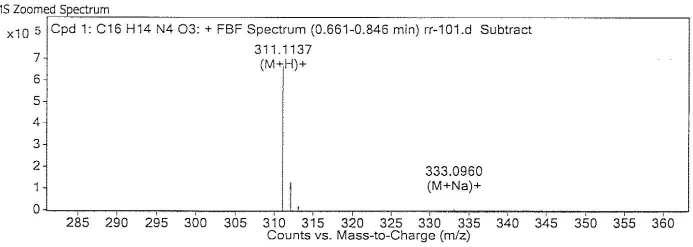

MS Spectrum Peak List

m/z	z	Abund	Formula	Ion
311.1137	1	667180.88	C16H15N4O3	(M+H)+
312.1171	1	123171.2	C16H15N4O3	(M+H)+
313.1195	1	15200.45	C16H15N4O3	(M+H)+
314.1226	1	1426.77	C16H15N4O3	(M+H)+
333.096	1	6429.59	C16H14N4NaO3	(M+Na)+
334.0992	1	1326.46	C16H14N4NaO3	(M+Na)+
335.1098	1	118.11	C16H14N4NaO3	(M+Na)+

--- End Of Report ---

Mass spectrum of 4f

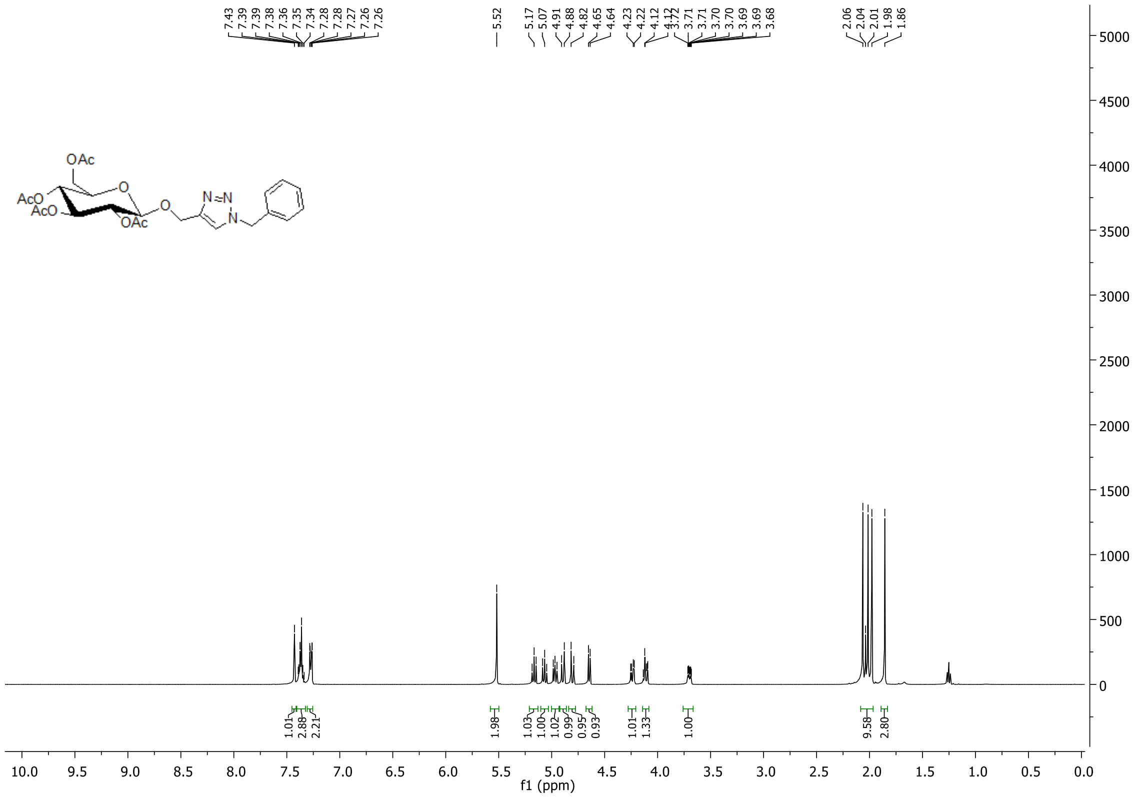
7.43
7.39
7.39
7.38
7.36
7.35
7.34
7.28
7.28
7.27
7.26
7.26
5.52
5.17
5.07
4.91
4.88
4.82
4.65
4.64
4.23
4.22
4.12
4.12
3.72
3.71
3.71
3.70
3.70
3.69
3.69
3.68
2.06
2.04
2.01
1.98
1.86
OAc
AcO
AcO
O
OAc
O
N=N
N
1.01
2.88
2.21
1.98
1.03
1.00
1.02
0.99
0.95
0.93
1.01
1.33
1.00
9.58
2.80
f1 (ppm)

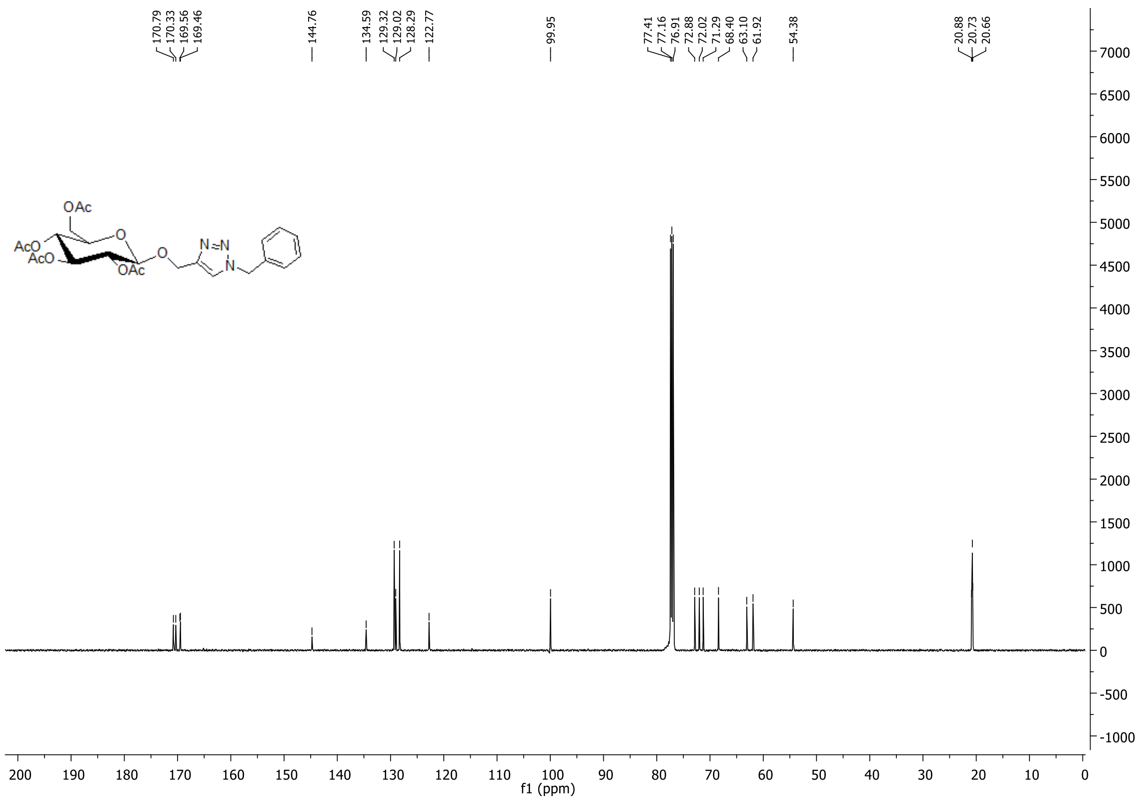
170.79
170.33
169.56
169.46
144.76
134.59
129.32
129.02
128.29
122.77
99.95
77.41
77.16
76.91
72.88
72.02
71.29
68.40
63.10
61.92
54.38
20.88
20.73
20.66
OAc
AcO
AcO
O
OAc
O
N=N
N
f1 (ppm)

Qualitative Compound Report

**Data File**	rr-108 (pos.mode).d	**Sample Name**	rr-108 (pos.mode)
**Sample Type**	Sample	**Position**	Vial 1
**Instrument Name**	QTOF	**User Name**	QTOF-PC\admin
**Acq Method**	ACgroup_new.m	**Acquired Time**	2019-07-10 13:54:19
**IRM Calibration Status**	Success	**DA Method**	szfg123.m
**Comment**			

**Acquisition SW Version**	6200 series TOF/6500 series Q-TOF B.05.00 (B5042.2)

Compound Table

Compound Label	RT	Mass	Abund	Formula	Tgt Mass	Diff (ppm)	MFG Formula	DB Formula
Cpd 1: C24 H29 N3 O10	0.549	519.1875	16856	C24 H29 N3 O10	519.1853	4.3	C24 H29 N3 O10	C24 H29 N3 O10

Compound Label	*m/z*	RT	Algorithm	Mass
Cpd 1: C24 H29 N3 O10	542.1751	0.549	Find By Formula	519.1875

MS Zoomed Spectrum

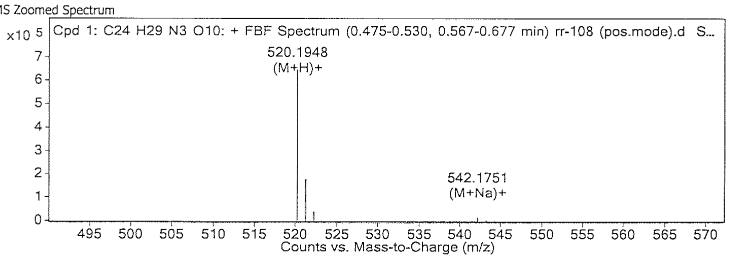

MS Spectrum Peak List

m/z	z	Abund	Formula	Ion
520.1948	1	649171.56	C24H30N3O10	(M+H)+
521.1985	1	178866.86	C24H30N3O10	(M+H)+
522.1994	1	37397.57	C24H30N3O10	(M+H)+
523.2018	1	5166.88	C24H30N3O10	(M+H)+
524.1986	1	707.57	C24H30N3O10	(M+H)+
542.1751	1	16855.91	C24H29N3NaO10	(M+Na)+
543.1786	1	4851.36	C24H29N3NaO10	(M+Na)+
544.1791	1	1147.26	C24H29N3NaO10	(M+Na)+
545.1885	1	132.7	C24H29N3NaO10	(M+Na)+

--- End Of Report ---

Mass spectrum of 4g

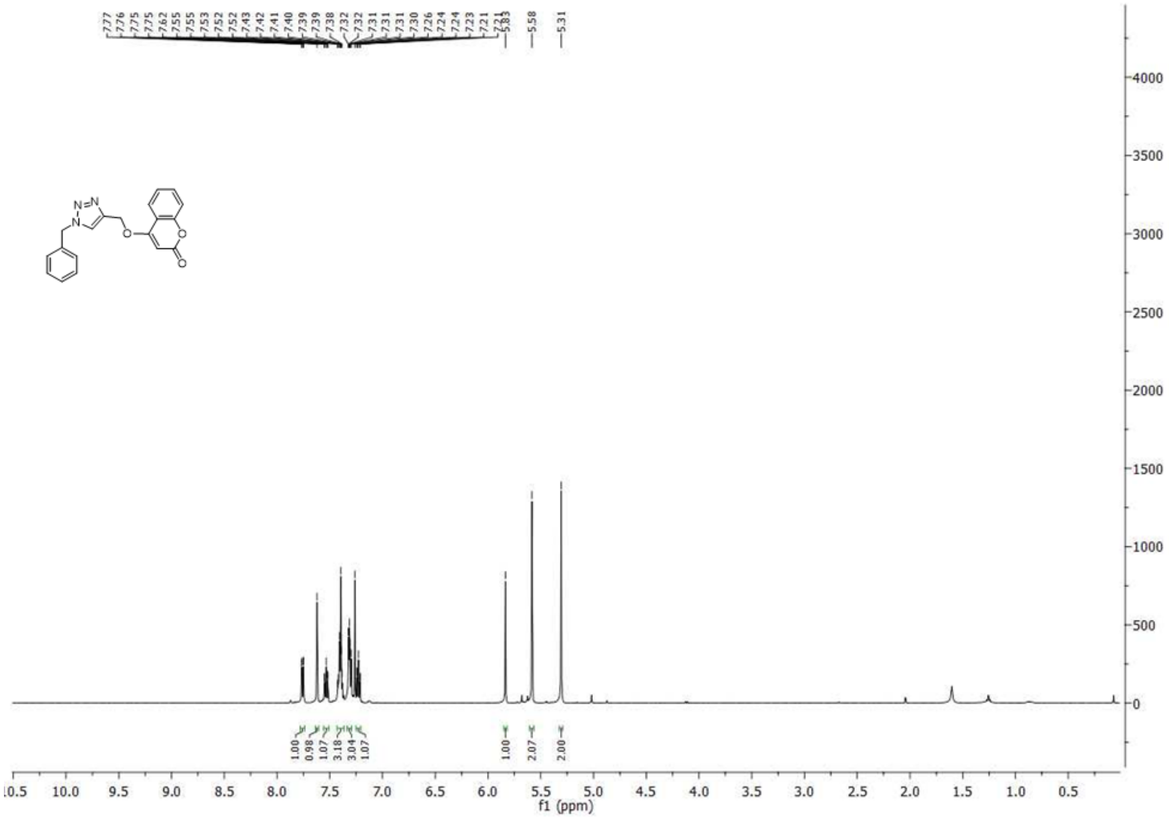
7.77
7.76
7.75
7.75
7.62
7.55
7.55
7.53
7.52
7.52
7.43
7.42
7.41
7.40
7.39
7.39
7.38
7.32
7.32
7.31
7.31
7.31
7.30
7.26
7.24
7.24
7.23
7.21
7.21
5.83
5.58
5.31
1.00
0.98
1.07
3.18
3.04
1.07
1.00
2.07
2.00
f1 (ppm)

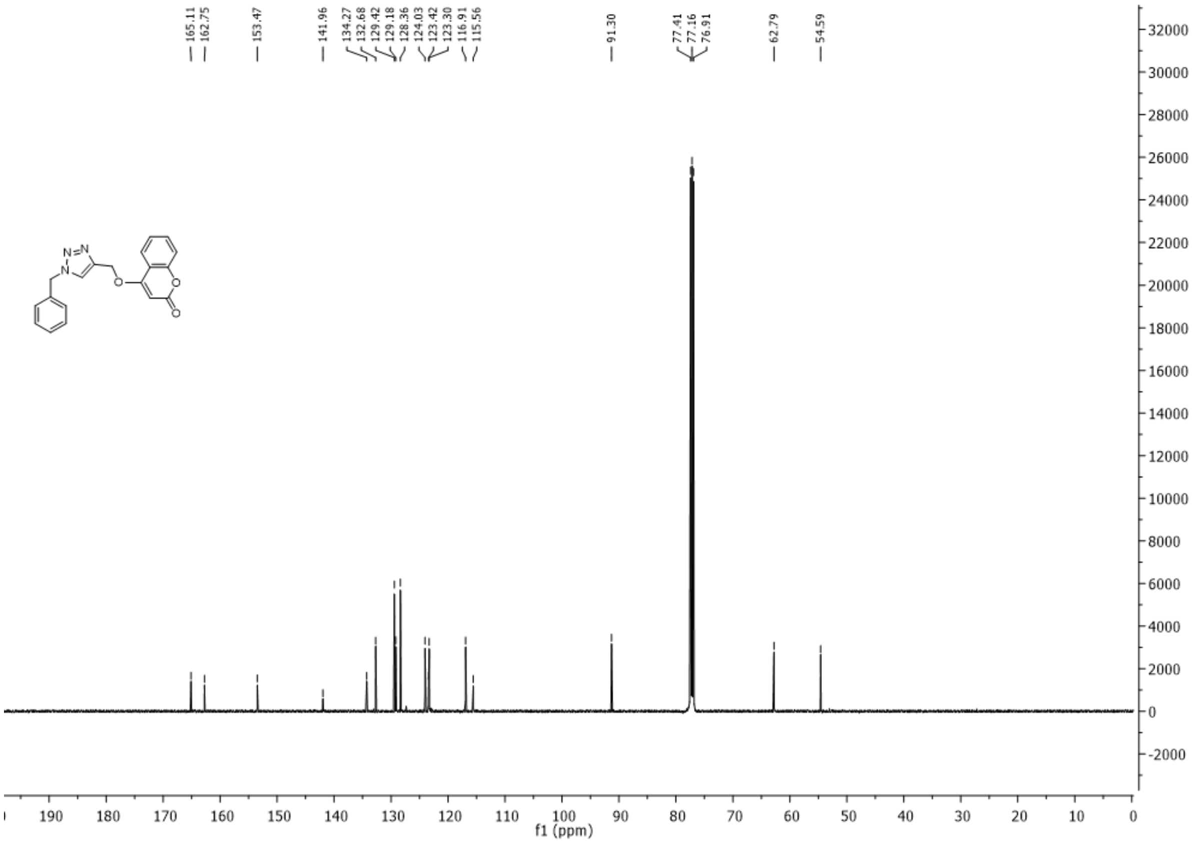
165.11
162.75
153.47
141.96
134.27
132.68
129.42
129.18
128.36
124.03
123.42
123.30
116.91
115.56
91.30
77.41
77.16
76.91
62.79
54.59
f1 (ppm)

Qualitative Compound Report

**Data File**	rr-114 (pos.mode).d	**Sample Name**	rr-114 (pos.mode)
**Sample Type**	Sample	**Position**	Vial 1
**Instrument Name**	QTOF	**User Name**	QTOF-PC\admin
**Acq Method**	ACgroup_new.m	**Acquired Time**	2019-07-10 14:02:19
**IRM Calibration Status**	Success	**DA Method**	szfg123.m
**Comment**			

**Acquisition SW Version**	6200 series TOF/6500 series Q-TOF B.05.00 (B5042.2)

Compound Table

Compound Label	RT	Mass	Abund	Formula	Tgt Mass	Diff (ppm)	MFG Formula	DB Formula
Cpd 1: C19 H15 N3 O3	0.603	333.1133	4995	C19 H15 N3 O3	333.1113	5.91	C19 H15 N3 O3	C19 H15 N3 O3

Compound Label	*m/z*	RT	Algorithm	Mass
Cpd 1: C19 H15 N3 O3	356.1019	0.603	Find By Formula	333.1133

MS Zoomed Spectrum

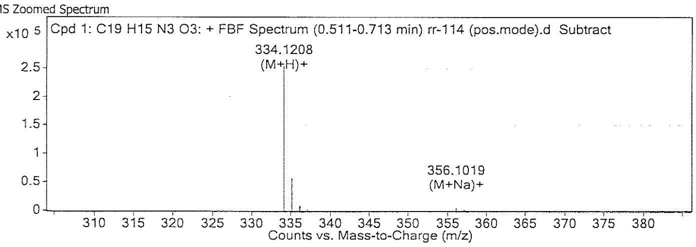

MS Spectrum Peak List

m/z	z	Abund	Formula	Ion
334.1208	1	254268.11	C19H16N3O3	(M+H)+
335.1231	1	55465.5	C19H16N3O3	(M+H)+
336.1253	1	7393.29	C19H16N3O3	(M+H)+
337.1257	1	766.77	C19H16N3O3	(M+H)+
356.1019	1	4995.1	C19H15N3NaO3	(M+Na)+
357.1027	1	1026.74	C19H15N3NaO3	(M+Na)+
358.1047	1	119.39	C19H15N3NaO3	(M+Na)+

--- End Of Report ---

Mass spectrum of 4h

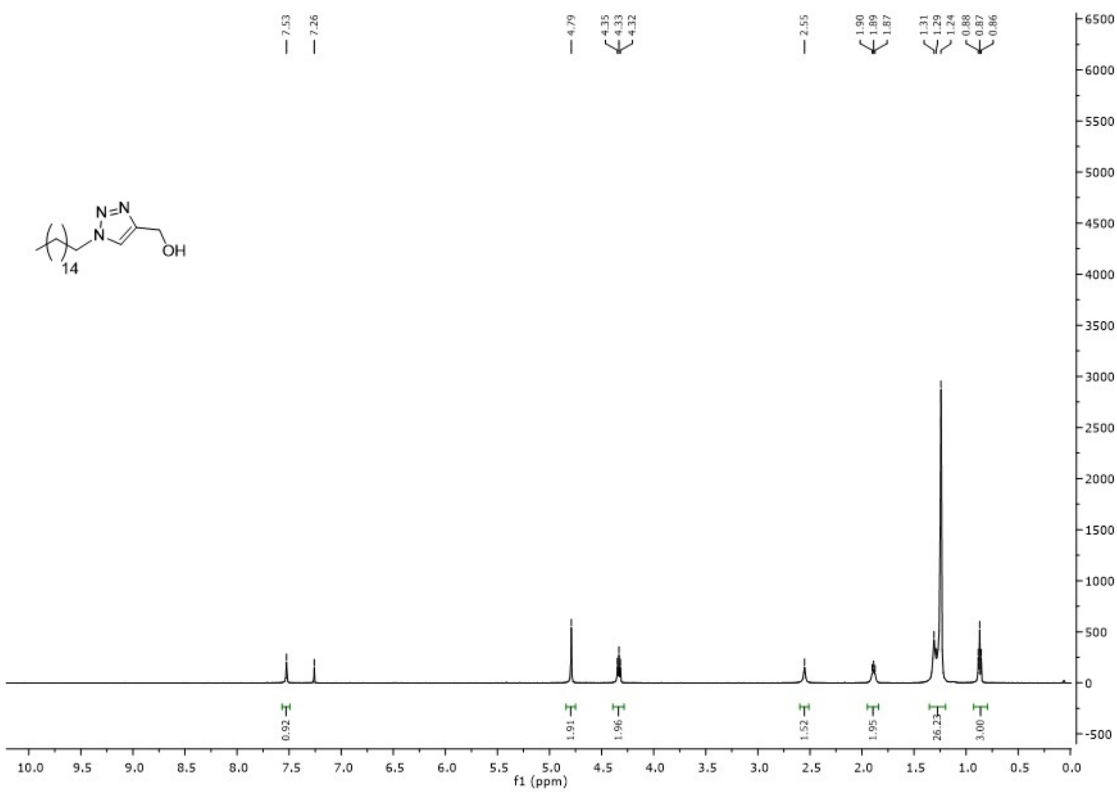
7.53
7.26
4.79
4.35
4.33
4.32
2.55
1.90
1.89
1.87
1.31
1.29
1.24
0.88
0.87
0.86
OH
14
0.92
1.91
1.96
1.52
1.95
26.23
3.00
f1 (ppm)

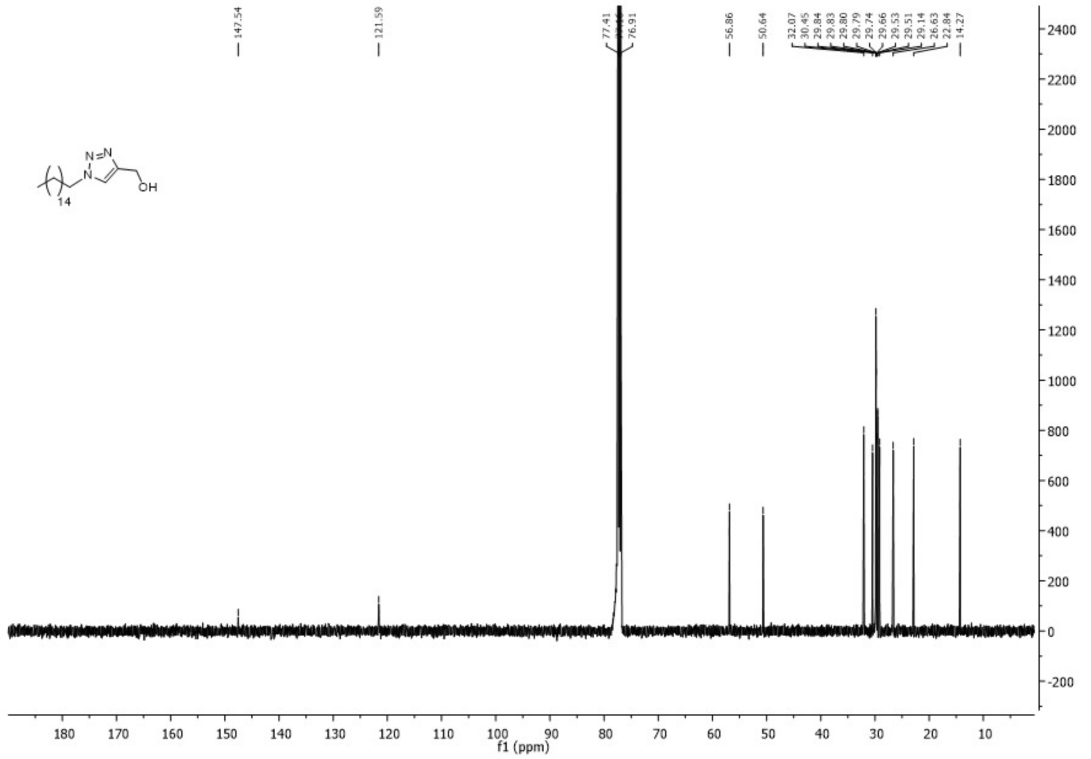
147.54
121.59
77.41
76.91
56.86
50.64
32.07
30.45
29.84
29.83
29.80
29.79
29.74
29.66
29.53
29.51
29.14
26.63
22.84
14.27
OH
14
f1 (ppm)

Qualitative Compound Report

**Data File**	rr-109 (pos.mode).d	**Sample Name**	rr-109 (pos.mode)
**Sample Type**	Sample	**Position**	Vial 1
**Instrument Name**	QTOF	**User Name**	QTOF-PC\admin
**Acq Method**	ACgroup_new.m	**Acquired Time**	2019-07-10 14:19:09
**IRM Calibration Status**	Success	**DA Method**	szfg123.m
**Comment**			

**Acquisition SW Version**	6200 series TOF/6500 series Q-TOF B.05.00 (B5042.2)

Compound Table

Compound Label	RT	Mass	Abund	Formula	Tgt Mass	Diff (ppm)	MFG Formula	DB Formula
Cpd 1: C19 H37 N3 O	5.836	323.2958	7552	C19 H37 N3 O	323.2937	6.53	C19 H37 N3 O	C19 H37 N3 O

Compound Label	*m/z*	RT	Algorithm	Mass
Cpd 1: C19 H37 N3 O	346.283	5.836	Find By Formula	323.2958

MS Zoomed Spectrum

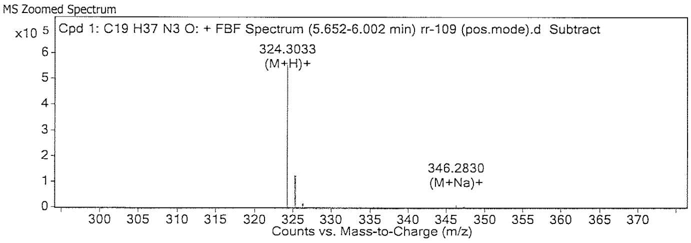

MS Spectrum Peak List

m/z	z	Abund	Formula	Ion
324.3033	1	554835.56	C19H38N3O	(M+H)+
325.3054	1	119526.73	C19H38N3O	(M+H)+
326.3077	1	13634.13	C19H38N3O	(M+H)+
327.3088	1	917.23	C19H38N3O	(M+H)+
346.283	1	7552.32	C19H37N3NaO	(M+Na)+
347.2865	1	1718.72	C19H37N3NaO	(M+Na)+
348.2838	1	419.83	C19H37N3NaO	(M+Na)+

--- End Of Report ---

Mass spectrum of 4i

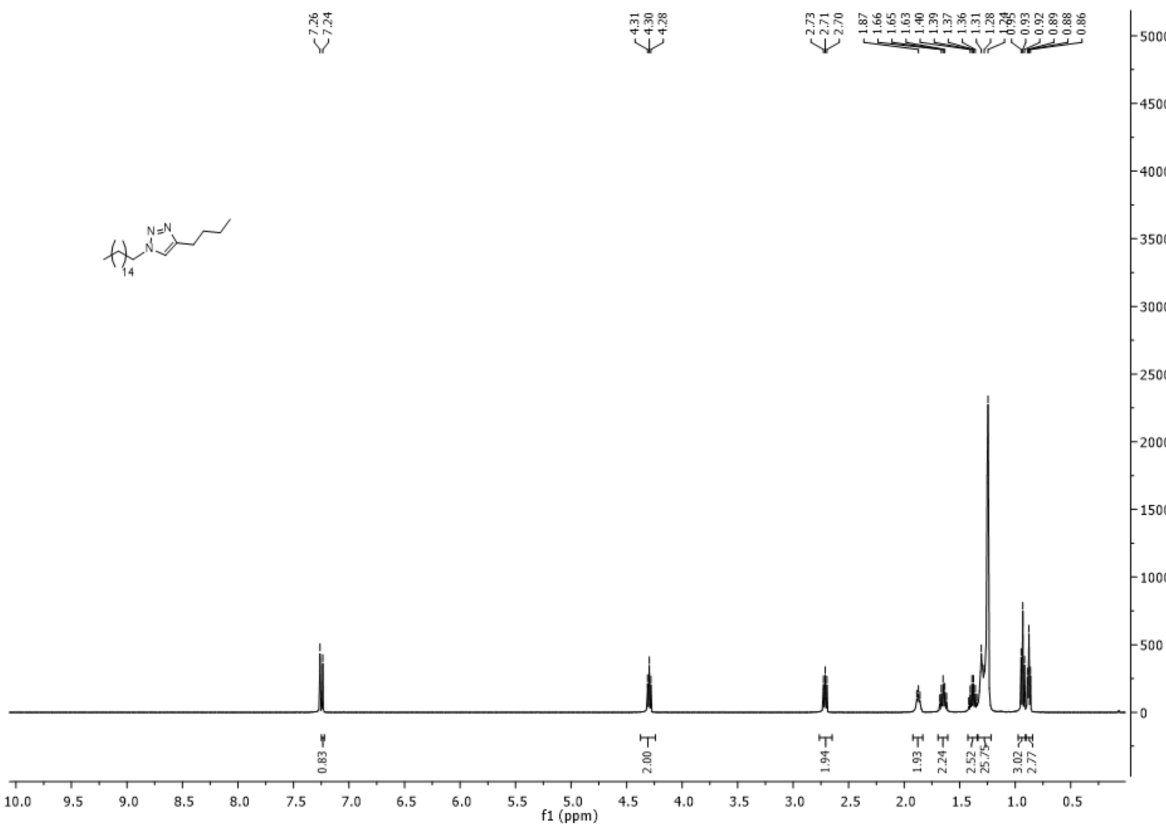
f1 (ppm)

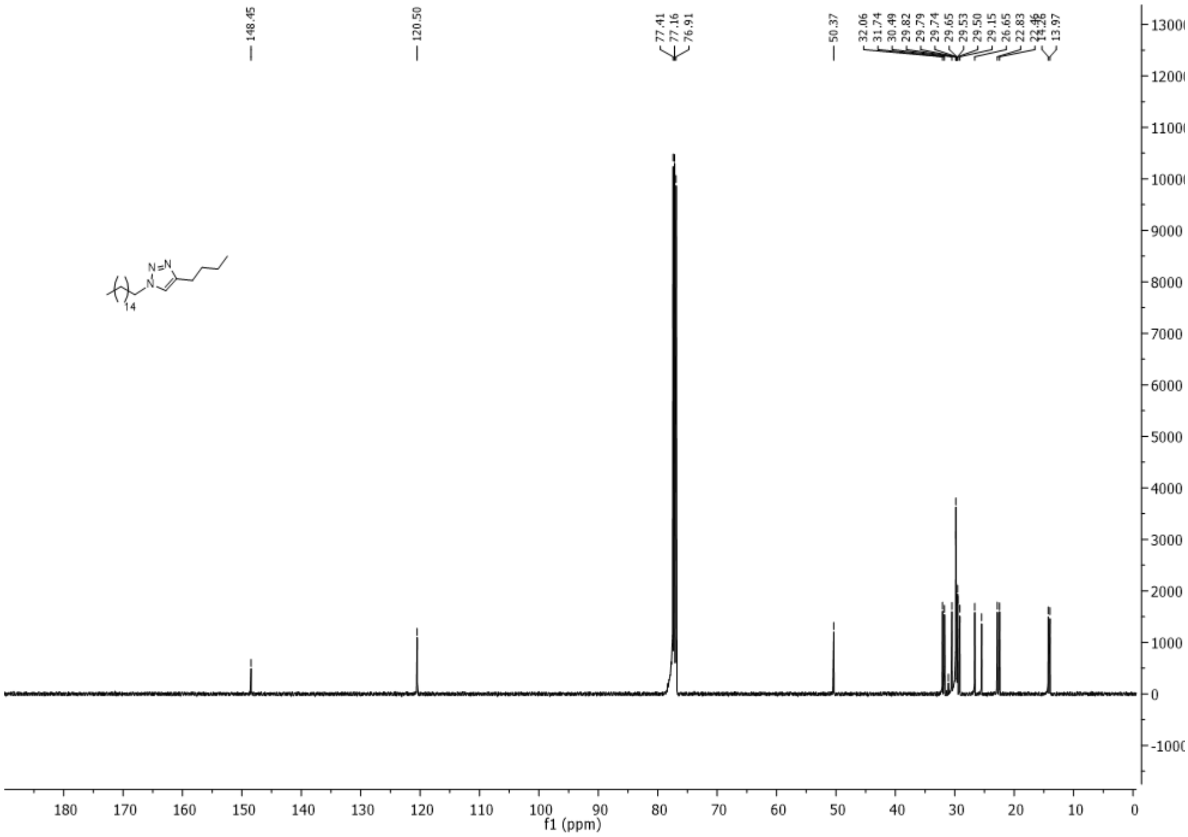
f1 (ppm)

Qualitative Compound Report

**Data File**	rr-110(2).d	**Sample Name**	rr-110(2)
**Sample Type**	Sample	**Position**	vial1
**Instrument Name**	QTOF	**User Name**	QTOF-PC\admin
**Acq Method**	ACgroup_new_no_column.m	**Acquired Time**	2019-06-20 14:51:03
**IRM Calibration Status**	Success	**DA Method**	szfg123.m
**Comment**			

**Acquisition SW Version**	6200 series TOF/6500 series Q-TOF B.05.00 (B5042.2)

Compound Table

Compound Label	RT	Mass	Abund	Formula	Tgt Mass	Diff (ppm)	MFG Formula	DB Formula
Cpd 1: C22 H43 N3	0.09	349.3462	3068792	C22 H43 N3	349.3457	1.42	C22 H43 N3	C22 H43 N3

Compound Label	*m/z*	RT	Algorithm	Mass
Cpd 1: C22 H43 N3	350.3533	0.09	Find By Formula	349.3462

MS Zoomed Spectrum

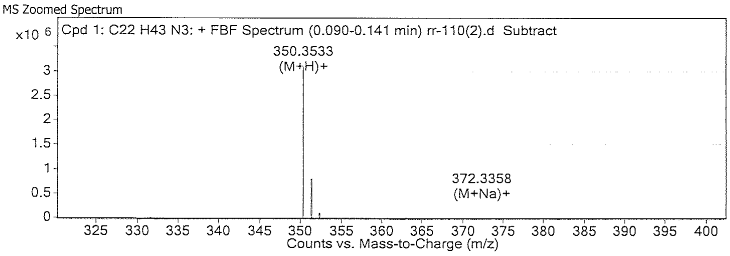

MS Spectrum Peak List

m/z	z	Abund	Formula	Ion
350.3533	1	3068791.75	C22H44N3	(M+H)+
351.3573	1	791230.19	C22H44N3	(M+H)+
352.3599	1	91993.85	C22H44N3	(M+H)+
353.3633	1	6571.12	C22H44N3	(M+H)+
372.3358	1	4725.44	C22H43N3Na	(M+Na)+
373.3388	1	1141.21	C22H43N3Na	(M+Na)+

--- End Of Report ---

Mass spectrum of 4j

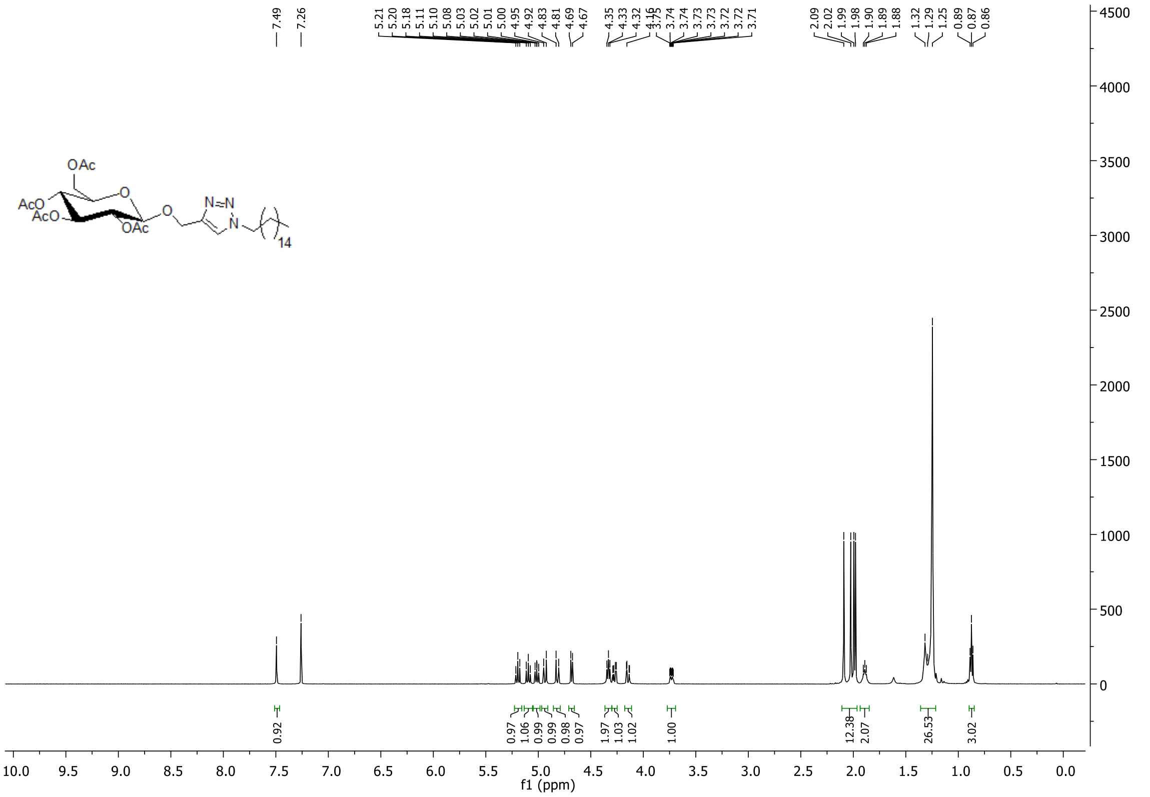

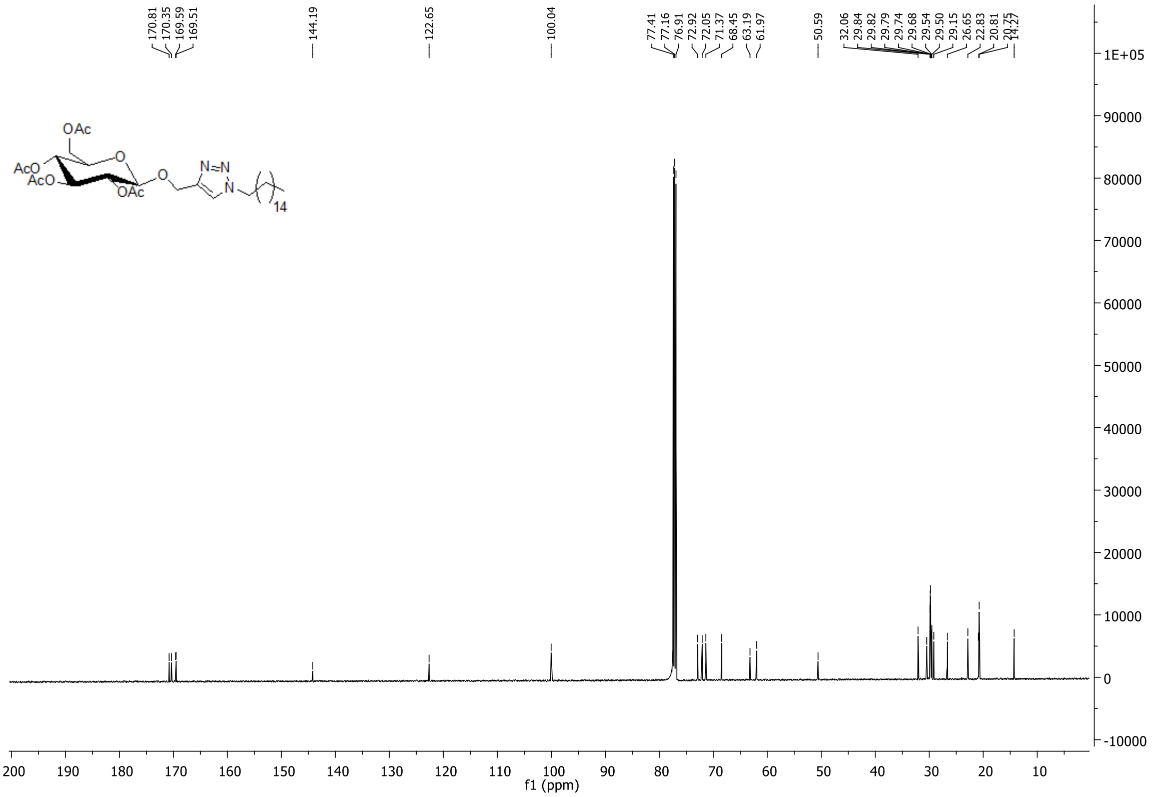

Qualitative Compound Report

**Data File**	rr-110 (pos.mode).d	**Sample Name**	rr-110 (pos.mode)
**Sample Type**	Sample	**Position**	Vial 1
**Instrument Name**	QTOF	**User Name**	QTOF-PC\admin
**Acq Method**	ACgroup_new.m	**Acquired Time**	2019-07-10 14:48:41
**IRM Calibration Status**	Success	**DA Method**	szfg123.m
**Comment**			

**Acquisition SW Version**	6200 series TOF/6500 series Q-TOF B.05.00 (B5042.2)

Compound Table

Compound Label	RT	Mass	Abund	Formula	Tgt Mass	Diff (ppm)	MFG Formula	DB Formula
Cpd 1: C33 H55 N3 O10	4.795	653.3887	812	C33 H55 N3 O10	653.3887	-0.13	C33 H55 N3 O10	C33 H55 N3 O10

Compound Label	*m/z*	RT	Algorithm	Mass
Cpd 1: C33 H55 N3 O10	654.3976	4.795	Find By Formula	653.3887

MS Zoomed Spectrum

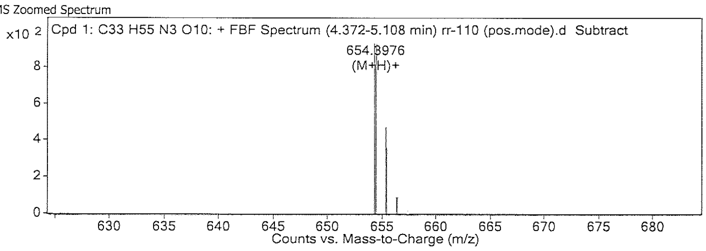

MS Spectrum Peak List

m/z	z	Abund	Formula	Ion
654.3976	1	811.98	C33H56N3O10	(M+H)+
655.3964	1	470.5	C33H56N3O10	(M+H)+
656.4013	1	86.6	C33H56N3O10	(M+H)+

--- End Of Report ---

Mass spectrum of 4k

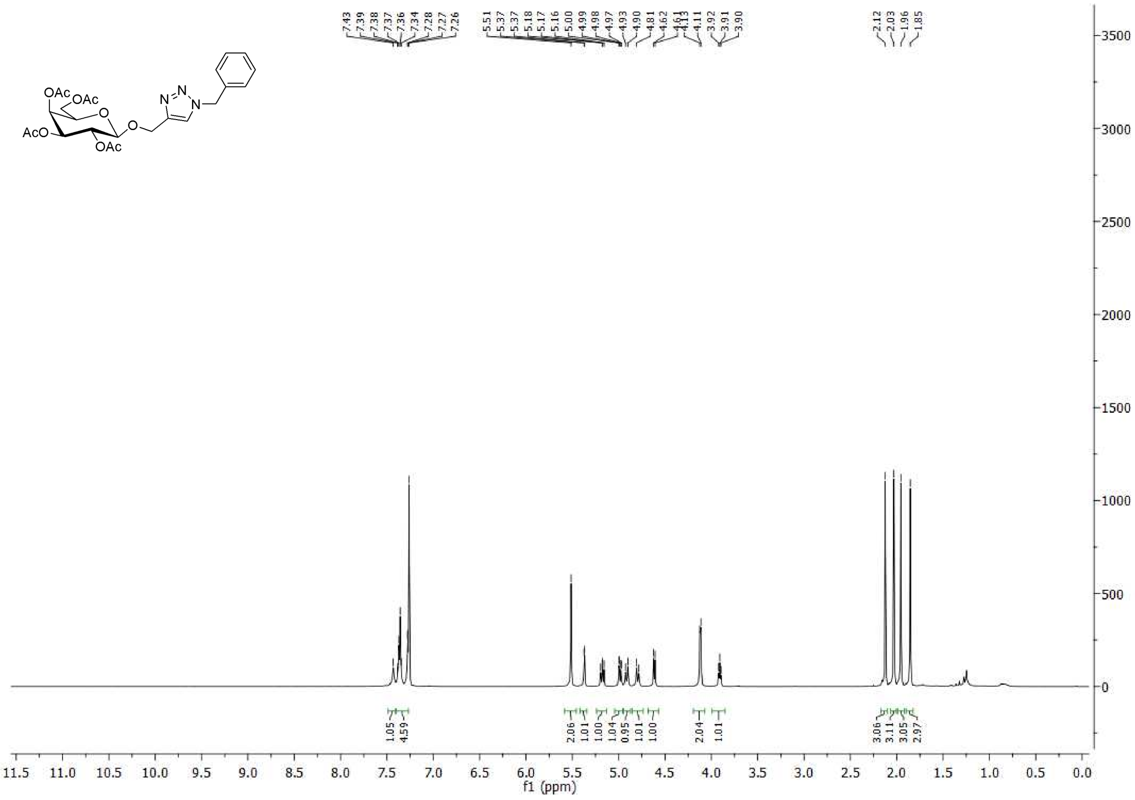
AcO
OAc
OAc
OAc
O
O
N
N
N
7.43
7.39
7.38
7.37
7.36
7.34
7.28
7.27
7.26
5.51
5.37
5.37
5.18
5.17
5.16
5.00
4.99
4.98
4.97
4.93
4.90
4.81
4.62
4.61
4.13
4.11
3.92
3.91
3.90
2.12
2.03
1.96
1.85
1.05
4.59
2.06
1.01
1.00
1.04
0.95
1.01
1.00
2.04
1.01
3.06
3.11
3.05
2.97
f1 (ppm)

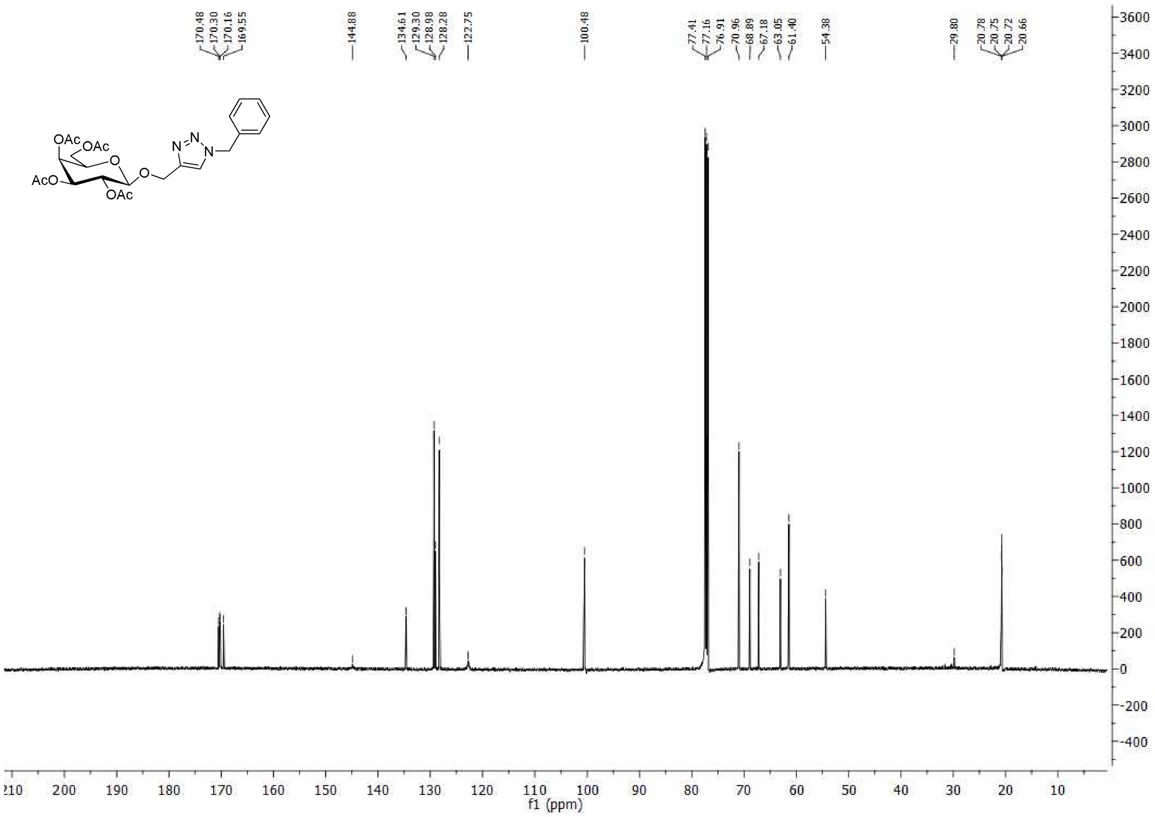

Qualitative Compound Report

**Data File**	rr-1-159.d	**Sample Name**	rr-1-159
**Sample Type**	Sample	**Position**	Vial 1
**Instrument Name**	QTOF	**User Name**	QTOF-PC\admin
**Acq Method**	ACgroup_new.m	**Acquired Time**	2020-07-28 12:36:06
**IRM Calibration Status**	Success	**DA Method**	Default.m
**Comment**			

Acquisition SW Version 6200 series TOF/6500 series Q-TOF B.05.00 (B5042.2)

Compound Table

Compound Label	RT	Mass	Abund	Formula	Tgt Mass	Diff (ppm)	MFG Formula	DB Formula
Cpd 1: C24 H29 N3 O10	1.061	519.1841	1156511	C24 H29 N3 O10	519.1853	-2.27	C24 H29 N3 O10	C24 H29 N3 O10

Compound Label	m/z	RT	Algorithm	Mass
Cpd 1: C24 H29 N3 O10	520.1915	1.061	Find By Formula	519.1841

MS Zoomed Spectrum

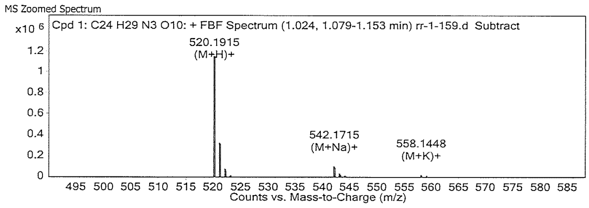

MS Spectrum Peak List

m/z	z	Abund	Formula	Ion
520.1915	1	1156511.13	C24H30N3O10	(M+H)+
521.195	1	316507.34	C24H30N3O10	(M+H)+
522.1971	1	63051.61	C24H30N3O10	(M+H)+
523.1991	1	8767.11	C24H30N3O10	(M+H)+
542.1715	1	96297.91	C24H29N3NaO10	(M+Na)+
543.1745	1	25504.33	C24H29N3NaO10	(M+Na)+
544.1772	1	5465.98	C24H29N3NaO10	(M+Na)+
558.1448	1	14345	C24H29KN3O10	(M+K)+
559.1486	1	4064.02	C24H29KN3O10	(M+K)+
560.1472	1	2088.94	C24H29KN3O10	(M+K)+

--- End Of Report ---

Mass spectrum of 41

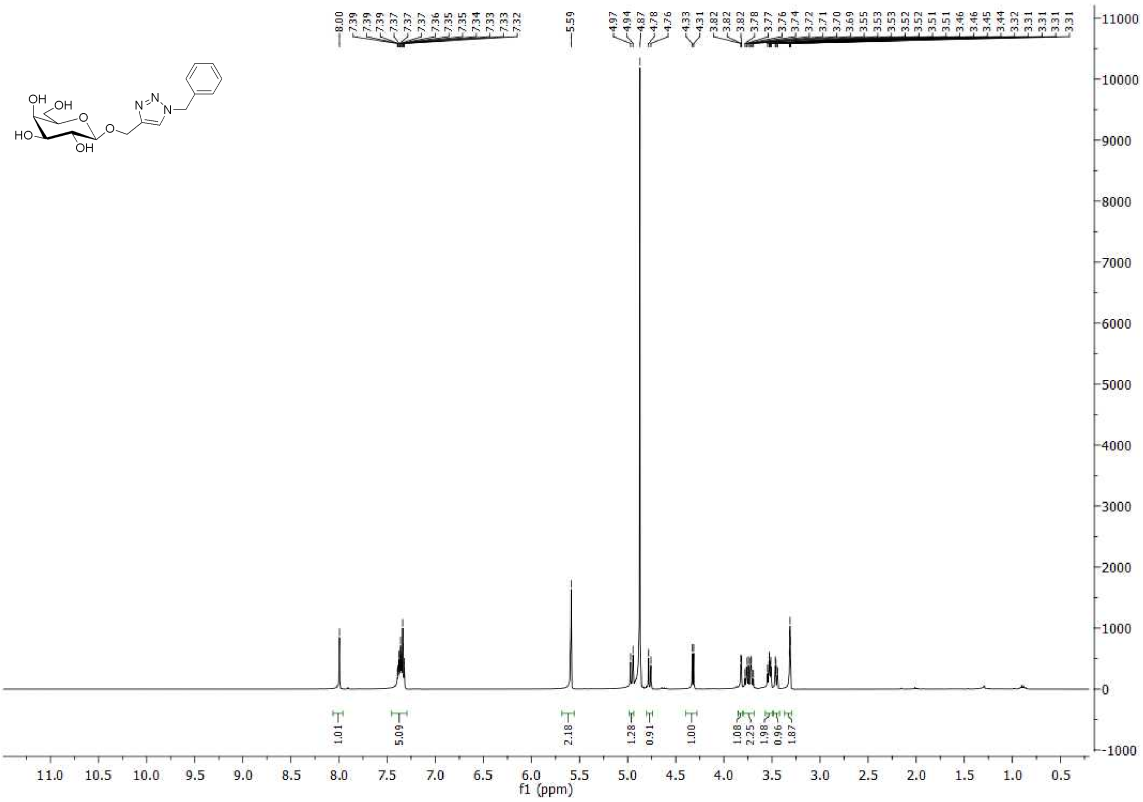
8.00
7.39
7.39
7.39
7.37
7.37
7.37
7.36
7.35
7.35
7.34
7.33
7.33
7.32
5.59
4.97
4.94
4.87
4.78
4.76
4.33
4.31
3.82
3.82
3.78
3.77
3.76
3.74
3.72
3.71
3.70
3.69
3.55
3.53
3.53
3.52
3.52
3.51
3.51
3.46
3.46
3.45
3.44
3.32
3.31
3.31
3.31
3.31
1.01
5.09
2.18
1.28
0.91
1.00
1.08
2.25
1.98
0.96
1.87
f1 (ppm)

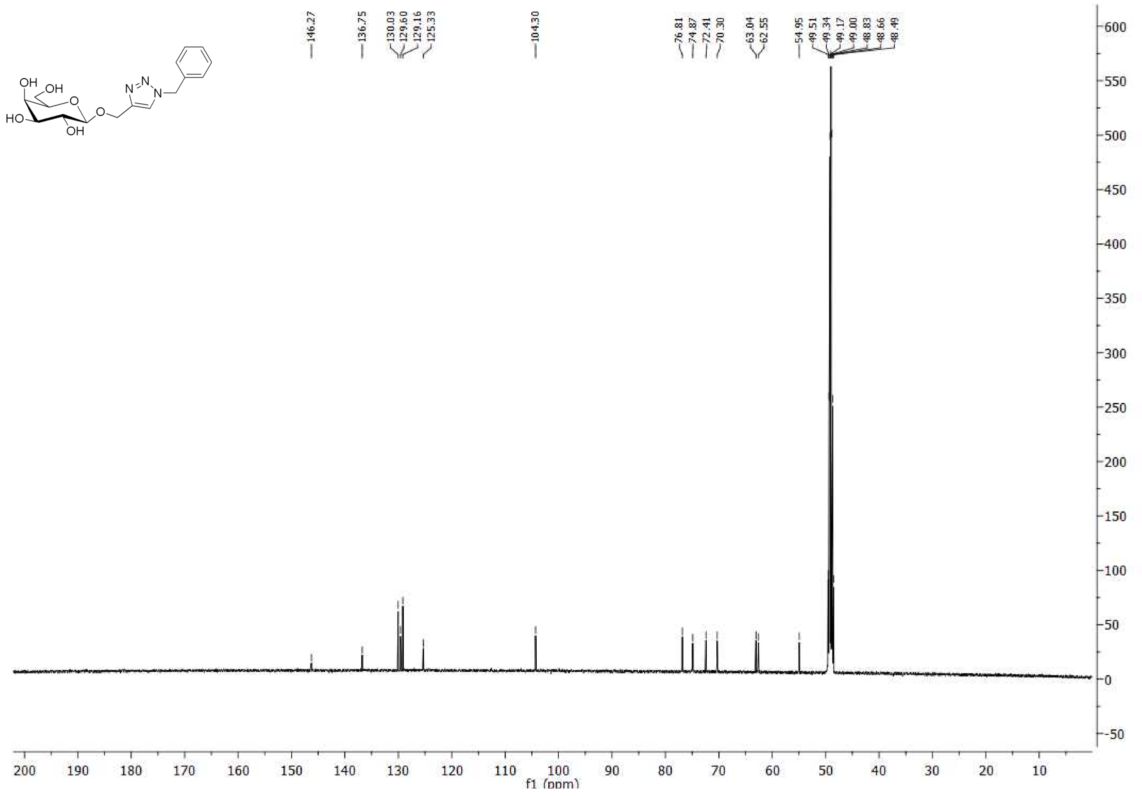
146.27
136.75
130.03
129.60
129.16
125.33
104.30
76.81
74.87
72.41
70.30
63.04
62.55
54.95
49.51
49.34
49.17
49.00
48.83
48.66
48.49
f1 (ppm)

Qualitative Compound Report

**Data File**	rr-1-153.d	**Sample Name**	rr-1-153
**Sample Type**	Sample	**Position**	Vial 1
**Instrument Name**	QTOF	**User Name**	QTOF-PC\admin
**Acq Method**	ACgroup_new.m	**Acquired Time**	2020-07-28 12:43:36
**IRM Calibration Status**	Success	**DA Method**	Default.m
**Comment**			

**Acquisition SW**	6200 series TOF/6500 series
**Version**	Q-TOF B.05.00 (B5042.2)

Compound Table

Compound Label	RT	Mass	Abund	Formula	Tgt Mass	Diff (ppm)	MFG Formula	DB Formula
Cpd 1: C16 H21 N3 O6	0.704	351.1425	415024	C16 H21 N3 O6	351.143	-1.5	C16 H21 N3 O6	C16 H21 N3 O6

Compound Label	*m/z*	RT	Algorithm	Mass
Cpd 1: C16 H21 N3 O6	352.1501	0.704	Find By Formula	351.1425

MS Zoomed Spectrum

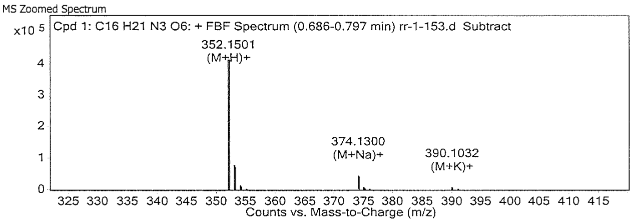

MS Spectrum Peak List

m/z	z	Abund	Formula	Ion
352.1501	1	415023.81	C16H22N3O6	(M+H)+
353.1527	1	75427.71	C16H22N3O6	(M+H)+
354.1549	1	11763.93	C16H22N3O6	(M+H)+
355.1578	1	1286.74	C16H22N3O6	(M+H)+
374.13	1	43671.35	C16H21N3NaO6	(M+Na)+
375.1336	1	8625.22	C16H21N3NaO6	(M+Na)+
376.135	1	1243.22	C16H21N3NaO6	(M+Na)+
390.1032	1	6634.37	C16H21KN3O6	(M+K)+
391.1071	1	1296.01	C16H21KN3O6	(M+K)+
392.1027	1	701.81	C16H21KN3O6	(M+K)+

--- End Of Report ---

Mass spectrum of 4m

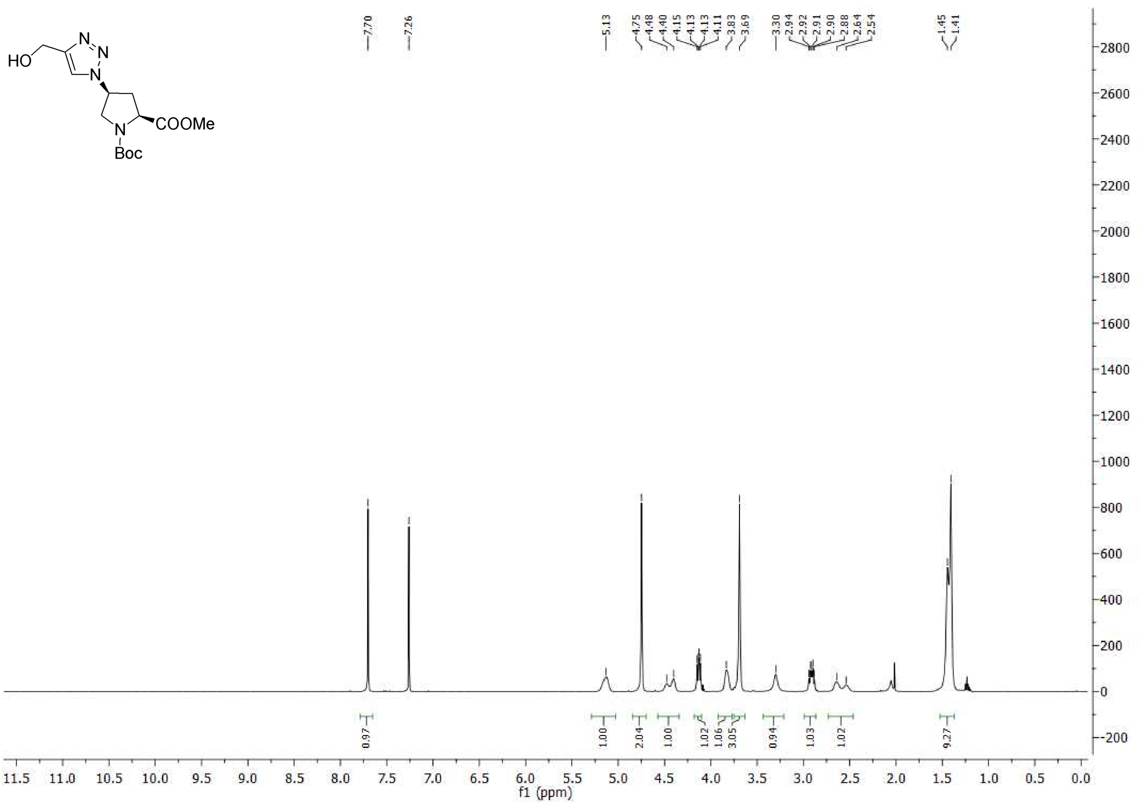
HO
N
N
N
COOMe
N
Boc
7.70
7.26
5.13
4.75
4.48
4.40
4.15
4.13
4.13
4.11
3.83
3.69
3.30
2.94
2.92
2.91
2.90
2.88
2.64
2.54
1.45
1.41
0.97
1.00
2.04
1.00
1.02
1.06
3.05
0.94
1.03
1.02
9.27
f1 (ppm)

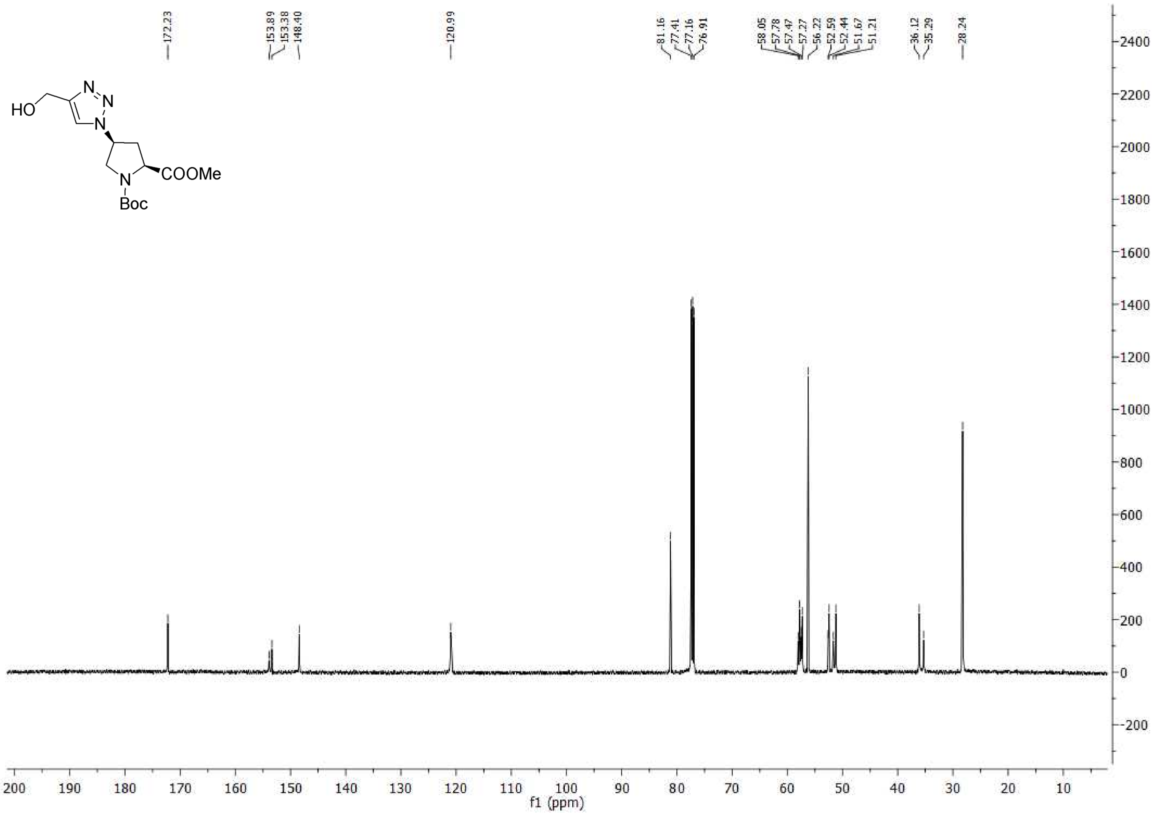
HO
N
N
N
COOMe
N
Boc
172.23
153.89
153.38
148.40
120.99
81.16
77.41
77.16
76.91
58.05
57.78
57.47
57.27
56.22
52.59
52.44
51.67
51.21
36.12
35.29
28.24
f1 (ppm)

Qualitative Compound Report

**Data File**	rr-2-75(c).d	**Sample Name**	rr-2-75(c)
**Sample Type**	Sample	**Position**	Vial 1
**Instrument Name**	QTOF	**User Name**	QTOF-PC\admin
**Acq Method**	ACgroup_new.m	**Acquired Time**	2020-07-28 12:07:49
**IRM Calibration Status**	Success	**DA Method**	Default.m
**Comment**			

Acquisition SW Version 6200 series TOF/6500 series Q-TOF B.05.00 (B5042.2)

Compound Table

Compound Label	RT	Mass	Abund	Formula	Tgt Mass	Diff (ppm)	MFG Formula	DB Formula
Cpd 1: C14 H22 N4 O5	0.797	326.1578	1201146	C14 H22 N4 O5	326.159	-3.72	C14 H22 N4 O5	C14 H22 N4 O5

Compound Label	*m/z*	RT	Algorithm	Mass
Cpd 1: C14 H22 N4 O5	327.1651	0.797	Find By Formula	326.1578

MS Zoomed Spectrum

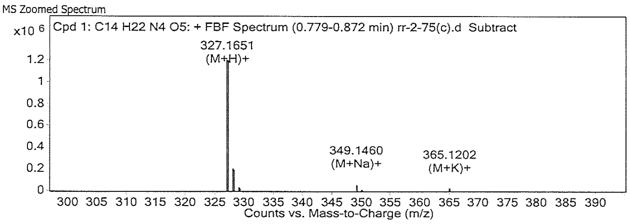

MS Spectrum Peak List

m/z	z	Abund	Formula	Ion
327.1651	1	1201146.13	C14H23N4O5	(M+H)+
328.1685	1	202500.8	C14H23N4O5	(M+H)+
329.1703	1	28493.19	C14H23N4O5	(M+H)+
330.1729	1	2782.93	C14H23N4O5	(M+H)+
349.146	1	57636.44	C14H22N4NaO5	(M+Na)+
350.1487	1	9649.43	C14H22N4NaO5	(M+Na)+
351.1511	1	1344.52	C14H22N4NaO5	(M+Na)+
365.1202	1	18730.93	C14H22KN4O5	(M+K)+
366.1231	1	3321.46	C14H22KN4O5	(M+K)+
367.1213	1	1822.26	C14H22KN4O5	(M+K)+

--- End Of Report ---

Mass spectrum of 4n
